# Engineered bidirectional promoters enable rapid multi-gene co-expression optimization

**DOI:** 10.1038/s41467-018-05915-w

**Published:** 2018-09-04

**Authors:** Thomas Vogl, Thomas Kickenweiz, Julia Pitzer, Lukas Sturmberger, Astrid Weninger, Bradley W. Biggs, Eva-Maria Köhler, Armin Baumschlager, Jasmin Elgin Fischer, Patrick Hyden, Marlies Wagner, Martina Baumann, Nicole Borth, Martina Geier, Parayil Kumaran Ajikumar, Anton Glieder

**Affiliations:** 1 Institute of Molecular Biotechnology, NAWI Graz, Graz University of Technology, Petersgasse 14, 8010 Graz, Austria; 2 Austrian Centre of Industrial Biotechnology (ACIB GmbH), Petersgasse 14, 8010 Graz, Austria; 3 Manus Biosynthesis, 1030 Massachusetts Avenue, Suite 300, Cambridge, MA 02138 USA; 4 Austrian Centre of Industrial Biotechnology (ACIB GmbH), Muthgasse 11, 1190 Vienna, Austria; 5 Department of Biotechnology, University of Natural Resources and Life Sciences, Muthgasse 18, 1190 Vienna, Austria; 6 Present Address: Department of Computer Science and Applied Mathematics, Weizmann Institute of Science, 76100 Rehovot, Israel; 7 Present Address: Department of Chemical and Biological Engineering, Northwestern University, Evanston, IL 60208 USA; 8 Present Address: Department of Biosystems Science and Engineering, ETH Zürich, Mattenstrasse 26, 4058 Basel, Switzerland

## Abstract

Numerous synthetic biology endeavors require well-tuned co-expression of functional components for success. Classically, monodirectional promoters (MDPs) have been used for such applications, but MDPs are limited in terms of multi-gene co-expression capabilities. Consequently, there is a pressing need for new tools with improved flexibility in terms of genetic circuit design, metabolic pathway assembly, and optimization. Here, motivated by nature’s use of bidirectional promoters (BDPs) as a solution for efficient gene co-expression, we generate a library of 168 synthetic BDPs in the yeast *Komagataella phaffii* (syn. *Pichia pastoris*), leveraging naturally occurring BDPs as a parts repository. This library of synthetic BDPs allows for rapid screening of diverse expression profiles and ratios to optimize gene co-expression, including for metabolic pathways (taxadiene, β-carotene). The modular design strategies applied for creating the BDP library could be relevant in other eukaryotic hosts, enabling a myriad of metabolic engineering and synthetic biology applications.

## Abstract (web summary)

Classic monodirectional promoters are of limited use for multiple gene co-expression. Here the authors generate a library of 168 bidirectional promoters for the yeast *K. phaffii* (syn*. P. pastoris*) with diverse expression profiles to optimize metabolic pathway design.

## Introduction

Efficient and well-tuned co-expression of multiple genes is a common challenge in metabolic engineering and synthetic biology, wherein protein components must be optimized in terms of cumulative expression, expression ratios, and regulation^1–4^. When co-expressing multiple proteins, not only their ratios to each other but also their total (cumulative) amounts summed together matter. Too excessive loads of heterologous proteins may overburden the cellular machinery of recombinant expression hosts. Hence, in addition to balancing the proteins relative to each other, their total (cumulative) expression strength needs to be adjusted. Else, burdensome overexpression of proteins or accumulation of toxic intermediate metabolites may prove detrimental to the cellular host and undermine engineering goals. One remedy has been to restrict protein overexpression to only certain times through dynamic or regulated transcription (inducibility)^1^. A second is to balance pathway expression to prevent toxic metabolite accumulation^3,5^, mimicking natural pathways’ balanced protein stoichiometries^6^.

Though effective, these methods’ ability to improve pathway performance by controlling gene expression is constrained to the tools available. To date, and especially in the context of eukaryotic microbes, this has primarily been restricted to monodirectional promoters (MDPs), which possess limits in terms of cloning and final pathway construction. Interestingly, nature has encountered similar gene expression challenges, developing its own set of solutions. This includes the use of bidirectional promoters (BDPs) to expand expression flexibility, exemplified by multi-subunit proteins such as histone-forming nucleosomes^7^.

Natural BDPs (nBDPs) and divergent transcription have been characterized in all model organisms^8–18^, with RNAseq studies even indicating that eukaryotic promoters are intrinsically bidirectional^9,11,13,19^. Moreover, nBDPs with non-cryptic expression in both orientations frequently co-regulate functionally related genes^20,21^. Inspired by these circuits, biological engineers have recently utilized BDPs to improve designs for gene co-expression in *Escherichia coli*^22^, *Saccharomyces cerevisiae*^23^, plants^24^, and mammals^25,26^. These studies offer promise, but larger sets of readily available BDPs remain limited, and the reported strategies have lacked generalizability. To our knowledge, *S. cerevisiae*’s less than dozen BDPs represent the largest collection^23^ and do not provide the desired spectrum of different expression ratios or consecutive induction.

BDPs offer the ability to dramatically improve pathway design, with applicability in numerous and even emerging hosts. In contrast to monodirectional expression cassettes in tandem, bidirectional cloning offers a simple and quick solution to identify optimal promoter contributions for co-expression in a single cloning expression screening experiment. But, for BDPs to be fully utilized a much larger set must be engineered, with the ideal library representing different expression levels and regulatory profiles varied per expression direction. Such a library could halve cloning junctions compared to conventional MDPs, facilitating rapid assembly of combinatorial libraries that efficiently explore broad expression landscapes. In addition, development of tools such as these could help to unlock the use of emerging hosts, such as *Pichia pastoris* (syn. *Komagataella phaffii*), which have the potential not only for industrial and pharmaceutical enzyme production but also food and diary protein production and as chemical factories^27^.

Here we generate a collection of 168 BDPs in the methylotrophic yeast *P. pastoris*, using its natural histone promoters as an engineering template. Our library covers a 79-fold range of cumulative expression, has variable expression ratios ranging from parity to a 61-fold difference between sides, and combines different regulatory profiles per side including the possibility for consecutive induction. The utility of these BDPs is demonstrated through the optimization of multi-gene co-expression, and the conserved nature of the framework histone promoters suggests the generalizability of this approach for other eukaryotes.

## Results and discussion

### Expression capabilities and limitations of natural BDPs

**Fig. 1:**
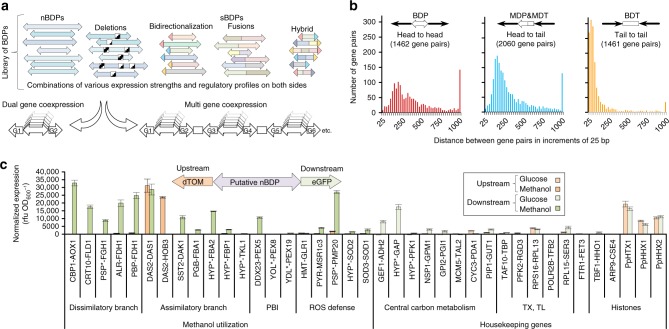
A library of bidirectional promoters (BDPs) for gene co-expression fine-tuning and bidirectional histone promoters are among the few strong *P. pastoris* nBDPs. **a** A library of diversely regulated natural and synthetic BDPs (nBDPs and sBDPs) covering a wide range of regulatory profiles facilitates optimization of dual gene co-expression and the assembly of multi-gene co-expression cassettes (Supplementary Fig. 5, Supplementary Note 6). **b** The *P. pastoris* genome harbors 1462 putative nBDPs (gene pairs in divergent head-to-head orientation, Supplementary Data 1, Supplementary Note 1). The distribution of distances between gene pairs is shown in 25 bp intervals. The last bar indicates gene pairs with an intragenic distance >1000 bp. Also convergent tail-to-tail gene pairs (forming putative bidirectional transcription terminators, BDTs) and head-to-tail/tail-to-head gene pairs flanking a monodirectional promoter (MDP) and a monodirectional terminator (MDT) are shown. Genes are illustrated as bold single-line arrows, promoters as filled arrows, terminators as rectangles. **c** The natural bidirectional *DAS1-DAS2* promoter is the only methanol-inducible *P. pastoris* promoter^30^ showing strong reporter gene fluorescence on both sides and histone promoters are the strongest nBDPs of several housekeeping gene pairs tested in *P. pastoris*. All strains were grown on glucose media for 60 h and MUT promoters subsequently induced with methanol for 48 h (for MUT promoters measurements after growth on methanol, for housekeeping genes on glucose are shown, see Supplementary Data 2 for the exact values). The promoters were screened with a single reporter gene in both orientations and bidirectional expression confirmed using two FPs (normalization factor used as determined in Supplementary Fig. 1). Gene names denoted with an asterisk (*) were shortened and are provided in Supplementary Data 2. Mean values and standard deviations of biological quadruplicates are shown. PBI peroxisome biogenesis and import, ROS reactive oxygen species, TX,TL transcription, translation

Our study began by searching for nBDPs that might satisfy various engineering needs (Fig. 1a), targeting our search to the yeast *P. pastoris*. Long favored as a host for heterologous protein production^28^, *P. pastoris* has recently emerged as a promising chassis for metabolic engineering applications owing to its growth to high cell densities and its excellent protein expression capabilities^29^. In addition, its methanol utilization (MUT) pathway represents one of the largest sets of tightly co-regulated genes in nature, offering transcriptional repression via glucose and inducibility via methanol^30^, making it an ideal target for BDP mining. Bioinformatics approaches (Supplementary Data 1, Supplementary Note 1) identified 1462 putative BDPs in *P. pastoris*’ genome (Fig. 1b), with a subset of 40 BDPs selected for detailed characterization due to their expected high expression as housekeeping genes or previous application as MDPs (Fig. 1c, see Supplementary Data 2 for a list of the promoters tested).

All putative MUT pathway^30^ and housekeeping gene nBDPs were tested to identify potential regulated and constitutive promoters, respectively. Our promoter screening involved green and red fluorescent protein (FP) reporters (Fig. 1c), normalized with respect to their different relative fluorescence units (rfu), which vary, due to their dependence on the specific quantum yields of the FPs and spectrometer settings, to allow direct comparison of the two promoter sides in our experimental setting (Supplementary Fig. 1, Supplementary Note 2). This normalization factor was applied to all promoter measurements reported in this work. Among MUT promoters, only the *DAS1*-*DAS2* promoter (*P*_*DAS1-DAS2*_) showed strong expression on both sides, matching the most frequently used monodirectional *AOX1* (*alcohol oxidase 1*) promoter, concurring with a previous study (ref. ^30^ and Supplementary Fig. 2a, b, Supplementary Note 3). Other MUT promoters showed only strong monodirectional expression (Fig. 1c). Several putative nBDPs of housekeeping genes showed detectable expression on both sides but weaker than the classical and most frequently applied monodirectional *GAP* (*glyceraldehyde-3-phosphate-dehydrogenase*) promoter (*P*_*GAP*_), one of the strongest constitutive promoter in *P. pastoris*^31^, which was used as a benchmark (Fig. 1c). Though the majority of nBDPs mined provided limited engineering applicability, the histone promoters (*P*_*HTX1*_, *P*_*HHX1*_, and *P*_*HHX2*_) showed promise due to their equally strong expression on both sides, matching (Fig. 1c) the *P*_*GAP*_ benchmark during growth on glucose as a carbon source.

### Bidirectional histone promoters as useful parts repository

Based on the results from the nBDPs screening (Fig. 1c), we focused subsequent engineering efforts on the three bidirectional histone promoters *P*_*HTX1*_, *P*_*HHX1*_, and *P*_*HHX2*_, where *HTX* refers to the BDP at the *HTA+HTB* locus and *HHX* represents *HHT–HHF*. These promoters regulate the expression ratios of highly conserved multimeric histone proteins, which are required for packaging DNA into chromatin^7^. They are required to be produced in equimolar amounts in the cell and evolutionary conserved BDPs control these ratios. Note that *P. pastoris* contains in contrast to *S. cerevisiae*^7^ only a single *HTA+HTB* locus (*HTX1*) and two *HHT+HHF* loci (*HHX1*, *HHX2)*.

The function, structure, involvement in gene regulation, and modifications of histones have been extensively investigated in several model organisms, with an emphasis on the cell-cycle-regulated expression of histone promoters^7,32^. Histone promoter have even been utilized to drive heterologous gene expression in fungi^33,34^ and plants^35^, but these studies focused solely on monodirectional expression from histone promoters without evaluating their bidirectional potential.

**Fig. 2:**
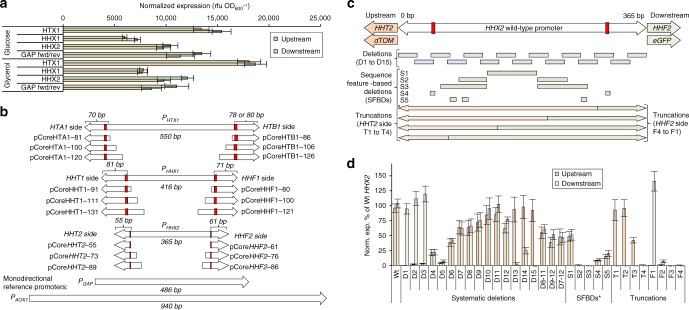
Natural bidirectional histone BDPs as promoter engineering framework in *P. pastoris*. **a** The *HTX1*, *HHX1*, and *HHX2* promoters match (on glucose) or even exceed (on glycerol) the monodirectional *P*_*GAP*_ promoter. Reporter protein fluorescence of the bidirectional *HTX1*, *HHX1*, and *HHX2* promoters in comparison to the strong, monodirectional *GAP* reference promoter in *P. pastoris*. Cells were grown for 60 h on 1% (w/v) glucose or glycerol in 96-well plates. *P*_*GAP*_ was cloned in forward (fwd) and reverse (rev) orientation and is hence not bidirectional. The reporter protein fluorescence is normalized per biomass (determined by OD_600_ measurements) to rule out effects of different biomass yields between the carbon sources. **b** Bidirectional histone promoters are short compared to the commonly used monodirectional *GAP* and *AOX1* promoters (all elements are drawn in the same scale). The histone promoters contain TATA boxes (red rectangle highlighting the yeast TATA box consensus sequence TATAWAWR^37^) and feature exceptionally short core promoters (pCore… and lengths indicated) useful as parts repository for promoter engineering (Figs. 3a,  4). **c**, **d** Owing to their short length, the *P. pastoris* histone BDPs are easily amenable to promoter engineering as exemplified with the *HHX2* promoter. Systematic deletions and truncations of the *P*_*HHX2*_ offer shortened variants with altered cumulative expression levels and ratios. **c** A schematic on the sequence variants is shown (Supplementary Data 2 for exact positions). TATA boxes are denoted by red rectangles. **d** Expression levels after growth for 60 h on glucose are shown. *SFBD sequence feature-based deletions (i.e., AT/GC-rich regions and TATA boxes). In **a**, **d**, mean values and standard deviations of normalized (using the normalization factor calculated in Supplementary Fig. 1) reporter protein fluorescence measurements of biological quadruplicates grown on the respective carbon sources are shown

For our studies, because *P. pastoris* reaches higher specific growth rates and biomass on glycerol compared to glucose^36^, we tested the histone BDPs on both carbons sources. The monodirectional *P*_*GAP*_ benchmark performed better on glucose than glycerol^31^. However, the histone BPDs performed better on glycerol and even outperformed the *P*_*GAP*_ benchmark by up to 1.6-fold (Fig. 2a).

Notably, the bidirectional *P. pastoris* histone promoters condense the regulatory elements needed for strong bidirectional expression compared to monodirectional benchmark promoters (Fig. 2b). This is exemplified in the length of these promoters (365–550 bp) compared to the monodirectional *P*_*GAP*_ (486 bp) and *P*_*AOX1*_ (940 bp). Nonetheless, both sides of the BDPs reached expression levels comparable to MDPs, reflected by a higher expression strength per promoter length (discussed in greater detailed below).

Noticeably all *P. pastoris* histone promoters contain clear TATA box motifs^37^ (Fig. 2b), meaning they are grouped with a class of yeast promoters that rely on TATA-binding protein to initiate transcription instead of alternative factors^38^. TATA box-containing promoters are typically tightly regulated and involved with cellular stress response genes^38^, including with *P. pastoris* MUT genes^30^, whereas TATA-less promoters are typically constitutively active^38^. Hence, the TATA boxes in the histone promoters concur with their tight cell cycle-associated expression^7^.

Using the TATA boxes as a hallmark for determining the core promoter length, we observed exceptionally short core promoters in all histone BDPs (55–81 bp, compared to 160 bp in case of the well-studied *P*_*AOX1*_^39^). Core promoters are the basic region needed for transcription initiation and bound by general transcription factors (TFs) and RNA polymerase II (RNAPII). It is worth nothing that histone core promoter sequences contain the 5′ untranslated region (5′ UTRs) of the natural histone mRNAs, as these cannot easily be functionally separated from the core promoter^40,41^. Regardless of this complication, the short core promoters/5′ UTRs identified here are desirable tools for promoter engineering as they can be simply provided on PCR primers^39–41^. Concurringly, these short histone core promoters turned out to be an excellent repository of parts for promoter bidirectionalization and the creation of synthetic hybrid promoters.

### Creation of BDPs with varied expression strength

**Fig. 3:**
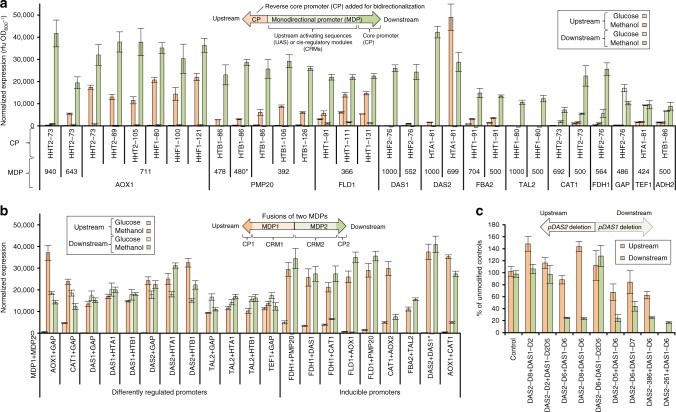
Modular design strategies of synthetic bidirectional promoters (sBDPs) in *P. pastoris* via bidirectionalization and fusions of MDPs yield sBDPs extending the repertoire of ratios and regulatory profiles. **a** Bidirectionalization of MDPs by addition of core promoters (Fig. 2b) yielded functional BDPs in most cases, but few designs gave high expression. The core promoters (CPs) indicated were fused to the indicated MDPs. The length of the MDPs is given in bp, selection criteria are outlined in Supplementary Note 4. Asterisk (*): In case of the *PMP20* promoter, slightly varying sequences from the CBS7435 and the GS115 strain were tested (Supplementary Note 4). Strains were grown on glucose media for 60 h and subsequently induced with methanol for 48 h. **b** Fusions of differently regulated MDPs yield BDPs with different regulatory profiles on each side. Fusions of methanol-inducible MDPs provide a set of strong, tightly regulated, sequence-diversified BDPs allowing co-expression of up to 10 genes without reusing any sequence (Supplementary Note 4 and Supplementary Table 1 for details on the MDPs). For *P*_*HTA1*_ and *P*_*HTB1*_, the truncated versions shown in Fig. 2b and Supplementary Fig. 4 were used. Asterisk (*): Only the fusion of *P*_*DAS2-699*_+*P*_*DAS1-552*_ is shown, for additional comparisons see Supplementary Fig. 2. **c** Fusing deletion variants of *P*_*DAS1*_ and *P*_*DAS2*_ offers strong inducible BDPs with different expression ratios demonstrating that variants of MDPs can be combined into BDPs maintaining their properties on each side. The rationale for the selection of the deletions in *P*_*DAS1*_ and *P*_*DAS2*_ and the measurements of the separate promoters are shown and explained in Supplementary Fig. 2/Supplementary Note 3. Fluorescence was measured after 48 h methanol induction and shown as the percentage of the unmodified fusion promoter (*P*_*DAS2-1000*_+*P*_*DAS1-1000*_). The bidirectionalized and fusion BDPs maintained the regulatory modes of the respective MDPs^30,31^: methanol inducible and tightly glucose/glycerol repressed (*P*_*AOX1*_, *P*_*PMP20*_, *P*_*DAS1/2*_ [and deletion variants thereof], *P*_*FBA2*_, *P*_*TAL2*_, *P*_*AOX2*_), derepressed and methanol inducible (*P*_*CAT1*_, *P*_*FLD1*_, *P*_*FDH1*_), and constitutive (*P*_*GAP*_, *P*_*TEF1*_, *P*_*ADH2*_, *P*_*HTX1 [HTA1-HTB1]*_). In all panels of this figure, mean values and standard deviations of normalized (using the normalization factor calculated in Supplementary Fig. 1) reporter protein fluorescence measurements of biological quadruplicates grown on the respective carbon sources are shown (see Supplementary Data 2 for the exact values)

Their strong bidirectional expression and short length provided opportunity to use the histone BDPs as a template for mutagenesis strategies^42^ to create a library of variants with greater expression flexibility. To expand the expression capabilities of the natural histone BDPs beyond only a fixed ratio and cumulative expression strength, we utilized truncation and deletion strategies of *P*_*HHX2*_ (Fig. 2c, d) to construct a synthetic BDP (sBDP) library with diversified expression strengths and ratios (Fig. 3c, d). Interestingly, removing the core promoter from one side of a BDP (Fig. 2c, d, Supplementary Fig. 4, Supplementary Note 5) increased monodirectional expression on the other side up to 1.5-fold, hinting a regulatory model in which two core promoters are competing for transcription initiation by general TFs or RNAPII (extended discussion in Supplementary Note 5). The 31 variants generated from *HHX2* histone promoter deletions (Fig. 2c, d) spanned a >15-fold range in cumulative expression levels and up to 39-fold expression ratio between sides.

### Creation of inducible sBDPs by MDP bidirectionalization

We next sought to introduce inducibility to this library of promoters with varied expression strength and ratios by incorporating design elements from the inducible MUT pathway. As mentioned, MUT promoters such as *P*_*DAS1-DAS2*_ (Supplementary Fig. 2) showed promise because of their expression capacity (Fig. 1c) but are cumbersome to work with due to size (2488 bp). To solve this, we aimed to generate shorter and more flexible inducible BDPs by bidirectionalizing MDPs, fusing a second core promoter in reverse orientation to an MDP (Fig. 3a). As core promoters in eukaryotes typically provide little expression on their own, strong expression generally requires upstream activating sequences, which are also referred to as enhancers or *cis*-regulatory modules (CRMs)^43^, with the CRM terminology including repressor-binding sites (Fig. 3a illustration). Here the previously identified short core promoter/5′ UTRs of the histone promoters held utility (Fig. 2b). We hypothesized that adding a short, non-regulated core promoter in reverse orientation upstream of an MDP could duplicate the expression and regulation of the native orientation^24,25^.

Accordingly, we fused 6 histone core promoters to 12 monodirectional *P. pastoris* promoters, partly varying the lengths of the core promoters and the MDPs (Fig. 3a). Two thirds of the 30 constructs were successfully bidirectionalized, showing detectable expression from the second core promoter. In the case of three promoters (*P*_*AOX1*_, *P*_*FLD1*_, and *P*_*DAS2*_), bidirectionalized expression >50% of the native monodirectional side was reached. The construct *Pcore*_*HTA1-81*_+*P*_*DAS2-699*_ even outperformed strong MDPs. Different core promoter lengths only moderately affected expression, while MDP length had a drastic effect (e.g., *Pcore*_*HTA1-81*_+*P*_*DAS2-699*_ vs. *Pcore*_*HTA1-81*_+*P*_*DAS2-1000*_: very high vs. no bidirectionalized expression). This was perhaps surprising in light of milestone bidirectionalization studies in higher eukaryotes^24,25^ where testing only a few promoters in a single length led to suitable BDPs. These dissimilarities may be explained by a different function/distance relationship between CRMs from yeast and higher eukaryotes.

### Creation of fusion sBDPs with varied regulation

**Fig. 4:**
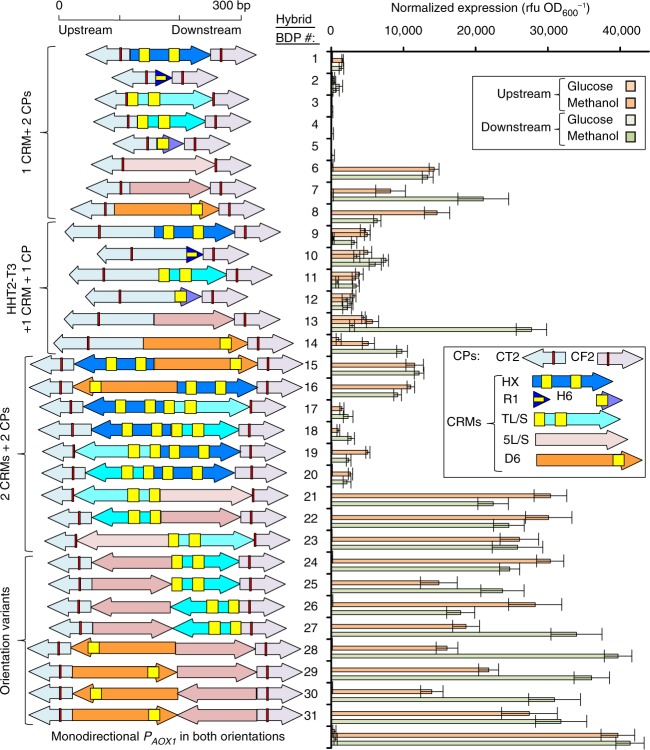
Modularly designed and exceptionally short bidirectional hybrid promoters (179–457 bp) achieve the highest expression strength per bp of promoter length matching the strong monodirectional *AOX1* promoter (940 bp length). The bidirectional hybrid promoters were assembled from histone core promoters (Fig. 2b) and CRMs of methanol-regulated promoters (Supplementary Fig. 2, Supplementary Fig. 4). The detailed color code for the regulatory elements/abbreviations used is provided in Supplementary Fig. 4; a list of the exact designs of shBDP1–31 is provided in Supplementary Data 2. Yellow boxes indicate experimentally confirmed Mxr1p (methanol master regulator) binding sites in *P*_*AOX1*_ and *P*_*DAS2*_ (Supplementary Fig. 2, Supplementary Fig. 4); red boxes: TATA boxes. Additional bidirectional variants, controls, and extended discussion are provided in Supplementary Fig. 4 and Supplementary Note 5. *P*_*AOX1*_ is a reference of a monodirectional, strong, methanol-inducible promoter. *P*_*AOX1*_ was cloned in forward and reverse orientation in the bidirectional reporter vector, therefore the values shown are derived from separate constructs and not from bidirectional activity. CP core promoter, CRM *cis*-regulatory module. “HHT2-T3” is the truncated side of a bidirectional histone promoter (see Fig. 2c, d) used to generate hybrid promoters with growth-associated expression from one side. Strains were grown on glucose media for 60 h subsequently induced with methanol for 48 h. Mean values and standard deviations of normalized (using the normalization factor calculated in Supplementary Fig. 1) reporter protein fluorescence measurements of biological quadruplicates grown on the respective carbon sources are shown (see Supplementary Data 2 for the exact values). All elements used (except for the non-regulated core promoters and constitutive *HHT2-T3*) are methanol inducible

**Fig. 5:**
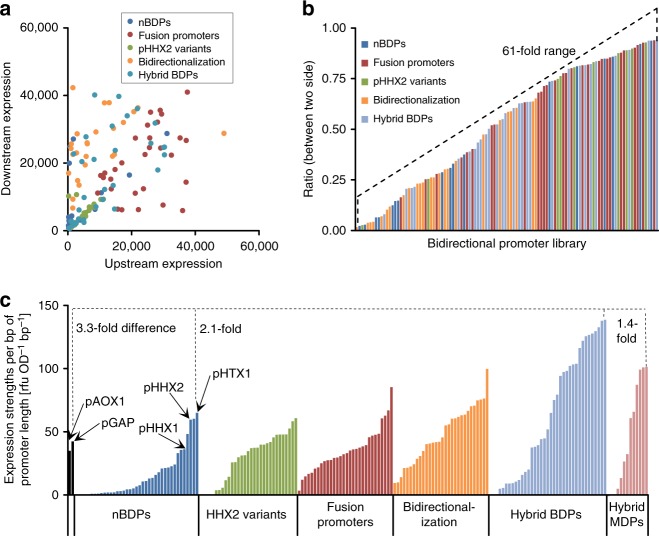
The library of 168 BDPs provides different absolute expression strengths, ratios, and regulatory profiles with synthetic BDPs (sBDPs) considerably surpassing the expression strength per bp of promoter length of natural BDPs (nBDPs). **a** The library of BDPs covers the whole expression space. Normalized upstream and downstream reporter fluorescence is shown (rfu OD^−1^ as in Fig. 1 to Fig. 4; under optimal growth conditions, by the default orientation in which the BDPs were cloned in the reporter vector). **b** The library of BDPs offers different ratios between the two sides of the promoters, ranging from equal expression to a 61-fold difference. The ratios were calculated from the normalized reporter protein fluorescences (under optimal growth conditions) by dividing the lower value by the higher value. Different growth conditions of the strains with differently regulated promoters even extend the ratios achievable. Only promoters clearly exceeding the background signal of the measurements (>500 rfu for eGfp, >100 rfu for dTom) were included in the calculations. **c** Expression strengths per bp of promoter length of sBDPs exceed nBDPs up to 2.1-fold and nMDPs up to 3.3-fold. “Expression strength per bp of promoter length” is a term introduced in this study to illustrate the relationship between promoter length and promoter strength. The expression strengths per bp of promoter length were calculated by adding up the normalized reporter protein fluorescence measurements of both sides (under optimal growth conditions) and dividing the sum by the length of the promoter (bp). Hence the expression strengths per bp of promoter length are relative terms and will change with different fluorescence reporter proteins used and even with different fluorospectrometers for detection. The monodirectional *AOX1* and *GAP* promoters are included as references for state-of-the-art nMDPs. Fold differences between the most efficient hybrid promoters and the most efficient nBDPs, hybrid MDPs, and the monodirectional reference promoters are shown

All BDPs to this point possessed the same regulation on both sides. Having varied regulation can allow for expression cascades, which can be beneficial when it is necessary to express one gene before another, such as a chaperone before its protein folding target. We generated fusions of constitutive, derepressed, and inducible MDPs^30^, creating 30 fusion sBDPs with distinct regulation on each side (Fig. 3b, c; Supplementary Note 4, Supplementary Table 1). These fusions generally maintained each side’s original regulation and individual expression levels, allowing for the creation of variably regulated BDPs with a range of expression ratios between sides (0.16–0.96). A subset of the fusion promoters (Fig. 3c) consisted of combinations of *DAS1* and *DAS2* deletion variants (Supplementary Fig. 2, Supplementary Note 3) demonstrating that separately engineered MDPs maintain their individual expression levels and can be rationally combined to generate BDPs with desired expression ratios. Some fusion variants showed synergistic effects, such as the 1.8-fold increase in the expression for a *GAP-DAS2* fusion promoter. Others showed antagonistic effects, such as the 40% repression of a *HTA1-TAL2* fusion promoter, suggesting a transcriptional “spillover” between promoters (Supplementary Fig. 3). These findings contrast previous MDP fusion studies in *S. cerevisiae*^23,44–47^, potentially due to the greater number of promoters and combinations tested here. It is known that binding of insulator proteins can decouple regulation of BDPs per side in *S. cerevisiae*^18^, and thus the properties of fusion promoters are difficult to predict. These synergistic effects, though, can be harnessed to design shorter, more efficient promoters and so we expanded this principle to the design of hybrid promoters (Figs. 4, 5c), ultimately finding it successful.

### Creation of short hybrid sBDPs

Through the creation of this sBDP library, it became clear that we had little ability to predict function based on promoter length and core promoter properties alone. To help improve our understanding, we assembled short defined CRMs (30–175 bp, Supplementary Fig. 2, Supplementary Fig. 4, Supplementary Note 5) with histone core promoters (Fig. 2b) into compact bidirectional hybrid^48^ promoters (Fig. 4). The CRMs were selected from methanol-regulated promoters based on literature data available on *P*_*AOX1*_ (^31^, Supplementary Fig. 2) and deletion studies on *P*_*DAS1*_ and *P*_*DAS2*_ (Supplementary Fig. 2, Supplementary Note 3). Each CRM was characterized with a single core promoter (Supplementary Fig. 4b), two core promoters, and combinations of CRMs in different positions and orientations (Fig. 4). To create combinations of regulatory profiles, we fused a truncated histone promoter variant (*P*_*HHT2-T3*_, Fig. 2c, d) to a single CRM and one core promoter.

Inducible synthetic hybrid BDPs matched expression from the monodirectional *AOX1* reference promoter (bottom of Fig. 4). However, the generated sBDPs were considerably shorter (179–457 bp) than *P*_*AOX1*_ (940 bp). To illustrate this length advantage, we characterized their expression strength per bp of promoter length, which we define as normalized fluorescence per bp in this study. As the expression output depends on the reporter protein, these expression strengths per bp of promoter length are dependent upon the fluorescence reporter proteins and even spectrometers used. Hybrid BDPs showed up to 3.3-fold higher expression strengths per bp of promoter length than typically used nMDPs and were 2.1-fold more efficient than the most efficient nBDP (Fig. 5c). In addition, synthetic MDP controls were up to 2.4-fold more efficient than nMDPs (Supplementary Fig. 4). The length of the core promoters and the orientation of the CRMs only marginally affected the expression of the hybrid BDPs. Orientation independency in yeast CRMs has long been known^38^, and our results demonstrate that this property can also be harnessed to generate strong sBDPs.

In summary, the modular design strategies outlined (Figs. 2b–d, 3, and 4) produced a versatile library of 168 BDPs offering (1) different regulatory profiles, (2) providing a 79-fold range of cumulative expression, and (3) up to 61-fold expression ratio between sides, meeting the intended design requirements for our library (Fig. 5a, b).

### BDPs facilitate dual gene co-expression optimization

**Fig. 6:**
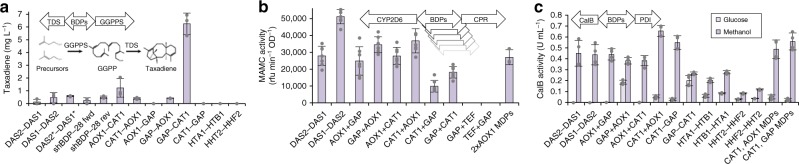
Applying the library of BDPs helps to find the optimal expression condition for dual gene co-expression. For each of the three pair of genes tested, a different BDP performed best and the activity/yields for the same set of genes spanned a 5.2–50-fold range. **a** Highest taxadiene yields were achieved using a *P*_*GAP-CAT1*_ fusion promoter for GGPPS and TDS co-expression. The designs based on different BDPs span a 50-fold range in yields. DAS2*-DAS1* denotes the improved promoter variant *DAS2-d8-DAS1-d2d5* (Fig. 3c, Supplementary Fig. 2). Constitutive expression of the GGPPS gene was detrimental. Yields determined by GC-MS from shake flask cultivations (biological triplicates, mean value, and standard deviation shown) with a dodecane overlay. **b** Highest activity for the co-expression of human CYP2D6 and its associated CPR was achieved using the natural *P*_*DAS1-DAS2*_ promoter in reverse orientation. The designs based on different BDPs span a 5.2-fold activity range. “2×* AOX1* MDPs” indicates a control strain expressing the two genes using two monodirectional *AOX1* promoters. The strains were pre-grown for 60 h on glucose and induced with methanol for 72 h. Activity of biological seven-fold replicates (mean value and standard deviation shown) was measured by a whole-cell bioconversion assay using 7-methoxy-4-(aminomethyl)-coumarin (MAMC) as substrate. **c** Bidirectional fusion promoters of *P*_*CAT1*_ to *P*_*AOX1*_ or *P*_*GAP*_ give the highest volumetric activities in the co-expression of secreted CalB and the chaperone PDI. The designs based on different BDPs span a 22-fold activity range. “*CAT1, AOX1*, MDPs” and “*CAT1, GAP* and MDPs” are control strains mimicking the best bidirectional designs with MDPs. Activities in the supernatant of biological quadruplicates (mean value and standard deviation shown) were measured after growth for 60 h on glucose and methanol induction for 72 h using a p-nitrophenyl butyrate (pNPB) assay

After developing a cloning strategy to insert the library of BDPs into a cloning junction between genes of interest (Supplementary Fig. 5, Supplementary Note 6), we next aimed to demonstrate the utility of our BDP library for optimizing multi-gene co-expression. First, we optimized dual gene co-expression for production of taxadiene (Fig. 6a), the first committed precursor of the potent anticancer drug Taxol (paclitaxel), which requires expression of geranylgeranyl diphosphate synthase (GGPPS) and taxadiene synthase^3^. Second, we evaluated co-expression of a human cytochrome P450s (CYP2D6) and its electron-donating NADPH-dependent reductase partner (CPR) using a subset of strong, differently regulated BDPs from the library (Fig. 6b). Third, we evaluated the effect of the chaperone protein-disulfide-isomerase (PDI) on secretion of the disulfide-bond-rich biocatalyst *Candida antarctica* lipase B (CalB, Fig. 6c).

Our results showed that constitutive expression worked only for CalB. Constitutive expression of endoplasmic reticulum-localized CYP2D6/CPR may exert too much stress on the cells, leading possibly stress responses and degradation driving its activity below the limit of detection. For taxadiene production, we noticed an approximately 100-fold decrease in transformation rates when the GGPPS gene was under control of a constitutive promoter, with the few candidate colonies showing no detectable taxadiene production. For the three gene pairs tested (Fig. 6a–c), there was a 5.2–50-fold difference in activity/yields of the best and worst performing promoter choice. Most strikingly, for taxadiene production, the worst strain produced only 0.1 mg L^−1^, whereas the best strain (bearing a *P*_*GAP+CAT1*_ fusion promoter) reached 6.2 mg L^−1^, in range with engineered *S. cerevisiae* strains (8.7 ± 0.85 mg L^−1^)^49^.

We presume that the high yield of this strain is mostly attributable to the use of *P*_*CAT1*_ to drive expression of the GGPPS gene, as also the second-best design (*P*_*AOX1-CAT1*_) had GGPPS under the control of the same promoter. *P*_*CAT1*_ is a derepressed promoter, meaning expression starts once the glucose in the media is depleted and is further strongly induced by methanol^30^. So, in the best taxadiene-producing strains, the GGPPS gene was at first repressed, partially activated in the derepressed phase, and then fully activated on methanol. This demonstrates, in addition to the importance of the ratio and strength of the promoters, that the regulatory profile is critical and can be easily optimized using this versatile library of sBDPs. Tailoring cultivation conditions toward each side of a BDP may further help to optimize yields^50^. Worth noting, each application had a different best promoter (GGPPS+TDS: *P*_*GAP+CAT1*_, CYP2D6+CPR: *P*_*DAS1-DAS2*_, CalB+PDI: *P*_*CAT1-AOX1*_) and the obtained titers/activities did not necessarily correlate with reporter protein fluorescence measured previously for these BDPs (Supplementary Fig. 6), highlighting gene-pair-specific effects and the importance of screening a diverse library (Fig. 6a–c). Once optimized expression profiles were known, they could be quickly recreated with MDPs (Fig. 6b, c), demonstrating that even if MDPs should be used for the final design, BDPs can be used to identify optimal expression profiles with faster and simplified cloning techniques, as previously discussed (Supplementary Fig. 5, Supplementary Note 6).

### BDPs alongside BDTs simplify multi-gene pathway fine-tuning

**Fig. 7:**
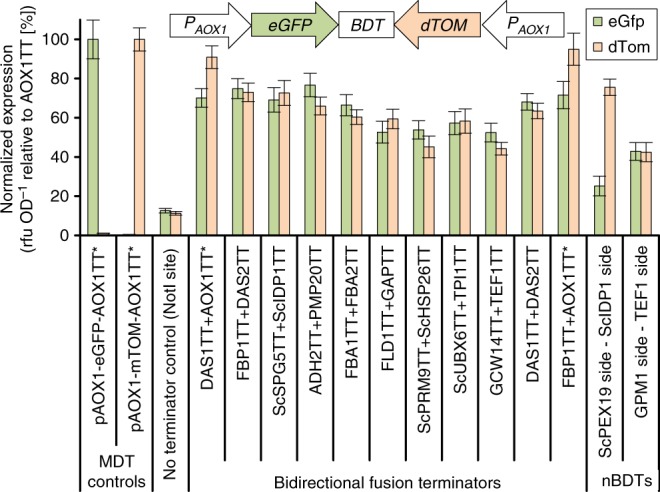
Bidirectional transcription terminators (BDTs) required for the assembly of bidirectional multi-gene co-expression relieve expression loss associated with transcriptional collision. A reporter construct for testing bidirectional transcription termination was assembled by cloning the genes coding for eGfp and dTom in convergent orientation (small inlet). Two *AOX1* promoters were used to drive equal expression of the reporter genes. Monodirectional terminators (MDTs) were combined into bidirectional fusion terminators and two putative natural BDTs (nBDTs) were tested. A negative control lacking termination sequences and bearing solely a *Not*I restriction site was included. Additional control constructs contain only a single *AOX1* promoter, a single FP, and the AOX1* terminator. AOX1TT* denotes the *AOX1* terminator sequence used by Vogl et al.^30^. Some BDTs acted also as autonomously replicating sequences (Supplementary Fig. 7). Mean values and standard deviations of fluorescence measurements after pre-growth on glucose followed by methanol induction of biological quadruplicates are shown

Finally, we wanted to assemble a pathway with greater than two components. In doing so, we quickly found that, with increasing numbers of genes, inclusion of bidirectional terminators (BDTs) was necessary. Lack of BDTs in this context results in transcriptional collision as polymerases transcribing opposite DNA strands in convergent orientation stall upon collision^51–53^. We combined selected MDTs, including heterologous *S. cerevisiae* terminators shown to be active in *P. pastoris*^30^, into 11 bidirectional fusion terminators by linking them in convergent orientation (Fig. 7). Additionally, natural BDTs (nBDTs) can be used as the *P. pastoris* genome harbors 1461 putative BDTs from genes in tail-to-tail orientation (Fig. 1b). We included two such short nBDTs from both *P. pastoris* and *S. cerevisiae*.

The BDTs were cloned, maintaining the natural transition between stop codon and terminator without any additional restriction sites, into a reporter vector containing two FPs in convergent orientation (Fig. 7). Complete lack of a termination signal in this context, created by leaving only an 8 bp *Not*I restriction between the reporter genes, resulted in an ~8-fold reduced reporter gene fluorescence, suggesting that transcriptional collision occurs to similar extents in *P. pastoris* as reported in *S. cerevisiae*^51–53^. Providing either fusion terminators or nBDTs showed clear improvements compared to the no terminator control, restoring 50–90% of reporter protein fluorescence. As in previous work on *P. pastoris* MDTs^30^, we also noticed that some BDTs functioned as autonomous replicating sequences (ARS) (Supplementary Fig. 7), which may lead to increased background growth and strain instability for episomally replicating sequences. We therefore recommend screening new BDTs for ARS function, as fusion terminators behaved in part differently from the originating MDTs (Supplementary Fig. 7).

**Fig. 8:**
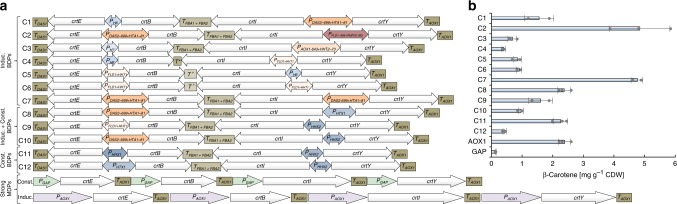
The library of BDPs and BDTs facilitates the assembly and transcriptional fine-tuning of multi-gene pathways demonstrated with the four gene (*crtE*, *crtB*, *crtI*, *crtY*) model pathway of β-carotene biosynthesis. **a** Using BDPs and BDTs for pathway assembly reduces construct length and the number of parts required. Twelve bidirectional constructs were assembled by combining inducible or constitutive BDPs and combinations thereof (Induc.+const.) with a BDT and two MDTs. See Supplementary Fig. 5d for assembly strategy and supporting file Supplementary Data 2 (sheet “Carotenoid pathway constructs”) contains detailed information on the BDPs/BDTs used. For the BDPs, a coloring scheme similar to Fig. 5 was used. T* natural bidirectional terminator between the *S. cerevisiae IDP1* and *PEX19* genes, *T*^*+*^ natural bidirectional terminator between the *P. pastoris TEF1* and *GDM1* genes. The bidirectionalized *P*_*FLD1-366+HHT1-91*_ was used. **b** β-Carotene titers obtained with strains based on the bidirectional constructs shown in **a** span a 12-fold range matching or surpassing conventional *P*_*AOX1*_- and *P*_*GAP*_-based designs. Mean values and standard deviations of biological triplicate cultivations in shake flasks are shown (HPLC measurements)

With these BDTs available, we tested combinations of BDPs (constitutive, inducible, expression ratios) to optimize expression of the four-gene carotenoid pathway for β-carotene synthesis (Fig. 8a). Monodirectional cassettes using *P*_*AOX1*_ (inducible) and *P*_*GAP*_ (constitutive) were included as reference. The bidirectional constructs showed a 12.1-fold range in β-carotene yields, with the highest β-carotene yield coming from the methanol-inducible bidirectional designs (C2/C7, Fig. 8b). This construct surpassed the monodirectional *P*_*AOX1*_ design two-fold and matched the best MDP-based inducible construct previously reported in *P. pastoris* (5.2 ± 0.26 mg g^−1^ cell dry weight)^30^. Regarding constitutive/growth-associated expression of the pathway, the best bidirectional design based on histone promoters (C11) yielded 14.9-fold higher β-carotene titers than the monodirectional standard *P*_*GAP*_ design. This improvement may be explained by the regulation of the promoters used. *P*_*GAP*_ is constitutively expressed and constitutive expression of the β-carotene pathway from this promoter may present too great a metabolic burden. Core histone genes, in contrast, are cell cycle regulated and typically only activated in the late G1 phase to provide sufficient histones for the newly replicated DNA in the S phase^7^. It appears plausible that cell cycle-associated expression from histone promoters exerted less metabolic burden than entirely constitutive expression from *P*_*GAP*_, leading to their improved function.

## Discussion

Constructing efficiently expressed and well-balanced pathways is paramount for harnessing biology to its full industrial potential. Here, using the natural histone BDPs of *P. pastoris* as template, we combined multiple engineering strategies, including truncation and MDP bidirectionalization, to develop a library of sBDPs with a broad range of expression levels and ratios and with different regulation profiles. We found that this library not only covers diverse expression profiles but also is highly efficient in terms of the expression output. Even more, we demonstrated its utility for multi-gene pathway optimization, highlighted by simple optimization experiments for taxadiene and β-carotene production. By screening of our large 168 member library, we identified a subset of highly useful BDPs and compiled a minimal set of 12 BDPs (6 BDPs to be tested in both orientations, Table 1 and Supplementary Data 3 for annotated sequence files). These promoters have regulatory diversity, different strengths, and ratios. In addition, this subset offers extended diversity if cultivated with different carbon sources (glucose/glycerol, methanol). Screening with this initial set provides a foundation for subsequent fine-tuning.

**Table 1:** Minimal set of diverse BDPs covering broad regulatory profiles for co-expression optimization

BDP	Regulation	Strength	Ratio
*P*_*HTX1 (HTA1-HTB1)*_	Constitutive on both sides (cell cycle/growth associated in *S. cerevisia*e^7^)	Strong on both sides	~1:1
*P*_*DAS2-699-pCoreHTA1-81*_	Methanol inducible (tightly glucose/glycerol repressed) on both sides	Strong on both sides	~1:2
*P*_*CAT1-FDH1*_	Derepressed/methanol inducible on both sides	Weak/moderate under derepression, strong on methanol	~1:1
*P*_*AOX1-CAT1*_	Methanol inducible (tightly glucose/glycerol repressed)–derepressed, methanol inducible	Strong on both sides on methanol, *P*_*CAT1*_ moderate under derepressed conditions	Derepressed: ~0:1 induced: ~1:1
*P*_*AOX1-GAP*_	Methanol inducible (tightly glucose/glycerol repressed)–constitutive	Strong on both sides (*P*_*GAP*_ side moderate on methanol)	Glucose/derepressed: ~0:1 induced: ~1:0.5
*P*_*GAP-CAT1*_	Constitutive–derepressed, methanol inducible	*P*_*GAP*_ side strong constitutive (moderate on methanol), *P*_*CAT1*_ side moderate derepressed, strong on methanol	Derepressed: ~1:0.25 induced: ~1:2

In dual gene expression applications, each BDP should be tested in forward and reverse orientation. Reporter protein fluorescences of the respective promoters are shown in Figs. 2a and 3a, b. Annotated GenBank files for these promoters are provided as Supplementary Data 3. For multi-gene co-expression furthermore, the three histone promoters *HTX1*, *HHX1*, and *HHX2* and additional methanol-inducible promoters (e.g., shBDP23 [Fig. 4], *FLD1*+*PMP20*, *FBA2*+*TAL2* [Fig. 3b]) are useful.

Generating similar BDP libraries in other organisms will require species-specific engineering, especially for obtaining inducible promoters. Methanol-inducible promoters are rather unique to *P. pastors* and other methylotrophic yeasts^31^, whereas other systems will require species-specific promoters such as galactose-regulated promoters in *S. cerevisiae*^38^. In higher eukaryotes, where carbon-source-regulated promoters are scarce, inducible BDPs based on synthetic TFSs^26^ could be generated relying on strategies developed for MDPs^54,55^.

However, as this library strategy relies on parts from the highly conserved histone BDP architecture, with homologs in *S. cerevisiae*, *Schizosaccharomyces pombe*, and even Chinese Hamster Ovary cells, we have reason to believe that the promoter engineering and cloning strategies outlined in this work will be generalizable to other eukaryotes. Hence, the use of similar BDP libraries is likely to expand to many hosts and allow for efficient and rapid pathway optimization, expanding the possibilities of synthetic biology and metabolic engineering.

## Methods

### Promoter reporter vectors

The *P. pastoris* CBS7435 wild-type strain was used for most experiments. The control strain expressing the four genes of the carotenoid pathway under control of four *AOX1* promoters was available from Geier et al.^56^. This strain contains the identically codon optimized genes of the carotenoid pathway used in this study each under control of the *AOX1* promoter and terminator. For CalB expression, mut^S^ strains^57^ were used, as higher productivity on methanol has been reported^58^.

Details on the promoters and terminators used in this study (including primers for amplification) and the list of primers for generating the reporter vectors and applications (pathway assembly, etc.) are provided in Supplementary Data 2. A subset of annotated sequences of a minimal set of BDPs covering broad regulatory profiles for dual gene expression optimization is provided in Supplementary Data 3 file in GenBank format (and summarized in Table 1 in the main manuscript).

For basic characterizations, a pPpT4_S^57^ based expression vector (Zeocin selection marker) bearing a single *eGFP* reporter gene reported by Vogl et al.^30^ was used (pPpT4mutZeoMlyI-intARG4-eGFP-BmrIstuffer^30^). This vector contains integration sequences near the *ARG4* locus and was linearized with *Swa*I to target insertion near the *ARG4* locus, as had been well established for promoter characterizations in *P. pastoris*^30,39,41^. Also the following vectors described below were based on this vector backbone. With the single reporter vector, BDPs had to be cloned twice, once in forward and once in reverse orientation. The *P. pastoris* nBDPs were initially characterized by these means. To reduce the cloning effort and allow simultaneous detection of both sides, we designed a bidirectional screening vector. Based on the single reporter vector, we inserted a second reporter gene (a red FP variant termed dTomato^59^) between the targeting sequence and the stuffer fragment of pPpT4mutZeoMlyI-intARG4-eGFP-BmrIstuffer. The vector was assembled by digesting the single reporter vector with *Asc*I and *Avr*II. Subsequently, the dTomato fused to a *P. pastoris* transcription terminator sequence was PCR amplified from a *P. pastoris* cloning vector using primers TomatoAscIBmrIFWD and AOXTTSbfIAvrIIREV1. To add an additional *Sbf*I restriction site, the obtained PCR fragment was used as template for a second PCR using primers TomatoAscIBmrIFWD and AOXTTSbfIAvrIIREV2. The newly inserted part was confirmed by Sanger sequencing. This vector was named pPpT4mutZeoMlyI-intArg4-bidi-dTOM-eGFP-BmrIstuffer. Subsequently, we cloned several natural BDPs and semisynthetic fusion promoters into this vector (primers provided in Supplementary Data 2). The promoters were either inserted in random orientation by TA cloning^60^ or directional by Gibson assembly^61^.

### BDTs reporter vector and cloning of BDTs

The reporter vector for BDTs contained two convergent expression cassettes each consisting of an *AOX1* promoter and an *eGFP* or *dTOM* reporter gene, respectively (see illustration in Fig. 7). The 3′ ends of the reporter genes are separated by a stuffer fragment that can be replaced with a BDT. The reporter vector was assembled by digesting a monodirectional control vector containing an *AOX1* promoter upstream of *eGFP* (pPpT4mutZeoMlyI-intArg4-EGFP-AOX1BglII)^30^ with *Not*I and *Bam*HI. Subsequently, the *AOX1* promoter fused to the dTomato gene was amplified using primers stuffer-dTom-Gib and pILV5-pAOX1-Gib (from the *P*_*AOX1*_-dTOM side of a bidirectional vector used in this study). The stuffer fragment was amplified using primers eGFP-stuffer-Gib and dTom-stuffer-Gib from the vector pPpT4mutZeoMlyI-intARG4-eGFP-BmrIstuffer as template^30^. The primers replaced the *Bmr*I sites with *Not*I sites, as the *P*_*AOX1*_ contains a *Bmr*I site and removal of the stuffer fragment using *Bmr*I would also impair the rest of the backbone. The vector backbone and the two PCR products were combined in a Gibson assembly reaction and verified by Sanger sequencing.

For cloning of the BDTs, the reporter vector was digested with *Not*I and the backbone was gel purified. The BDTs were amplified with overhangs to the 3′ ends of the reporter genes (using the primers listed in Supplementary Data 2) and cloned by Gibson assembly into the vector. Note that for the bidirectional fusion terminators each monodirectional terminator was amplified separately with an overhang to the other one. In this case, the terminators were fused in the Gibson assembly reaction by adding three fragments (vector backbone and two PCRs of the two monodirectional terminators). The inserted terminators were sequenced using primers seqEGFP-520..543-fwd and seqTomato-517..540-fwd.

### Cloning vector for dual or multi-gene co-expression

The aforementioned bidirectional reporter pPpT4mutZeoMlyI-intArg4-bidi-dTOM-eGFP-BmrIstuffer vector can also be used as entry vector for the co-expression of any gene pair. Therefore, a cassette consisting of the two genes to be co-expressed with a stuffer fragment between them is assembled by olePCR, digested with *Not*I, and cloned in the *Not*I-digested bidirectional double reporter vector backbone (general concept outlined in Supplementary Fig. 5 and Supplementary Note 6). Alternatively, also Gibson assembly can be used. This vector contains *AOX1* terminators on both sides, hence directional cloning (even by Gibson assembly) is not possible.

To facilitate the generation of entry vectors for oriented cloning of two or more genes, we generated a cloning vector, which provides two different MDTs (*T*_*AOX1*_ and *T*_*DAS1*_) in opposite orientation separated by a *Not*I restriction site. If two genes (dual gene co-expression) or a multiple genes (multi-gene co-expression) should be co-expressed, this vector can be used for insertion. We prepared two different cloning vectors: pPpT4_S-DAS1TT-NotI-AOX1TT and pPpT4mutZeoMlyI-intArg4-DAS1TT-NotI-AOX1TT. The former is based on the pPpT4_S vector reported by Näätsaari et al.^57^: Following *Not*I and *Swa*I digestion and purification of the backbone, a PCR product of the *T*_*DAS1*_ bearing overhangs to the vector (primers: P_AOX1_Syn-SwaI-DAS1TT-3prime-Gib and AOX1TT-5prime-NotI-DAS1TT-5prime-Gib) was cloned by Gibson assembly and subsequently confirmed by sequencing. The latter vector contained in addition a sequence to target specific genomic integration (intArg4) and a mutated *Mly*I site in the Zeocin resistance gene (silent mutation^60^). This vector was generated by digesting the aforementioned pPpT4mutZeoMlyI-intArg4-bidi-dTOM-eGFP-BmrIstuffer with *Sbf*I and *Not*I and inserting a PCR product containing the respective overhangs (primers: intARG4-SbfI-DAS1TT-3prime-Gib and AOX1TT-5prime-NotI-DAS1TT-5prime-Gib) by Gibson assembly. Again, the vector was confirmed by sequencing.

### Cloning different BDPs for dual gene co-expression

Our screening strategy for the optimal BDP for a certain gene pair (Supplementary Fig. 5a–c) requires an entry vector containing the two co-expressed genes in which the promoter can be easily exchanged. A stuffer fragment in this entry vector is subsequently cut out by *Bmr*I digestion and replaced with BDPs. Note that the genes to be co-expressed must not contain *Bmr*I sites.

The vector for taxadiene co-expression was generated by ordering *P. pastoris*-codon-optimized GGPPS and TDS genes. The genes were ordered as synthetic double-stranded fragments (gBlocks by Integrated DNA Technologies) with overhangs for Gibson assembly (gBlock-GGPPS_optTV-AOX1TT-Gib, gBlock-TDS_optTV-Part1 and gBlock-TDS_optTV-Part2-DAS1TT-Gib). A stuffer fragment with complementary overhangs was amplified using primers TDS-BmrI-stuffer-Gib and GGPPS-BmrI-stuffer-Gib. The four fragments were mixed in equimolar ratios with the *Not*I-digested pPpT4mutZeoMlyI-intArg4-DAS1TT-NotI-AOX1TT backbone and joined by Gibson assembly. The entire inserted cassette was sequenced. This vector was named pPpT4mutZeoMlyI-intArg4-DAS1TT-AOX1TT-TDS_optTV-GGPPS_optTV-BmrIstuffer.

After removal of the stuffer fragment by *Bmr*I digestion and gel purification, a set of the respective differently regulated promoters was amplified, cloned into the entry vectors, and verified by sequencing. See Supplementary Data 2 for the exact primers and overhangs used.

In a similar way, entry vectors for CYP2D6/CPR co-expression and CalB/PDI co-expression were generated. The coding sequences were available from previous studies (CYP2D6/CPR^62^, CalB^30^, PDI^63^). See also Supplementary Data 2 for the exact primers and overhangs used. For CYP2D6/CPR, the monodirectional control strain (Fig. 6b) containing a single copy of a vector with each gene under control of an *AOX1* promoter was available from previous work and was generated by cloning each gene into pPpT4 and pKan vectors^57^ via *Eco*RI and *Not*I sites and after the transformation a transformant with a single copy of each plasmid was selected. The monodirectional CalB/PDI control constructs shown in Fig. 6c were generated by cloning the respective promoters into the same pPpT4 vector (using the standard *AOX1TT*).

### Assembly of multi-gene cassettes for the carotenoid pathway

Constructs with different BDPs and terminators were designed for the expression of the carotenoid pathway (four genes *Crt*E, *Crt*B, *Crt*I, and *Crt*Y) in *P. pastoris* and are shown in Fig. 8a. The exact promoters, terminators, and primers for amplification are provided in Supplementary Data 2. The BDPs and terminators were selected based on their length, function, and sequence characteristics. Combinations of promoters of different strengths and regulations were tested (inducible, constitutive, constitutive+inducible). Also a construct with switched positions of the BDPs was created to evaluate the effect of positioning the promoter between the first two or the last two genes.

The vector backbone pPpT4_S-DAS1TT-NotI-AOX1TT containing two monodirectional terminators *T*_*AOX1*_ and *T*_*DAS1*_ in opposite orientation with a *Not*I restriction site in between was used for insertion of the pathway. The genes, BDPs, and terminators were amplified by PCR, using the primers listed in Supplementary Data 2. The primers for the amplification of the promoter and terminator sequences contained overhangs to the carotenoid genes. The fragments were linked by Gibson assembly. In order to increase the efficiency of the Gibson assembly, the number of fragments, which have to be combined, was reduced by a preassembling step via overlap extension PCR. After combining the carotenoid genes with the adjacent promoter or terminator, the preassembled fragments were connected by Gibson assembly and used to transform *E. coli*. Plasmid DNA was isolated from transformants and the sequences were verified by sequencing.

### Cultivation conditions and screening procedures

The *P. pastoris* cultivations were performed using a high-throughput small-scale 96-deep-well-plate (DWP) cultivation protocol^64^. Briefly, wells containing 250 µL BMD1 (buffered minimal dextrose medium, as reported^64^) were inoculated with a single colony from transformation plates and grown for 60 h on glucose. For induction, a final methanol concentration of 0.5% (v/v) was used. Cells were induced with 250 µL of buffered media with 1% methanol (BMM2) after 60 h of growth on glucose. After 12 h, 24 h up to 48 h, 50 µL of BMM10 (with 5% methanol) was added for further induction.

*P. pastoris* cells were transformed with molar equivalents to 1 µg of the empty pPpT4_S vector *Swa*I linearized plasmids^65^ (1 µg of the empty pPpT4_S vector was found to yield predominantly single copy integration^40,66^. Some of the vectors used in this study are, however, considerably large than the empty pPpT4_S vector [e.g., the carotenoid pathway constructs], hence in these cases we increased the DNA amounts to have an equivalent number of vector molecules compared to the empty pPpT4_S vector). The following antibiotic concentrations were used: *E. coli*: LB-medium containing 25 μg mL^−1^ Zeocin; *P. pastoris*: 100 μg mL^−1^ Zeocin. The screening and rescreening procedures to compare single *P. pastoris* strains have previously been reported^30,40^. In brief, for each construct 42 transformants (approximately half a DWP) were screened to avoid clonal variation observed in *P. pastoris*^66–68^. Three representative clones from the middle of the obtained expression landscape (to avoid outliers of multi-copy integration or reduced expression because of deletions^66^ or undesired integration events^67,68^) were streaked for single colonies and rescreened in biological triplicates. Finally, one representative clone was selected and a final screening of all the variants together was performed.

### Fluorescence reporter measurements and assays

The fluorescence reporter and OD_600_ measurements were performed using 96-well microtiter plates (Nunc MicroWell 96-well optical-bottom plates with polymer base, black; Thermo Fisher Scientific) and a Synergy MX plate reader (Biotek, Winooski, VT, USA). Enhanced green fluorescent protein (eGfp) measurements were performed at excitation/emission wavelengths of 488/507 nm^30,40^. dTomato was measured at excitation/emission wavelengths of 554/581 nm^59^. Cell suspensions were diluted to remain within the linear range of the plate reader (1:20-fold, i.e., 10 µl of cell suspension were added to 190 µl of ddH_2_O and mixed by shaking in the plate reader). For eGfp and dTom fluorescence and OD_600_ measurements, the background signals of diluted medium were removed and rfu normalized per OD_600_ to correct for dilution errors and different amounts of cell material.

CalB activities in the supernatants were determined using an esterase activity assay with p-nitrophenyl butyrate (pNPB) as substrate^30,58^. After centrifugation of the cell suspensions, 20 μl of undiluted cultivation supernatant were mixed with 180 μl of assay solution (buffer: 300 mM Tris-HCl, pH 7.4, 1% dimethyl sulfoxide (DMSO) and 4 mM pNPB). The change in absorbance per time (over 5 min) was measured using the aforementioned Synergy MX plate reader at 405 nm.

CYP2D6 activity measurements were performed using 7-methoxy-4-(amino-methyl)-coumarin (MAMC) as substrate^69^. Cell suspensions from cultivations in 96 DWPs were centrifuged and the supernatants were discarded. Cells were gently resuspended in 200 µl 100 mM potassium phosphate buffer, pH 7.4. This washing step was repeated once and 95 µl of the resuspended cells were transferred to the aforementioned Nunc microtiter plates. Reactions were started by addition of 5 µL 1 mM MAMC (BD Biosciences—Discovery Labware, USA) in DMSO (Sigma Aldrich, USA) and fluorescence measured (excitation/emission wavelengths of 405/480 nm) using the aforementioned Synergy MX plate reader at 30 °C for 90 min in 1 min steps. OD_600_ measurements were performed in parallel as outlined above (10 µl of the cell suspensions mixed with 190 µl ddH_2_O in microtiter plates).

β-Carotene-producing strains were cultivated in shake flasks and titers determined by high-performance liquid chromatography (HPLC)^30^. Cell pellets equaling 100 OD_600_ units were mixed with 1 mL of lysis buffer (14 mM 2-mercaptoethanol, 100 mM EDTA, 1 M sorbitol). Zymolyase was added (100 µL of a 1000 U/mL stock solution) and incubated at 30 °C for 30 min resulting in spheroplasts. After pelleting (by centrifugation), the spheroplasts were resuspended in 500 μL methanol–chloroform (1:1 v/v) for carotenoid extraction and incubated for 15 min at 60 °C. To obtain complete extraction of carotenoids, this extraction procedure was repeated until the cell pellet was colorless. After drying with a stream of dry nitrogen gas, the residues were resorbed in 2 mL of methanol–chloroform (1:1 v/v) and analyzed with an Agilent Technologies 1200 series HPLC instrument (with a photodiode array detector) using a C30 carotenoid column (150 × 4.0 mm^2^, 5 μm; YMC Europe) and mobile phases H_2_O–methanol (4:96 v/v; phase A) and methanol–MTBE (5:95 v/v; phase B). The following linear gradient elution program was applied with a flow rate of 0.75 mL min^−1^ at 30 °C: 0−20 min, 5−65% phase B; 20−25 min, 65% phase B; 25.01−30 min, 65−5% phase B. β-Carotene titers were determined by external calibration using the respective reference material.

Taxadiene-producing strains were cultivated in shake flasks in BYPG media (100 mM potassium phosphate buffer pH 6.0, 1% yeast extract, 2% peptone, 1% (w/v) glycerol) with a 10% dodecane overlay and induced with methanol (final concentration of 0.5% (v/v)). Taxadiene titers were determined by gas chromatography–mass spectrometry (GC-MS)^3,70^. Briefly, a standard curve was created with purified taxadiene. Following, the dodecane overlays from the shake flask cultivations were diluted into hexanes to fall within the linear range of detection. Integration of the area under the curve for the taxadiene peak (*m*/*z* 272) and calculation for dilution factors allowed for the determination of titer value. GC-MS analysis was conducted with an Agilent Technologies 7890A GC System with a 240 Ion Trap GC/MS, 7693 Autosampler, DB-5 column (Agilent), a splitless single taper glass wool ultrainert inlet liner, and 11-mm Restek premium nonstick center guiding septa. Injections were made in pulsed splitless mode, with an inlet temperature of 250 °C and pressure of 7.652 psi. The GC method begins at 50 °C with a hold time of 1 min, followed by a 10 °C min^−1^ ramp to 200 °C (15 min), and a 5 °C min^−1^ ramp to 270 °C (14 min), for a total run time of 30 min. The post-run temperature is increased to 320 °C for 5 min between each sample. MS is collected between 30 and 350 *m*/*z* and from 17 to 30 min for each sample. The flow rate of the helium carrier gas was 1 mL min^−1^. Before each set of samples were run, washes of methanol and hexane were carried out twice. Per every six samples, an additional hexane wash was carried out. Analysis was completed by using the Agilent MS Workstation software package.

## Electronic supplementary material

Peer Review File

Supplementary Information

Description of Additional Supplementary Files

Supplementary Data 1

Supplementary Data 2

Supplementary Data 3

## Data Availability

All sequence data related to the *P. pastoris* promoters/terminators used in this study are available in the EMBL-EBI database (accession numbers FR839628–FR839632) and the gene names and promoter/terminator positions are provided in Supplementary Data 2. The majority of data generated or analyzed during this study are included in this published article (and its supplementary information files). Additional datasets generated and analyzed during the current study are available from the corresponding author on reasonable request.
