# Publisher Correction: Engineered bidirectional promoters enable rapid multi-gene co-expression optimization

**DOI:** 10.1038/s41467-018-07112-1

**Published:** 2018-10-29

**Authors:** Thomas Vogl, Thomas Kickenweiz, Julia Pitzer, Lukas Sturmberger, Astrid Weninger, Bradley W. Biggs, Eva-Maria Köhler, Armin Baumschlager, Jasmin Elgin Fischer, Patrick Hyden, Marlies Wagner, Martina Baumann, Nicole Borth, Martina Geier, Parayil Kumaran Ajikumar, Anton Glieder

**Affiliations:** 1 Institute of Molecular Biotechnology, NAWI Graz, Graz University of Technology, Petersgasse 14, 8010 Graz, Austria; 2 Austrian Centre of Industrial Biotechnology (ACIB GmbH), Petersgasse 14, 8010 Graz, Austria; 3 Manus Biosynthesis, 1030 Massachusetts Avenue, Suite 300, Cambridge, MA 02138 USA; 4 Austrian Centre of Industrial Biotechnology (ACIB GmbH), Muthgasse 11, 1190 Vienna, Austria; 5 Department of Biotechnology, University of Natural Resources and Life Sciences, Muthgasse 18, 1190 Vienna, Austria; 6 Present Address: Department of Computer Science and Applied Mathematics, Weizmann Institute of Science, 76100 Rehovot, Israel; 7 Present Address: Department of Chemical and Biological Engineering, Northwestern University, Evanston, IL 60208 USA; 8 Present Address: Department of Biosystems Science and Engineering, ETH Zürich, Mattenstrasse 26, 4058 Basel, Switzerland

Correction to: *Nature Communications*; 10.1038/s41467-018-05915-w; published online: 04 September 2018

The original HTML version of this Article included an incomplete ‘Description of Additional Supplementary Files’ which lacked legends for each file. The HTML version of the Article has been updated to include a corrected version of the ‘Description of Additional Supplementary Files’.

