# Publisher Correction: Engineered bidirectional promoters enable rapid multi-gene co-expression optimization

**DOI:** 10.1038/s41467-021-21369-z

**Published:** 2021-02-18

**Authors:** Thomas Vogl, Thomas Kickenweiz, Julia Pitzer, Lukas Sturmberger, Astrid Weninger, Bradley W. Biggs, Eva-Maria Köhler, Armin Baumschlager, Jasmin Elgin Fischer, Patrick Hyden, Marlies Wagner, Martina Baumann, Nicole Borth, Martina Geier, Parayil Kumaran Ajikumar, Anton Glieder

**Affiliations:** 1 Institute of Molecular Biotechnology, NAWI Graz, Graz University of Technology, Petersgasse 14, 8010 Graz, Austria; 2 Austrian Centre of Industrial Biotechnology (ACIB GmbH), Petersgasse 14, 8010 Graz, Austria; 3 Manus Biosynthesis, 1030 Massachusetts Avenue, Suite 300, Cambridge, MA 02138 USA; 4 Austrian Centre of Industrial Biotechnology (ACIB GmbH), Muthgasse 11, 1190 Vienna, Austria; 5 Department of Biotechnology, University of Natural Resources and Life Sciences, Muthgasse 18, 1190 Vienna, Austria; 6 Present Address: Department of Computer Science and Applied Mathematics, Weizmann Institute of Science, 76100 Rehovot, Israel; 7 Present Address: Department of Chemical and Biological Engineering, Northwestern University, Evanston, IL 60208 USA; 8 Present Address: Department of Biosystems Science and Engineering, ETH Zürich, Mattenstrasse 26, 4058 Basel, Switzerland

**Keywords:** Non-model organisms, Genetic vectors, High-throughput screening, Expression systems, Synthetic biology

Correction to: *Nature Communications* 10.1038/s41467-018-05915-w, published online 4 September 2018.

In the original version of this Article, the link for the Supplementary Information directed to the Peer Review file and the link for the Peer Review file directed to the Supplementary Information. This has now been corrected in the HTML version. The PDF version of the Article was correct at the time of publication.

