# Supplementary material for: Engineered bidirectional promoters enable rapid multi-gene co-expression optimization
Source: Nat Commun. 2018 Sep 4;9:3589. doi: 10.1038/s41467-018-05915-w (PMC6123417; doi:10.1038/s41467-018-05915-w)
Supplement: Supplementary file 3 — Description of Additional Supplementary Files [file 41467_2018_5915_MOESM3_ESM.pdf]

Description of Additional Supplementary Files

File Name: Supplementary Data 1 – List of head to head, head to tail and tail to tail genes in *P. pastoris*

Description: length: length of the intergenic region in bp; type: orientation of the two genes to each other (‘<’ and ‘>’ characters indicate the orientation arrow-like); g1-from/g1-to: begin/end of the upstream gene of the gene pair on the respective chromosome; g2-from/g2-to: begin/end of the downstream gene of the gene pair on the respective chromosome; g1-orientation: orientation of the upstream gene of the gene pair on the reverse (complement) or forward (normal) strand; g1-CDS-range: coding sequence of the upstream gene (‘join’ and multiple numbers indicate splicing events); g1-locus_tag: gene identifier containing chromosome number; g1-product: gene product of the upstream gene; g1-protein_id: accession number of the protein sequence; g1-gene: gene name (if assigned); g1-inference: protein motifs (if assigned); g1-EC_number: Enzyme Commission number (if assigned); the same terms (-orientation to -EC_number) are also given for the downstream gene (g2); inbetween: tRNA, rRNA or mobile_elements present in the intragenic region

File Name: Supplementary Data 2 - List of BDPs & primers for cloning

Description: List of primers and details on sequences used in this study. Primers used for generating the vectors applied, the *P. pastoris* nBDPs tested, sBDPs generated, BDTs tested and detailed dual gene expression and carotenoid pathway assemblies are provided. The respective information is provided in different sheets of the Excel file:

- Promoters and terminators
  - Reporter vectors
    Primers for generating the reporter vectors for bidirectional promoters and terminators are provided. Also, the primers for the generation of the entry vectors for cassettes for dual or multi gene co-expression are provided (see Materials and methods for detailed descriptions).
  - nBDPs
    Detailed list on the *P. pastoris* natural BDPs (Fig. 1c) tested and primers used for amplification. Either primers for TA cloning (shorter as no overhangs are needed) or for Gibson assembly were used (overhangs denoted in different letter case). The histone promoters were cloned in both orientations, hence two primer pairs each are listed.
  - HHX2 variants
    Details on the deletion and truncation variants of the *P. pastoris HHX2* promoter (Fig. 2c,d). The deletions were achieved by either linking two PCR products up to the deletion by Gibson assembly or by ordering the promoters as gBlocks (Integrated DNA technologies).
  - Bidirectionalization
    Overview on combinations of core promoters and monodirectional promoters used to generate bidirectionalized promoters (Fig. 3a). The core promoters were ordered as long primers and fused by PCR to the monodirectional promoter and cloned via Gibson assembly into the reporter vector.
  - Fusion promoters
    Monodirectional promoters fused to each other to generate bidirectional fusion promoters combining different regulatory profiles (Fig. 3b,c). Deletions in the *DAS1/2* promoters are described in detail in the next sheet. The promoters were amplified separately with primers (with complementary overhangs between each other and to the vector) and cloned by Gibson assembly.
  - DAS1/2 deletions
    Exact deletions performed in the monodirectional *DAS1* and *DAS2* promoters (shown in Supplementary Figure 2 and used to assemble some fusion promoters shown in Fig. 3c). The deletions were achieved by linking two PCR fragments with respective overhangs to each other using the primer combinations indicated. The olePCR products were cloned into a reporter vector via *Sbf*I and *Nhe*I sites (the *Nhe*I site is adjacent to *eGFP* reporter gene's start codon, resulting in seamless fusions).
  - Hybrid BDPs
    The exact composition of the *P. pastoris* bidirectional hybrid promoters shown in Fig. 4 is provided. Orientations of the elements are given by stylized arrows '->' or '<-', different elements are separated by '|'. The synthetic promoters were either assembled by PCR (providing short designs on a primer) or ordered as gBlocks. Fusions to the truncated HHT2-T3 variant were assembled by olePCR.
  - BDTs

Primer sequences for cloning of the bidirectional transcriptional terminators for *P. pastoris* are provided (Fig. 7, Supplementary Figure 7).

- Dual gene applications
  Primers for generating the bidirectional dual gene co-expression vectors and cloning of the BDPs tested in *P. pastoris* for Taxadiene production, CYP2D6+CPR co-expression and CalB+PDI co-expression (Fig. 6a-c) are provided.

- Carotenoid pathway constructs
  Contains the exact promoters and terminators used for the pathways shown in Fig. 8a. Primers for cloning via Gibson assembly are indicated (n.a. = not applicable).

- Carotenoid pathway primers
  Primer sequences for assembling the constructs shown in the aforementioned sheet.

File Name: Supplementary Data 3 - Annotated sequences for minimal set of promoters

Description: Excel file containing annotated sequences of a minimal set of BDPs covering broad regulatory profiles for dual gene expression optimization. Annotated sequence files in GenBank format are provided for the BDPs highlighted in Tab. 1 of the main manuscript.
