# Supplementary material for: Engineered bidirectional promoters enable rapid multi-gene co-expression optimization
Source: Nat Commun. 2018 Sep 4;9:3589. doi: 10.1038/s41467-018-05915-w (PMC6123417; doi:10.1038/s41467-018-05915-w)
Supplement: Supplementary file 2 — Supplementary Information [file 41467_2018_5915_MOESM2_ESM.pdf]

Reviewers' comments:

Reviewer #1 (Remarks to the Author):

The authors here report on design, expression testing and use of a collection of bidirectional gene promoter modules for Komagataella phaffii, an often-used expression system for recombinant proteins. Some of these promoters are derived from histone genes of the organism, as it was indeed already known that the orthologs in other organisms, in particular in the yeast Saccharomyces cerevisiae, are bidirectional. This is confirmed here for Komagataella. Other 'bidirectional promoters' are constructed by simple head-to-tail cloning of 2 known promoters with known transcriptional regulation patterns. It comes as no great surprise that then these promoters function in the same way when they are cloned head-to-tail rather than when used in isolation. The concept of using bidirectional promoters in biotechnology is not novel, as the authors indeed also indicate by citing relevant papers. These are set away as 'preliminary', but there are e.g. two rather substantial Nature Biotechnology papers among the cited ones that I'd hardly call 'preliminary' after having a look at these...
On the level of conceptual novelty, I would consider the paper as rather limited.

The material that is presented in this paper is however certainly useful for the practitioner of protein expression/synthetic biology in this particular organism, and it adds to the gene regulatory element collection that is available, with some advantages in terms of cloning efficiency, as the authors also show.

I have only few doubts about the technical validity of the experiments and a huge amount of work is presented, it must have been an enormous job to collect all of this; one may argue that it is too much for one paper. In terms of presentation, it looks like a quick digest has been made from one or more chapters of a doctoral thesis, with some elements put together in the main text and the remainder having been kept for the supplementary material. This did not benefit clarity nor brevity and I was a bit at a loss as to what now the main message/guidance/conclusions were for the mass of data that is presented. In particular, in the main text there is virtually no mention of experiments in CHO and S. cerevisiae, but all of a sudden these appear with very little context at all in the supplementary material. I have no idea why that was and found it impossible to comprehend.

Therefore, an important remark on the paper is that it needs to be carefully rewritten for clarity, not trying to overwhelm the reader with data but rather presenting the key experiments that allow the authors to now suggest (hopefully) the minimal bidirectional promoter set that together encompass the key variables in their design (constitutive/Methanol inducible, with varying strength). Few readers will want to start utilizing 168 promoters of which many are rather similar in behaviour. The main utility of a paper like this is if it can condense all of the obtained data to clear instructions on a minimized set of tools that readers can then confidently use, knowing that they will miss very little opportunities for further enhancement in their designs. The supplementary materials section needs to be more carefully designed, linking each and every section of it tightly to the buildup of the main text such that it becomes more easy to follow. Each piece of material is technically quite OK but the integration in the overall story needs to be enhanced. Perhaps an experimental flowchart for the overall study would help to guide the reader: what is the sequence of questions that was explored, and where can one find the data for each piece?
I would suggest to keep the paper limited to Komagataella phaffii only, as the few other experiments are adding not much and should better be elaborated upon in future papers.

A few experimental and technical points:

1) In the experiment section that deals with testing the promoters for a few applications (page 5 and onwards), some controls appear to be needed. For Komagataella phaffii, the default since decades is to use the AOXI or GAP promoter for obtaining inducible or constitutive expression,

respectively. As reconfirmed in the present study, these are still amongst the very strongest such promoters for the organism. Consequently, I feel that control experiments in which the modules are driven by these promoters in classical unidirectional constructs are important to be able to evaluate whether the bidirectional setups have any supplementary value in terms of obtained yields of the targeted products. Such controls are only there for the beta-carotene example, making it difficult to assess whether this is generalizable.
2) In these real-life engineering examples, I was surprised to see only a small set of the promoters being tested. Why make 168 first and then limit to just a dozen in the first real use, especially given that, rather surprisingly,this selection of a dozen is heavily biased to very 'classical' promoters, with the long-known DAS1-DAS2 bidirectional couple, and combinations of the well-known GAP, AOXI, TEF and CAT promoters dominating the design. The paper (see abstract, e.g.) makes a lot of the inspiration by bidirectional histone promoters, but only two of those are included in the real-life testing, with actually rather dismal results (no production yields at all, or much worse than the classical promoter combinations). These observations would reinforce the point that controls with simple constructs using AOXI and GAP or TEF are needed.
3) When working with Komagataella, constructs are typically genomically integrated. This was also done here but I could find no details as to where the vectors were linearized and to what locus they were designed to be biased for integration. Depending on the selection marker used (I think I did not see that information), multi-copy integration occurs more or less frequently, which can fundamentally obscure interpretations in terms of the relation between promoter used and expression yields obtained. Gene copy number can rather straightforwardly be determined using either qPCR or Southern analysis. Given the scale of experimentation used here, I would agree that copy number controls are difficult to do for every single reported experiments, but I definitely think that these controls are essential for those experiments that lead to key conclusions, e.g. in the pathway engineering examples. What precautions did the authors make to ensure that all of these yeast clones had single copy integration at a particular target locus?
4) What is meant by 'cumulative expression'? This is not such a common term and needs to be defined.
5) Same for 'expression efficiency'. I see that it is something like expression level per bp of promoter sequence. That is again a rather uncommon way of putting things and I doubt that it is meaningful. Very short promoters can be tremendously powerful and vice versa. What users care about is the expression yield obtained by a promoter, the length of it is quite secondary. I was misled by some of the figures, thinking that I was looking at stronger or weaker promoters, whereas what was displayed was this odd promoter strengths/bp thing. I suggest to remove these figures and stick to promoter strength.

Reviewer #2 (Remarks to the Author):

The manuscript by Vogl and colleagues describes the development and application of a large library of natural and synthetic bidirectional promoters for tuned gene co-expression in Pichia pastoris. The work involves extensive characterization of natural bidirectional promoters, construction of synthetic bidirectional or hybrid promoters, and development and analysis of bidirectional terminators, to establish an extensive tool-set for controlling gene expression in P. pastoris. The authors also present an efficient cloning strategy for the rapid screening of the promoter library on any given gene pair. Furthermore, they confirm the function of some of the identified bidirectional promoters in other yeast or mammalian cells. Finally, they validate the usefulness of the tools developed using several case studies.
The work is thorough, and the volume of promoters constructed and analyzed is impressive. The resource established will be very useful for numerous synthetic biology efforts in P. pastoris and will inspire similar efforts in other synthetic biology chassis. The findings have broad interest for the synthetic biology community and the manuscript is suitable for the audience of Nature Communications. However, the authors should address the following issues:

1. In the manuscript title, but also in the abstract and the conclusions, the authors place special emphasis on the histone bidirectional promoters, and they also make a case for generalization of their findings based on the conservation of the histone BDP architecture between P. pastoris and other eukaryotes. Although several hybrid histone BDPs developed perform well in the fluorescent protein coupled reporter assay developed, neither the native or the hybrid histone BDPs prove to be so efficient when tested in “real-life” applications in the case studies used. In the taxadiene, Cyp2D6, or CalB case studies, optimal performance was achieved with native inducible promoter combinations, rather than inducible or constitutive histone-based promoters. Only in the carotenoid production case study have the histone hybrid promoters performed better, but the improvement over the simple linear gene arrangement under the inducible AOX1 promoter is only 2-fold, suggesting a very limited overall contribution of expression tuning in the performance of this system. Furthermore, the requirement of all case studies for inducible expression diminishes the usefulness of the non-hybrid histone BDPs. Although it is possible that the histone promoter-based hybrid variants will eventually be beneficial in certain cases, there is very little evidence so far for the authors to argue that the main benefits/novelty of their system stems from the architecture of the histone BDPs. The promoter resource developed here is a powerful tool in its own right and will have a strong impact in future synthetic biology applications, but the main advantage of the approach is the size and extent of the promoter collection and the construction of several synthetic BDPs with varied regulation. This is evidenced by the observation that a different combination of promoters was found to be optimal in each of the first three case studies, which makes a compelling case for testing such a large and diverse library of promoters as a standard strategy in similar synthetic biology applications. The authors should consider rephrasing this main claim to better reflect the findings.
2. The authors should also clarify in their abstract and discussion the extent by which the specific resource can be directly transferred to another chassis. Although the architecture of histone promoters is indeed conserved, in the case studies presented here the most successful promoter combinations turned out to be inducible. Since the methanol utilization capacity of P. pastoris is rather unique, for the hybrid histone BDPs-based promoters to be useful in another system, these must be redesigned and re-constructed using host-specific inducible promoters. Therefore, the generality of the approach lies in the overall strategies employed for the construction of the promoter library (and the lessons learned), rather than the ability to transfer a large collection of parts to other hosts.

Minor comments:
3. To facilitate evaluation of the results of the case studies, it would be useful to include in the figures 4a, b, and c the corresponding normalized expression (based on the fluorescent protein-based assay) of each of the BDPs together with the titer/activity (using a second axis and symbol on the same graph).
4. The taxadiene titer obtained in the optimal BDP is indeed impressive, considering that the P. pastors strain used here is not engineered for isoprenoid production. However, the S. cerevisiae strain used for comparison in ref 37 is not a heavily engineered strain (line 203). An example of such a strain can be the one mentioned in (Westfall 2012, PNAS doi: 10.1073/pnas.1110740109). Please rephrase.
5. In Figure 4d it is not so easy to read the identity of the different promoters/terminators.
6. Fig S1, and many other figures in the supplementary section are difficult to read. As there is not a tight size restriction in this section, the authors could try to help the reader by increasing the size of the diagrams.
7. A lot of valuable information has been relegated to the supplementary section, presumably due to size considerations (one such example is in lines 411-416 in supplementary, but several instances exist). The authors should review the size of the manuscript and explore the possibility to bring some arguments/clarifications forward to main text.
8. Line 492 in supplementary “Fold-differences….”. Misplaced sentence?
9. Line 441 in suppl. Correct to: cytomegalovirus.

Reviewed by:

Sotirios C. Kampranis, PhD
Associate Professor
University of Copenhagen, Denmark

Reviewer #3 (Remarks to the Author):

The work by Vogl et al describes the construction and characterization of 168 new synthetic promoters for Pichia pastoris with bidirectional activity. The work starts by bioinformatic analysis to identify natural bidirectional promoters and then starts the construction and evaluation of several variants though the combination of a number of characterized modules. Additionally, synthetic terminators are also evaluated in the work. Finally, the authors demonstrate the usage of the nearly developed tools for the optimization of biosynthetic pathways.

In general I found the manuscript very interesting and certainly it should have an important impact in the field. Yet, I find the manuscript quite confusing sometimes by several reasons, as listed below.

Major comments

1. There are two manuscripts in one here (The main text and the supporting information). The main text describes very directly (and fast) the main results of the work. For example, an entire figure (Figure 3) is presented and discussed in less then 5 lines (176-178 and 185-186). This makes difficult to fallow the work in light of the heave load Figures presented (specially Figure 2). In fact, there is a back and forth movement into the Figure 2. The authors should try to enhance a bit the presentation of the data and discussion of the results. In the case of Figure 2, this could be dived into two Figures, even by esthetic reasons. The letters in the figure are very small and only by zooming it I could follow which was each promoter constructed. I do not think it will be easy for a reader to follow it in a printed version of the paper. In the case of the supporting information, there is a extensive discussion on a number of control and additional experiments, with a lot o interpretations and expectations in some cases. This section could be simplified, with some of the critical discussion been incorporated to the main text. In fact, I have learned quite a bit more reading the SI then the main text.

2. The experiments in S. pombe, S. cerevisiae and CHO cells are presented in the Material and Methods (lines 532 to 607) and presented very briefly in the conclusions. I do not see the point of this here. The flow bioinformatic singing-promoter engineering-pathway construction makes this manuscript worth already. Yet, if the authors think this is relevant to be present in the conclusion, then they should consider properly present and discuss the results in the previous section, with a proper Figure and conclusive data. Otherwise, removing this part would make the work less confusing. I particularly think the paper will be great even without this potential generalization.

3. The authors present a number of muted version of the promoters, some by creating deletions others by fusing different parts. These variants present different behaviors in terms of indelibility and activity levels. Yet, there was not assessment of the single-cell behavior of the resulting promoters. The authors argued that a Flow Cytomiter with appropriate filters for dual analysis was not available at the institution. Yet, a typical 488 filter would allow then to investigate using GFP only whether the modulation of promoter activity by some critical modifications is the result from changes in the single-cell behavior of the promoters (such as in Fig. 2b,c and d). This is very easy to do with the promoters already constructed and is critical to see if the new tools are indeed useful for pathway engineering. If the resulting promotors are switching toward a all-or-none behavior, that would mean that the genes of the engineered pathways would be expressed at different cell population. Perhaps that would explain why the strain expression taxadiene did not bit the S. cerevisiae strain. This kind of characterization is crucial for a manuscript of this

magnitude.

4. The authors used eGFP and dTomato to quantify promoter activity. They presented int he SI a investigation on the differences in signal produced by the two reporters (page 9). Yet, this information is very relevant and should be clearly presented in the main text. In other words, are the RFU/OD600 data presented along the manuscript corrected using the data provided in page 9 of SI?

5. Related to the previous point, the used of GFP, RFP and OD600 quantification can lead to some interference in signal as demonstrated by Hecht et al 2016 (https://pubs.acs.org/doi/abs/10.1021/acssynbio.6b00072). The same approach is used here. Have the authors checked these potential interferences here? This should be clearly demonstrated and discussed.

6. A strong weight is placed on Figure 2. Since glycerol, glucose and methanol are used to induce of repress promoter activities, it should be better presented in which cases the promoters are expected to be constitutive and in which a induction/repression is expected to occur.

7. The way the new P. pastoris strains performs compared to previous attempts should be better presented (with the actual values), such as in line 202 and 227.

5. How easy would be for the public to reuse some of the new parts created here? How some would reproduce the experiments using the engineered promoters. The tables provided the primers used for construction of the promoters, but the authors should consider to provide some annotated sequences that could be directly synthetized and used, in cases where it does not conflicts with the existing patents. Otherwise, it will be not easy to fallow those instruction and reproduce the experiments.

Minor comments

1. As I said before, the SI material is quite dense. Please revise it for potential errors in information (such as "Pp hybrid promoter"in line 226 vs "Pp hybrid BDPs in the Excel table). There are different style in the text, such as eGFP vs. eGfp.

2. Page 9. I do not see how FRET fits here, since there is not reason for the two proteins be in close proximity for this to occur. No need for this speculation.

3. Page 26. What do the authors understand for "more energy"? Is it reducing power? ATP? There are some good literature on resource allocation and metabolic burden that can be used here.

4. Page 26. What is CYP (line 775)?

General response

We would like to thank all three reviewers for their detailed comments, constructive criticism and insightful suggestions to improve the manuscript. We have revised the manuscript accordingly and made several changes (listed below).

A universal point of criticism mentioned by all reviewers was the duality between the main text and supporting information. We have reworked the manuscript and transferred key information from the SI to the main text resulting in a more coherent manuscript.

Especially reviewers 1 and 3 commented on the breadth of the manuscript suggesting to remove the results on histone promoters in other organisms than *P. pastoris*. We have removed the data on histone promoters in *S. cerevisiae*, *S. pombe* and CHO cell lines from the manuscript/SI. Therefore the author list has changed and R.W., A-M.H. and K.W., who performed the respective experiments, are no longer listed as authors in the revised manuscript.

While unanimously acknowledging the usefulness of the BDP library, another shared point of criticism by the reviewers was towards the role of the histone promoter based engineering strategy and the applicability of the histone promoters themselves. We have hence removed 'histone promoters' from the title and revised the interpretation to limit the role of histone BDPs in the abstract and throughout the manuscript, still highlighting their usefulness as parts repository for promoter engineering.

We have also replied to all reviewers' comments in detail, see the point by point responses below.

Two versions of the manuscript are provided: 1) a version highlighting the changes compared to the initial submission; 2) a 'clean' version.

List of major changes

- removed previous supplementary figures S6 (histone promoters in different organisms) and S7 (*P. pastoris* histone prompters on glucose and glycerol) and combined relevant parts thereof in the main manuscript (revised Fig. 2 and new sub-section "Bidirectional histone promoters as useful parts repository")
- old figure 2 split into three figures to improve readability (new figures 2, 3, 4)
- new figure 7 on bidirectional terminators added (moved from old supporting information S11), text in new S10 shortened and moved essential parts to the main manuscript
- controls of monodirectional reference promoters also provided for dual gene co-expression examples (Fig. 6b,c)
- supporting figure S1 from the initial manuscript and extended discussion thereof shortened and repositioned in the SI (now S8)
- old supporting text S 10 (extended discussion on the applications) has been deleted and key information incorporated in the main manuscript.
- removed data on histone promoters in *S. cerevisiae*, *S. pombe* and CHO cells from the main text and SI (old S12 was removed)
- The only longer text stretches left in the supplements are extended discussions on the fluorescent protein measurements (S4), promoter design (S6, S7) and cloning strategy (S8). Due to the length limit, we were unable to include them in the main text, but we believe that readers interested in detailed promoter architecture/design and practical applications will find them useful.

Table of contents

General response 1
List of major changes..... 1
Table of contents..... 2
Response to Reviewer #1 3
Response to unnumbered comments 3
Response to numbered comments 5
Response to Reviewer #2 11
Major comments 11
Minor comments: 13
Response to Reviewer #3 16
Major comments 16
Minor comments 22
References for response to reviewers 23

Response to Reviewer #1

Response to unnumbered comments

“The concept of using bidirectional promoters in biotechnology is not novel, as the authors indeed also indicate by citing relevant papers. These are set away as 'preliminary', but there are e.g. two rather substantial Nature Biotechnology papers among the cited ones that I'd hardly call 'preliminary' after having a look at these... On the level of conceptual novelty, I would consider the paper as rather limited.”

We agree that these are important milestone studies and we had not intended to downplay their significance. The term ‘preliminary’ was only related to the number of promotes and the library concept of the present studies. The two Nature Biotechnology paper report less than 10 bidirectional promoters and the biggest collection known in the literature (for *S. cerevisiae* (Öztürk et al., 2017)) are also less than a dozen BDPs, whereas our library of 168 BDPs is considerably larger. Previous BDPs reported in the Nature Biotechnology papers mentioned or *S. cerevisiae* also do not cover different expression ratios and regulatory profiles allowing consecutive induction (as the derepressed/inducible promoters in this paper). The novelty of this work does not lie in the use of bidirectional promoters *per se*, but the library, cloning and design concepts of using differently regulated BDPs to fine-tune gene expression, which provides several advantages over monodirectional promoters or conventional bidirectional systems.

We have reworded the text in the manuscript to correct this ambiguity towards previous work:

“Inspired by these circuits, biological engineers have recently utilized BDPs to improve designs for gene co-expression in *Escherichia coli* (Yang et al., 2012), *Saccharomyces cerevisiae* (Öztürk et al., 2017), plants (Xie et al., 2001), and mammals (Amendola et al., 2005; Fux and Fussenegger, 2003). These ~~**preliminary**~~ studies offer promise, but **larger sets** of readily available BDPs remain limited, and the reported strategies have lacked generalizability. **To our knowledge, *S. cerevisiae’s* less than dozen BDPs represent the largest collection** (Öztürk et al., 2017) **and do not provide the desired spectrum of different expression ratios or consecutive induction.**”

We have also extended and revised the discussion on the library concept, cloning strategy and transferability of the design strategies to different organisms in the main text.

“I have only few doubts about the technical validity of the experiments and a huge amount of work is presented, it must have been an enormous job to collect all of this; one may argue that it is too much for one paper. In terms of presentation, it looks like a quick digest has been made from one or more chapters of a doctoral thesis, with some elements put together in the main text and the remainder having been kept for the supplementary material. This did not benefit clarity nor brevity and I was a bit at a loss as to what now the main message/guidance/conclusions were for the mass of data that is presented. In particular, in the main text there is virtually no mention of experiments in CHO and S. cerevisiae, but all of a sudden these appear with very little context at all in the supplementary material. I have no idea why that was and found it impossible to comprehend.” […]

“I would suggest to keep the paper limited to Komagataella phaffii only, as the few other experiments are adding not much and should better be elaborated upon in future papers.”

As outlined in the general response above and in more detail below, we have reworked the manuscript moving parts from the supplements to the main text and also removing the results on the experiments in *S. cerevisiae, S. pombe* and CHO cells (Reviewer 3 had made a similar suggestion).

“Therefore, an important remark on the paper is that it needs to be carefully rewritten for clarity, not trying to overwhelm the reader with data but rather presenting the key experiments that allow the authors to now suggest (hopefully) the minimal bidirectional promoter set that together encompass the key variables in their design (constitutive/Methanol inducible, with varying strength). Few readers will want to start utilizing 168 promoters of which many are rather similar in behaviour. The main utility of a paper like this is if it can condense all of the obtained data to clear instructions on a minimized set of tools that readers can then confidently use, knowing that they will miss very little opportunities for further enhancement in their designs.”

Reviewer 3 had a similar comment (also on where to obtain annotated promoter sequences). We have now added a table with a set of 12 (if tested in both orientations) BDPs representing key features of our library (and included also annotated sequence files for these promoters to make them easily accessible to other researchers). The table is included in the main manuscript (Table 1), the sequence files as S3 (in GenBank format).

“Perhaps an experimental flowchart for the overall study would help to guide the reader: what is the sequence of questions that was explored, and where can one find the data for each piece?”

As mentioned in the last comment, we have now added a summarizing table and annotated promoter sequences for a minimized set of promoters. We are also happy to add a flowchart suggested by the reviewer, but at the moment are not sure what this should depict exactly. We have now repositioned the figure on the cloning strategy in the SI (new S8) and rewritten the discussion of these results. Together with the summarizing panel A of Figure 1, this should sufficiently illustrate the workflow. Or should we add another type of flowchart?

The data for all promoters/terminators are shown in the respective figures and detailed information on cloning etc. is provided in the supporting Excel file (S2). To make the link between figures and the sheets in the supporting excel file more clear, we have now added references to each figure in the SI (for example the for the natural BDPs, a link to Fig. 1c is provided, for the deletion/truncation variants to Fig. 2c,d etc.).

"The supplementary materials section needs to be more carefully designed, linking each and every section of it tightly to the buildup of the main text such that it becomes more easy to follow.”

We have reworked the structure of the manuscript, by in part moving parts from the supplements to the main text and completely removing other parts. Thereby the SI has be shortened and now better follows the course of the main text. Key information on the advantages of histone BDPs and applications is now provided in the main text and has been removed from the supplements. The only extended discussions in the supplements are on detailed results on the fluorescent protein measurements, promoter design and BDP cloning. Due to the length limit, we were unable to include them in the main text, but we believe that readers interested in detailed promoter architecture/design and practical applications will find this information useful.

“Other 'bidirectional promoters' are constructed by simple head-to-tail cloning of 2 known promoters with known transcriptional regulation patterns. It comes as no great surprise that then these promoters function in the same way when they are cloned head-to-tail rather than when used in isolation.”

Interestingly, the two fusions promoters did not behave exactly the same way: Some fusion variants showed synergistic (boosting expression of up 1.8-fold in case of a *GAP-DAS2* fusion promoter) or antagonistic effects (40% repression of a *HTA1-TAL2* fusion promoter) suggesting a transcriptional ‘spillover’ between promoters. These findings contrast previous MDP fusion studies in *S. cerevisiae* (Da Silva and Srikrishnan, 2012; Li et al., 2008; Miller et al., 1998; Öztürk et al., 2017; Partow et al., 2010; Vickers et al., 2013), potentially due to the greater number of promoters and combinations tested here in our work. It is known that binding of insulator proteins can decouple regulation of BDPs per side in *S. cerevisiae* (Yan et al., 2015), and thus the exact properties of fusion promoters are difficult to predict.

These results had initially only been shown in the supplements and briefly mentioned in the main text. We have now extended their description in the main text.

Also for bidirectionalization (where only a short core promoter, rather than a full length monodirectional promoter is fused to a monodirectional promoter) our results led to new insights compared to previous work: In bidirectionalization studies in higher eukaryotes (Amendola et al., 2005; Xie et al., 2001), testing a few promoters in a single length led to suitable BDPs. Here, we had to test several promoters in different lengths with different core promoters to obtain strong bidirectionalized promoters (Fig. 3a). These dissimilarities may be explained by a different function/distance relationship between regulatory regions from yeast and higher eukaryotes.

“The material that is presented in this paper is however certainly useful for the practitioner of protein expression/synthetic biology in this particular organism, and it adds to the gene regulatory element collection that is available, with some advantages in terms of cloning efficiency, as the authors also show.”

To highlight these advantages, we have repositioned the figure on the cloning strategy in the SI and have also rewritten and restructured the discussion on the applications.

Response to numbered comments

“1) In the experiment section that deals with testing the promoters for a few applications (page 5 and onwards), some controls appear to be needed. For Komagataella phaffii, the default since decades is to use the AOXI or GAP promoter for obtaining inducible or constitutive expression, respectively. As reconfirmed in the present study, these are still amongst the very strongest such promoters for the organism. Consequently, I feel that control experiments in which the modules are driven by these promoters in classical unidirectional constructs are important to be able to evaluate whether the bidirectional setups have any supplementary value in terms of obtained yields of the targeted products. Such controls are only there for the beta-carotene example, making it difficult to assess whether this is generalizable.”

We have now generated also monodirectional controls for the dual gene coexpression examples and added the data to Figure 6.

For CYP2D6/CPR we have used two monodirectional *AOX1* promoters, each cloned separately upstream of the CYP2D6 and CPR genes respectively. For CalB/PDI we have mimicked the best BDPs [P_{CAT1}/P_{AOX1} and P_{CAT1}/P_{GAP}]. For CYP2D6/CPR, the best bidirectional promoter ($P_{DAS1\text{-}DAS2}$ in a specific orientation), surpasses two monodirectional *AOX1* promoters about two-fold. Yet, the two monodirectional *AOX1* promoters perform similar to bidirectional fusions of the *AOX1* and *CAT1* promoters (which are roughly of similar strength) (Fig. 6b). Also recreating the strongest BDPs ($P_{CAT1\text{-}AOX1}$ and $P_{CAT1\text{-}GAP}$) for CalB-PDI co-expression with monodirectional assemblies (P_{CAT1} for CalB and P_{AOX1} or P_{GAP} for PDI) matches the bidirectional design (Fig. 6c).
These results illustrate that once the optimal expression profile is known from BDPs, similar expression can be reproduced using MDPs (at least for fusion BDPs). However, there is no simple strategy available to insert two MDPs at the same time and hence it is not feasible to perform similar screenings for dual gene expression optimization with MDP libraries (S8). We assume that similar effects would also occur for taxadiene production, *i.e.* the high levels from the bidirectional *GAP-CAT1* fusion promoter could be mimicked by using monodirectional P_{GAP} and P_{CAT1} promoters to drive expression of the respective *TDS* and *GGPPS* genes. Yet, practically cloning this into conventional monodirectional vectors was very challenging due to the long TDS gene (2409 bp)s and restriction sites present (the cassettes would have to be stitched together by several overlap extension PCRs), highlighting the superior strategy of using BDPs, requiring only a single restriction digest to clone an entire library of BDPs (S8).
We are not claiming that the MDPs cannot reach similar expression levels as BDPs, the use of BDPs however greatly facilitates cloning efforts and thereby co-expression fine-tuning (S8). Also, while MDPs and BDPs can show similar strengths, the short length of BDPs (and accompanying high relative expression efficiency Fig. 5c) is advantageous, especially for pathway applications.

We have added these results and a brief discussion to the revised version of the manuscript (renamed section "The library of BDPs facilitates dual gene coexpression optimization").

"2) [first part] In these real-life engineering examples, I was surprised to see only a small set of the promoters being tested. Why make 168 first and then limit to just a dozen in the first real use, especially given that, rather surprisingly,this selection of a dozen is heavily biased to very 'classical' promoters, with the long-known DAS1-DAS2 bidirectional couple, and combinations of the well-known GAP, AOXI, TEF and CAT promoters dominating the design."

The screening strategy of using subsets promoters, rather than the entire library of 168 promoter, is related to the next question (3) of the reviewer about biases of genomic integration and copy numbers fundamentally obscuring interpretations.

This is exactly what would happen if a library of 168 promoters were to be transformed into *P. pastoris*: Even when using low amounts of DNA for the transformations, multi copy integration cannot be entirely avoided (Vogl et al., 2018a). Also due to a relatively strong NHEJ mechanism, a substantial number of transformants contains ectopically integrated expression cassettes (Schwarzhans et al., 2016a, 2016b; Vogl et al., 2018a; Weninger et al., 2018, 2016). Even for the most efficient integration sites and even when using CRISPR-Cas systems, in the best case only up to ~60% of cassettes integrate specifically in the *P. pastoris* wildtype strain (Vogl et al., 2018a; Weninger et al., 2018).

Hence, when transforming the entire library, not only the promoter choice but also copy numbers and integration sites would influence the outcome. Ultimately this does not matter for biotechnological

applications, where yields should be maximized and the possibility of multi copy integration is even a powerful advantage of the *P. pastoris* system (Aw and Polizzi, 2013).

However, we wanted to prove here that the choice BDPs has a strong effect on expression, which could not be easily done with pooled libraries. Hence, we designed experiments to rule out confounding effects of multiple copies and different integration sites. We cloned each of the constructs shown in the application figure (Fig. 6) separately, transformed *P. pastoris* cells separately and screened for each construct 42 transformants (approximately half a deep well plate), picked representative clones form the middle of the expression landscape (thereby avoiding copy number/integration outliers) and rescreened them (following a published workflow demonstrating that this approach leads to similar results as checking for the integration locus and copy number (Vogl et al., 2018a)). This could not be done with a pooled library of 168 promoters and clonal variation would mask the effects of the promoters. Moreover, all of the promoter/terminator characterizations had been performed this way, screening 42 transformants, followed by rescreening.

Regarding the choice of very 'classical' promoters, we used constitutive, inducible and derepressed promoters to illustrate the propagated effect of expression strength, regulation and ratios for fine-tuning. When using a pooled library finer increments of expression can be tested. Alternatively, after having identified a promising promoter choice in the first round (such as Figure 6), an additional round with variants related to this promoter can be performed.

In response to the reviewer's comment on "the minimal bidirectional promoter set that together encompass the key variables in their design (constitutive/Methanol inducible, with varying strength)", these promoters represent such a set accurately. As outlined above, we have added table and discussion highlighting these promoters to the main text.

"[2] [second part] The paper (see abstract, e.g.) makes a lot of the inspiration by bidirectional histone promoters, but only two of those are included in the real-life testing, with actually rather dismal results (no production yields at all, or much worse than the classical promoter combinations). These observations would reinforce the point that controls with simple constructs using AOXI and GAP or TEF are needed."

Reviewer 2 presented a related comment on the applicability of the histone promoters themselves. We have hence removed 'histone promoters' from the title and revised the interpretation to limit the role of histone BDPs in the abstract and throughout the manuscript (still highlighting their usefulness as parts repository for promoter engineering).

To respond in detail to the comment by reviewer 1: The seemingly poor performance of the histone promoters is in part caused by the protein targets (TDS/GGPPS, CYP2D6/CPR). Constitutive expression of TDS/GGPPS and CYP2D6/CPR apparently causes too much cellular stress, hence the constitutive/growth associated histone promoters did not yield colonies after transformation. The same issue holds for the constructs bearing the suggested GAP or TEF promoters, as we obtained no transformants here either. Hence, this is not a limitation of the histone promoters specifically, but rather all constitutive promoters for these gene pairs.

For both CalB/PDI and the carotene pathway, the histone promoters and *GAP* reference promoter did give expression. However, the derepressed and methanol-inducible *CAT1* promoter, induced with methanol, outperformed any constitutive promoter (the histone promoters or P_{GAP}) for CalB/PDI. The best histone

promoter (*HTA1-HTB1*) gave fair expression compared to P_{GAP}, reaching about 50% on glucose. Nonetheless, constitutive expression appears unfavorable compared to methanol induction for CalB/PDI expression.

For the carotene pathway, the best bidirectional design based on histone promoters (C11) yielded 14.9-fold higher β-carotene titers than the monodirectional standard P_{GAP} design. Hence, in this case the histone promoters performed substantially better than the *GAP* reference promoter. Methanol inducible BDPs yielded a further two-fold improvement. It is noteworthy that the histone strains did not need to be induced with methanol and were harvested at 60 hours, whereas the methanol induced construct was grown for additional 48 hours. Thus, in terms of space/time yields, the histone promoters provide an advantage and may be useful, especially in large scale production plants where induction with toxic and flammable methanol can represent a considerable safety risk.

"3) When working with Komagataella, constructs are typically genomically integrated. This was also done here but I could find no details as to where the vectors were linearized and to what locus they were designed to be biased for integration. Depending on the selection marker used (I think I did not see that information), multi-copy integration occurs more or less frequently, which can fundamentally obscure interpretations in terms of the relation between promoter used and expression yields obtained. Gene copy number can rather straightforwardly be determined using either qPCR or Southern analysis. Given the scale of experimentation used here, I would agree that copy number controls are difficult to do for every single reported experiments, but I definitely think that these controls are essential for those experiments that lead to key conclusions, e.g. in the pathway engineering examples. What precautions did the authors make to ensure that all of these yeast clones had single copy integration at a particular target locus?"

Regarding the general screening/characterization strategy:

This issue has partially been addressed in the response to the first part of comment (2) by the reviewer. In short, all vectors used in this study contained a Zeocin resistance marker and were based on the pPpT4 plasmid family reported by (Näätsaari et al., 2012) and more specifically reporter vectors previously used to characterizes dozens to hundreds of promoter variants following an established screening procedure (Portela et al., 2018, 2017, Vogl et al., 2016, 2014).

A detailed explanation on the vectors and on the screening strategy has been added to the materials and methods section (the initial version had only contained references to the established vectors/protocols).

Namely, all vectors used in this study contain integration sequences near the *ARG4* locus and were linearized with *Swa*I to target insertion near the *ARG4* locus, as applied in previous promoter characterizations in *P. pastoris* (Portela et al., 2018, 2017; Vogl et al., 2016).

P. pastoris cells were transformed with molar equivalents to 1 µg of the empty pPpT4_S vector *Swa*I linearized plasmids (Lin-Cereghino et al., 2005), as 1 µg of the empty pPpT4_S vector was found to yield predominantly single copy integration (Vogl et al., 2018a, 2014). Some of the vectors used in this study are however considerably larger than the empty pPpT4_S vector [*e.g.* the carotenoid pathway constructs], hence in these cases we increased the DNA amounts to have an equivalent number of vector molecules compared to the empty pPpT4_S vector.

The screening and rescreening procedures to compare single *P. pastoris* strains have previously been reported (Vogl et al., 2016, 2014). In brief, for each construct 42 transformants (approximately half a deep well plate) were screened to account for clonal variation observed in *P. pastoris* (Schwarzhans et al., 2016a, 2016b; Vogl et al., 2018a). Three representative clones from the middle of the obtained expression landscape (to avoid outliers of multi copy integration or reduced expression because of deletions (Vogl et al., 2018a) or undesired integration events (Schwarzhans et al., 2016a, 2016b)) were streaked for single colonies and rescreened in biological triplicates. Finally, one representative clone was selected and a final screening of all the variants together was performed.

Regarding copy numbers and "*What precautions did the authors make to ensure that all of these yeast clones had single copy integration at a particular target locus?*"

This tedious screening, rescreening and final screening workflow eliminates not only the occurrence of different copy numbers but also biases of different integration sites. A detailed mechanistic study thereof for this exact plasmid design has recently been published (Vogl et al., 2018a). By transforming 1 µg of DNA, predominately (>95%) single copy integration is obtained (why still maintaining reasonable transformation efficiencies). By then picking only transformants from the middle of the landscape, we avoided working with multi copy strains (that would represent the highest expressing transformants in the screening). We have also determined the copy numbers in previous studies to verify this strategy (Vogl et al., 2018a, 2014) and have already used the same approach to characterize dozens to hundreds of promoter variants (Portela et al., 2018, 2017, Vogl et al., 2016, 2014).

The results obtained in this study illustrate the robustness of this approach. For example, the histone promoters show the same expression no matter if they are cloned in forward or reverse orientation (after correcting for the use of different fluorescent proteins [supporting figure S4]). In addition, in bidirectionalization experiments, where different core promoters are fused to the same monodirectional promoter (Fig. 3a), the expression of the constant/monodirectional side stays the same, and generally only the expression on the second side with the new core promoters changes.

This holds not only true for the experiments with fluorescent proteins, but also for the validation experiments of the real world examples, that the reviewer asked for in a previous comment. When we recreated the same promoter construct with MDPs our chromosomal integration approach gave the same expression results, demonstrating its consistency. (Fig. 6b,c).

Hence the characterization approach applied in this study yields reliable results, avoiding biases of copy numbers or different integration sites.

"4) What is meant by 'cumulative expression'? This is not such a common term and needs to be defined.
5) Same for 'expression efficiency'. I see that it is something like expression level per bp of promoter sequence. That is again a rather uncommon way of putting things and I doubt that it is meaningful. Very short promoters can be tremendously powerful and vice versa. What users care about is the expression yield obtained by a promoter, the length of it is quite secondary. I was misled by some of the figures, thinking that I was looking at stronger or weaker promoters, whereas what was displayed was this odd promoter strengths/bp thing. I suggest to remove these figures and stick to promoter strength."

Regarding ‘cumulative expression’:

When co-expressing multiple proteins, not only their ratios to each other, but also their total (cumulative) amounts summed together matter. Too excessive loads of heterologous proteins may overburden the cellular machinery of recombinant expression hosts. Hence, in addition to balancing the proteins relative to each other, their total (cumulative) expression strength needs to be adjusted. We thought at first about using the term “total” but chose “cumulative”, as several co-authors thought “total expression” to sound rather odd.

To avoid confusion, we have now added an explanation of the term to the very beginning of the manuscript.

Regarding ‘expression efficiency’:

We apologize that the explanation of how the expression efficiency was calculated was unclear. It was not our intention to mislead the reader. When writing the manuscript, we noticed that we repeatedly mentioned the short length of BDPs (which is advantageous for the pathway assemblies shown in Fig. 6d) while giving nonetheless high expression. But none of the figures initially supported this claim quantitatively. Plotting just the lengths in bp was missing the achievable expression strength, so we introduced ‘efficiency’ as a metric combing both properties to illustrate the data.

“I suggest to remove these figures and stick to promoter strength.”

The absolute promoter strengths (of both sides) were actually illustrated for the whole library as panel C of the old Fig. 3, as X/Y chart, which however cannot be sorted in any way and is hence somewhat confusing to interpret. We believe that the figure on the efficiency provides added value. It also represents a good summary on the groups of promoters generated, especially since the old Fig. 2 on the sBDP library has now been split into three figures (Figs. 2-4).

We have re-arranged the panels of the summarizing figure (old Figure 3, new Figure 5). The first panel (a) illustrates the absolute promoter strengths, panel (b) shows the ratios and panel (c) shows the efficiencies. We have also changed the term from ‘expression efficiency’ to ‘**relative** expression efficiency’. We have also added an explicit disclaimer, that ‘relative expression efficiency’ is a relative term introduced in this study and its caveats (*i.e.* values change for different fluorescence reporter proteins used and even with different fluorospectrometers for detection).

Response to Reviewer #2

Major comments

"1. In the manuscript title, but also in the abstract and the conclusions, the authors place special emphasis on the histone bidirectional promoters, and they also make a case for generalization of their findings based on the conservation of the histone BDP architecture between P. pastoris and other eukaryotes. Although several hybrid histone BDPs developed perform well in the fluorescent protein coupled reporter assay developed, neither the native or the hybrid histone BDPs prove to be so efficient when tested in "real-life" applications in the case studies used. In the taxadiene, Cyp2D6, or CalB case studies, optimal performance was achieved with native inducible promoter combinations, rather than inducible or constitutive histone-based promoters. Only in the carotenoid production case study have the histone hybrid promoters performed better, but the improvement over the simple linear gene arrangement under the inducible AOX1 promoter is only 2-fold, suggesting a very limited overall contribution of expression tuning in the performance of this system. Furthermore, the requirement of all case studies for inducible expression diminishes the usefulness of the non-hybrid histone BDPs. Although it is possible that the histone promoter-based hybrid variants will eventually be beneficial in certain cases, there is very little evidence so far for the authors to argue that the main benefits/novelty of their system stems from the architecture of the histone BDPs. The promoter resource developed here is a powerful tool in its own right and will have a strong impact in future synthetic biology applications, but the main advantage of the approach is the size and extent of the promoter collection and the construction of several synthetic BDPs with varied regulation. This is evidenced by the observation that a different combination of promoters was found to be optimal in each of the first three case studies, which makes a compelling case for testing such a large and diverse library of promoters as a standard strategy in similar synthetic biology applications. The authors should consider rephrasing this main claim to better reflect the findings."

Reviewer 1 has commented on similar issues (see specifically also the response to R1's comment #2 [second part]). We have hence removed 'histone promoters' from the title and revised the interpretation to limit the role of histone BDPs in the abstract and throughout the manuscript. The manuscript now highlights the usefulness of the histone BDPs as parts repository for promoter engineering.

By parts repository, we mean that not only the actual histone promoters and deletion variants were useful for expression fine-tuning, but that also the underlying sequence elements of the histone promotes such as the core promoters and regulatory regions were used to construct the bidirectionalized promoters (new Fig. 3a) and the bidirectional hybrid promoters (Fig. 4). These short core promoters are now illustrated as Fig. 2b and the accompanying discussion has been moved to the main text.

Regarding the performance of the wildtype histone promoters for dual gene expression and the carotenoid pathway, we have replied in detail to reviewer 1. So we will copy parts of this explanation already given here and amend it to cover all issues raised by R2:

The seemingly poor performance of the histone promoters is in part caused by the protein targets (TDS/GGPPS, CYP2D6/CPR). Constitutive expression of TDS/GGPPS and CYP2D6/CPR apparently causes too much cellular stress, hence the constitutive/growth associated histone promoters did not yield colonies after transformation. The same issue holds for the constructs bearing the suggested GAP or TEF promoters, as we obtained no transformants here either. Hence, this is not a limitation of the histone promoters specifically, but rather all constitutive promoters for these gene pairs.

For the other gene pair CalB/PDI and the carotene pathway, the histone promoters and GAP reference promoter did give expression. However, the derepressed and methanol-inducible *CAT1* promoter (on methanol) clearly outperformed any constitutive promoter (the histone promoters or P_{GAP}) for CalB/PDI. The best histone promoter (*HTA1-HTB1*) gave fair expression compared to P_{GAP}, reaching about 50% on glucose. Nonetheless, constitutive expression appears unfavorable compared to methanol induction for CalB/PDI expression.

"Only in the carotenoid production case study have the histone hybrid promoters performed better, but the improvement over the simple linear gene arrangement under the inducible AOX1 promoter is only 2-fold, suggesting a very limited overall contribution of expression tuning in the performance of this system."

Actually the methanol inducible 4xAOX1 design yielded about the same beta-carotene titers as the histone promoter based design. However, in this case the histone promoters provided a more than 10-fold improvement compared to the constitutive *GAP* promoter: The best bidirectional design based on histone promoters (C11) yielded 14.9-fold higher β-carotene titers than the monodirectional standard P_{GAP} design. Hence, the histone promoters performed in this case clearly better than GAP reference promoter. Methanol inducible BDPs (e.g. C2 and C7) yielded again higher yields (about two fold) than the histone BDPs. However, note that the histone strains did not need to be induced with methanol and were already harvested after 60 h cultivation, whereas the methanol induced construct had to be grown for additional 48 hours. Hence, regarding space/time yields the histone promoter provide an advantage and may be useful, especially in large scale production plants where induction with toxic and flammable methanol can represent a considerable safety risk. So specifically for pathway applications, the histone promoters did show promising results.

"2. The authors should also clarify in their abstract and discussion the extent by which the specific resource can be directly transferred to another chassis. Although the architecture of histone promoters is indeed conserved, in the case studies presented here the most successful promoter combinations turned out to be inducible. Since the methanol utilization capacity of P. pastoris is rather unique, for the hybrid histone BDPs-based promoters to be useful in another system, these must be redesigned and re-constructed using host-specific inducible promoters. Therefore, the generality of the approach lies in the overall strategies employed for the construction of the promoter library (and the lessons learned), rather than the ability to transfer a large collection of parts to other hosts."

We have rewritten the respective parts. Our key idea was that the engineering strategies (such as fusion promoters, deletions/truncations of histone promoters and using their core promoter for bidirectionalization) rather than the promoter sequences themselves are broadly applicable. Basically all of the 168 BDPs and will only function in *P. pastoris* and not in other organisms. So while we provide in this manuscript a library specific to *P. pastoris*, we also demonstrate the advantages of bidirectional expression strategies, meanwhile providing detailed descriptions of the underlying design processes to allow other researchers to engineer similar libraries in other organisms.

The text added to the conclusion section reads (extract):

"Generating similar BDP libraries in other organisms will require species specific engineering, especially for obtaining inducible promoters. Methanol inducible promoters are rather unique to *P. pastors* and other methylotrophic yeasts (Yurimoto et al., 2011), whereas other systems will require species specific

promoters such as galactose regulated promoters in *S. cerevisiae* (Weinhandl et al., 2014). In higher eukaryotes, where carbon source regulated promoters are scarce, inducible BDPs based on synthetic TFSs (Fux and Fussenegger, 2003) could be generated relying on strategies developed for MDPs (Khalil et al., 2012; Nevozhay et al., 2013)."

Minor comments:

"3. To facilitate evaluation of the results of the case studies, it would be useful to include in the figures 4a, b, and c the corresponding normalized expression (based on the fluorescent protein-based assay) of each of the BDPs together with the titer/activity (using a second axis and symbol on the same graph)."

We had actually had the same idea when we initially prepared the manuscript. But ultimately the figures then contained a huge amount of data and became fairly difficult to interpret (for example for taxadiene, there is just one bar representing the taxadiene titers and four more bars depicting the two promoter sides under repressed/derepressed and methanol induced conditions [see the illustration below]). So we had omitted the figures (as the main conclusion was, that the BDPs yields different results for each gene pair, and do not behave the same as with the FPs – which is basically also apparent when just comparing the three panels a,b,c from new Fig. 6 [former Fig. 4]).

In the revised version, the main text has also become longer and more complex. New Fig. 6 on the results still contains a fair amount of data already. Hence, we have included this new additional figure in the supplements as new supporting figure S9.

New figure S9:

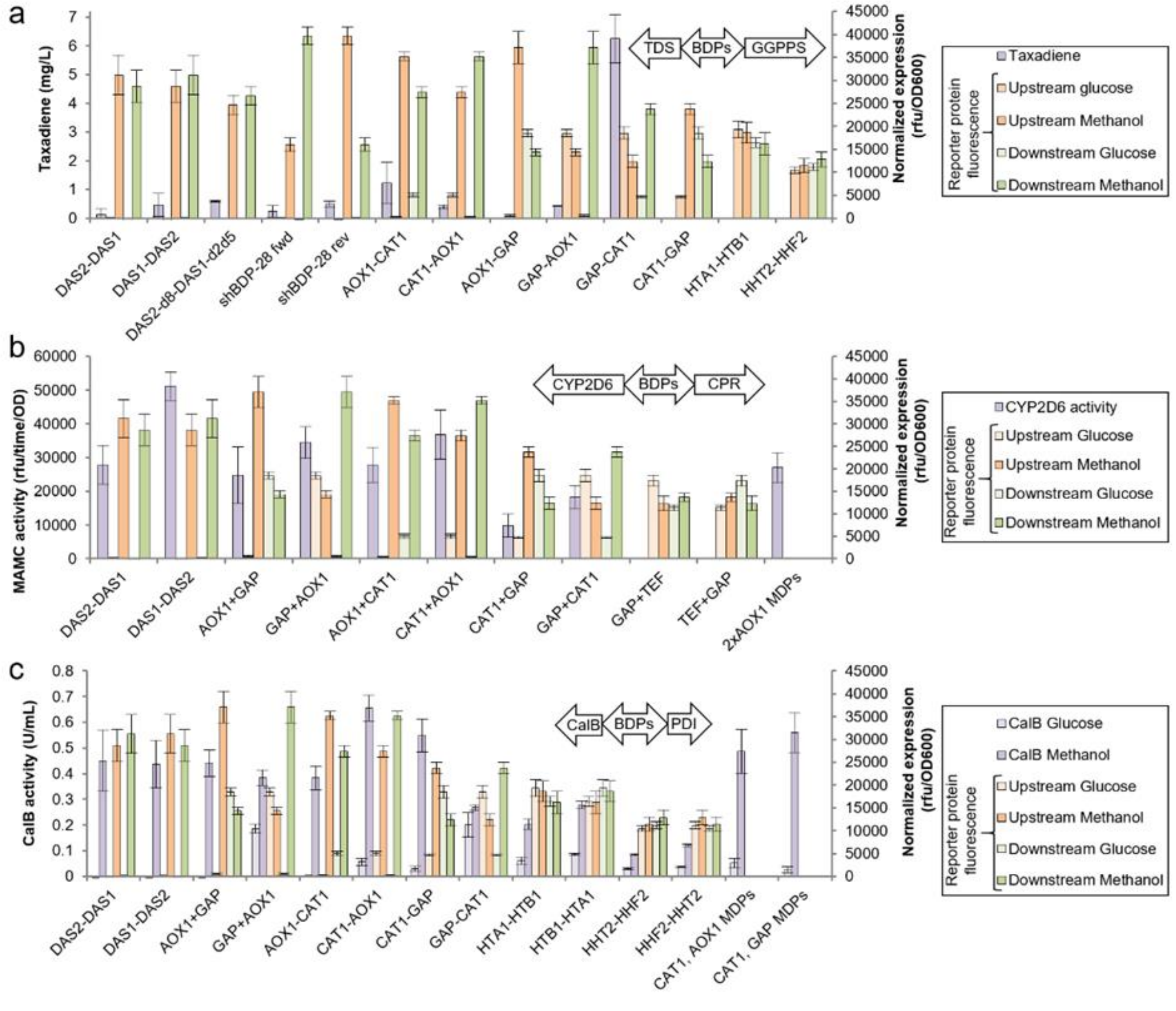

"4. The taxadiene titer obtained in the optimal BDP is indeed impressive, considering that the P. pastors strain used here is not engineered for isoprenoid production. However, the S. cerevisiae strain used for comparison in ref 37 is not a heavily engineered strain (line 203). An example of such a strain can be the one mentioned in (Westfall 2012, PNAS doi: 10.1073/pnas.1110740109). Please rephrase."

We have revised the text. We only meant that the *P. pastoris* strain was generated by transformation of a single plasmid, whereas in *S. cerevisiae* multiple plasmids were transformed (and genes knocked out). The revised text reads:

"Most strikingly, for taxadiene production, the worst strain produced only 0.1 mg/L, whereas the best strain (bearing a $P_{GAP+CAT1}$ fusion promoter) reached 6.2 mg/L, in range with ~~**heavily**~~ engineered *S. cerevisiae* strains **(8.7±0.85 mg/L)** (Engels et al., 2008)."

"5. In Figure 4d it is not so easy to read the identity of the different promoters/terminators."

We have increased the font size in all other revised figures, but here it is really not possible to make it bigger (if the scale of the elements to each other should be depicted correctly). We have also submitted a

vector graphics of this (and all other figures, at the end of the pdf file of the main manuscript), so eventually zooming in should not be limited. We have also added an explicit remark to supporting file S2 in the figure caption, listing the exact promoters/terminators used for each construct.

"6. Fig S1, and many other figures in the supplementary section are difficult to read. As there is not a tight size restriction in this section, the authors could try to help the reader by increasing the size of the diagrams."

We just noticed now that the conversion of S1 to the pdf in the supplements had not worked properly in the last version. We have now ensured that this figure (and others) were converted correctly, which has improved the readability (note that the old S1 has now been repositioned as S8).

"7. A lot of valuable information has been relegated to the supplementary section, presumably due to size considerations (one such example is in lines 411-416 in supplementary, but several instances exist). The authors should review the size of the manuscript and explore the possibility to bring some arguments/clarifications forward to main text."

Reviewer 1 and 3 have commented on the same issue, we have now reworked the paper and moved key results and the accompanying discussion from the supplements to the main manuscript. We have also moved lines 411-416 from the old SI to the main text.

"8. Line 492 in supplementary "Fold-differences….". Misplaced sentence?"

Thank you, yes that was an error. Data from this supporting figure has now been included in the main text as Fig. 2a and the extended discussion in the supplements was removed and shortened into the main text.

Response to Reviewer #3

Major comments

"1. There are two manuscripts in one here (The main text and the supporting information). The main text describes very directly (and fast) the main results of the work. For example, an entire figure (Figure 3) is presented and discussed in less then 5 lines (176-178 and 185-186). This makes difficult to fallow the work in light of the heave load Figures presented (specially Figure 2). In fact, there is a back and forth movement into the Figure 2. The authors should try to enhance a bit the presentation of the data and discussion of the results. In the case of Figure 2, this could be dived into two Figures, even by esthetic reasons. The letters in the figure are very small and only by zooming it I could follow which was each promoter constructed. I do not think it will be easy for a reader to follow it in a printed version of the paper. In the case of the supporting information, there is a extensive discussion on a number of control and additional experiments, with a lot of interpretations and expectations in some cases. This section could be simplified, with some of the critical discussion been incorporated to the main text. In fact, I have learned quite a bit more reading the SI then the main text."

Reviewer 1 and 2 had very similar comments on this issue. We have now reworked the paper and moved key results and the accompanying discussion from the supplements to the main manuscript (see the list of major changes at the beginning of this response). We have also shortened and removed extensive discussions from the supplements.

Regarding the old Fig. 2, we have now split this figure into three figures (Fig. 2 to Fig. 4), which should add clarity and avoid the back and forth movement mentioned by the reviewer. We have also increased the font sizes in these figures and they should be now easier to read.

"2. The experiments in S. pombe, S. cerevisiae and CHO cells are presented in the Material and Methods (lines 532 to 607) and presented very briefly in the conclusions. I do not see the point of this here. The flow bioinformatic singing-promoter engineering-pathway construction makes this manuscript worth already. Yet, if the authors think this is relevant to be present in the conclusion, then they should consider properly present and discuss the results in the previous section, with a proper Figure and conclusive data. Otherwise, removing this part would make the work less confusing. I particularly think the paper will be great even without this potential generalization."

Reviewer 1 made very similar comments and we have hence removed the data in *S. cerevisiae, S. pombe* and CHO cells.

Also, thank you for the enthusiastic words in the last sentence of this comment, it is very motivating and reassuring to receive also positive feedback like this.

"3. The authors present a number of muted version of the promoters, some by creating deletions others by fusing different parts. These variants present different behaviors in terms of indelibility and activity levels. Yet, there was not assessment of the single-cell behavior of the resulting promoters. The authors argued that a Flow Cytomiter with appropriate filters for dual analysis was not available at the institution. Yet, a typical 488 filter would allow then to investigate using GFP only whether the modulation of promoter activity by some critical modifications is the result from changes in the single-cell

behavior of the promoters (such as in Fig. 2b,c and d). This is very easy to do with the promoters already constructed and is critical to see if the new tools are indeed useful for pathway engineering. If the resulting promotors are switching toward a all-or-none behavior, that would mean that the genes of the engineered pathways would be expressed at different cell population. Perhaps that would explain why the strain expression taxadiene did not bit the S. cerevisiae strain. This kind of characterization is crucial for a manuscript of this magnitude."

We had actually performed such FACS experiments for monodirectional synthetic promoters to address similar issues raised by a reviewer in the past (Portela et al., 2017). These experiments were included as supporting figure and supporting text S6 in the previous publication (Portela et al., 2017) and are added here below (also parts of the following explanation are copied from this open access supporting text). In detail, we had compared FACS and plate reader results for natural promoters and synthetic variants (spanning a ~10-fold expression range). In general the FACS and fluorescent plate reader readings were in excellent agreement (R^2=0.96, see figure below).

Regarding the population effects (all-or-none behavior) mentioned by reviewer 3, we did notice bimodal distributions for methanol inducible promoters (appearing to show two distinct populations after induction, see figure below). These populations may be caused by the experimental setup of the methanol induction: cells are at first grown on glucose and then induced with methanol. The different cell populations upon methanol induction may be attributable to 'older' cells, having been grown on glucose and subsequently induced, and 'new' cells emerging from cell divisions after methanol induction and hence only grown under these conditions. These two peaks occurred for all promoters tested (the synthetic ones and also the control of an unmodified wildtype promoter). Hence, these differences appear to be an inherent trait of the methanol induced yeast cells and not caused by the synthetic promoter design.

Also many of the bidirectional promoters reported in this study were generated by a related strategies of fusing core promoters to CRMs (Fig. 4) or to entire MDPs for bidirectionalization (Fig. 3a). Hence we expect designs based on methanol inducible CRMs, to show similar bimodal population distributions as natural promoters and monodirectional synthetic promoters. When applying these synthetic bidirectional promoters for pathway fine-tuning applications (carotenoid), they actually outperformed the monodirectional P_{AOX1} system (with known population distributions (Portela et al., 2017)) and matched previously reported monodirectional designs (Vogl et al., 2016). Thus, we conclude population effects of BDPs to be in the same range or even less pressing than for P_{AOX1} and hence not to be a key bottleneck associated with the bidirectional design.

Regarding the statement that population effects "[…] *would explain why the strain expression taxadiene did not beat the S. cerevisiae strain."*:

We think that the key reason for our strains not beating and being 'only' at par with *S. cerevisiae* designs lies in the degree of additional strain optimizations performed. In such works in *S. cerevisiae* (Engels et al., 2008) or *E. coli* (Ajikumar et al., 2010), the upstream mevalonate pathway (in the *E. coli* non-mevalonate/MEP/DOXP pathway) was optimized to provide sufficient precursors. Therefore multiple plasmids were transformed and genes knocked out. In our work, titers matching optimized *S. cerevisiae* strains were obtained by transforming a single plasmid (Note that reviewer 2 also acknowledged this "*The taxadiene titer obtained in the optimal BDP is indeed impressive, considering that the P. pastors strain used here is not engineered for isoprenoid production.*"). We have added an extended discussion on this to the main manuscript, as also reviewer 2 was commenting on this.

Also related to population effects for the actual production (e.g. taxadiene, carotenoide) strains, this would be nearly impossible to investigate, as we ultimately cannot measure single cell levels of taxadiene. But from the data available the taxadiene expressing strains did appear to behave uniformly. For example constitutive expression of the GGPPS gene was lethal to basically all transformants. If only some cells expressed the genes and others did not, lower ODs and a slower growth would have been apparent. However, all colonies behaved the same suggesting that there were no effects only in a subset of the cells.

Reproduced figure S6 from (Portela et al., 2017)

(A) The plate reader based fluorescence measurements are in excellent agreement with flow cytometry measurements:

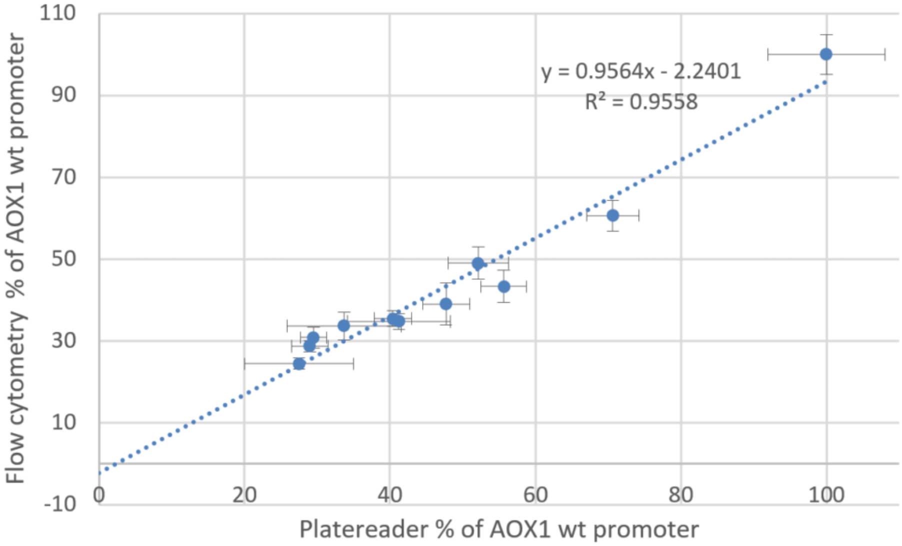

(B) FACS histograms of cell counts and respective measured fluorescence (log scale) of three representative biological replicates measurements (30000 events each) [Note the different scaling of the y-axis for each plot]:

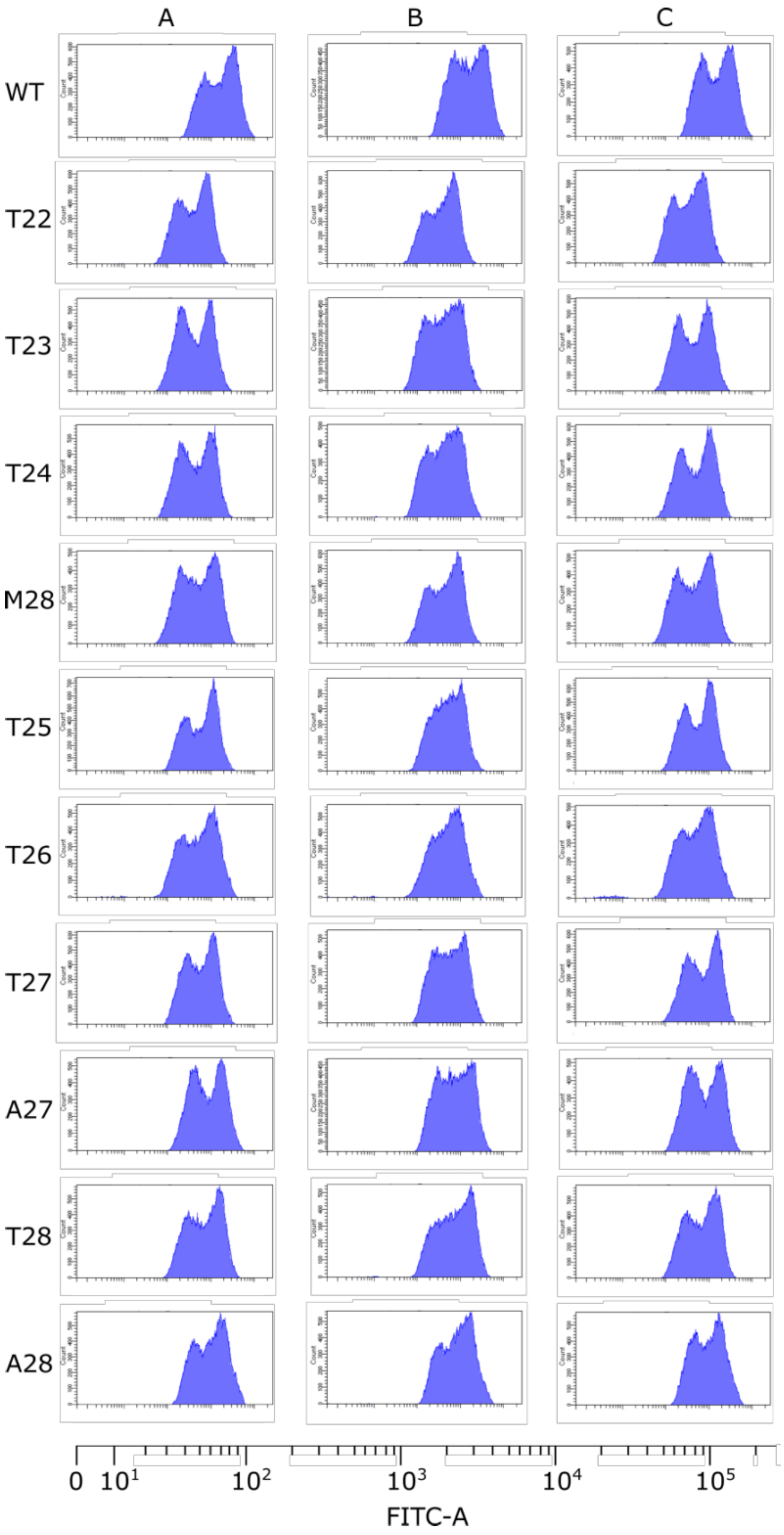

A
B
C
WT
T22
T23
T24
M28
T25
T26
T27
A27
T28
A28
0
10^1
10^2
10^3
10^4
10^5
FITC-A

"4. The authors used eGFP and dTomato to quantify promoter activity. They presented int he SI a investigation on the differences in signal produced by the two reporters (page 9). Yet, this information is very relevant and should be clearly presented in the main text. In other words, are the RFU/OD600 data presented along the manuscript corrected using the data provided in page 9 of SI?"

Yes, in all figures on the promoter characterizations in the main manuscript (as well as the supplements) the normalization factor to make eGfp and dTom fluorescence comparable was used. We have now explicitly mentioned this in the main text and in every Figure caption. We would prefer to keep the normalization figure in the supplements as it is mostly a technical control while not providing key information on the bidirectional promoters themselves (and the main manuscript is fairly dense already).

"5. Related to the previous point, the used of GFP, RFP and OD600 quantification can lead to some interference in signal as demonstrated by Hecht et al 2016 (https://pubs.acs.org/doi/abs/10.1021/acssynbio.6b00072). The same approach is used here. Have the authors checked these potential interferences here? This should be clearly demonstrated and discussed."

Thank you for bringing this paper to our attention. We had not been aware of this work in *E. coli* and had initially not specifically investigated such effects of high dTom fluorescence on OD600 measurements. However, if there had been a major bias, our normalization experiments, where eGfp and dTom fluorescence were extensively compared, would have revealed it. We had also specifically looked at cell growth of dTom expressing cells in comparison with the wildtype strain in previous work (Vogl et al., 2018b) [supporting figure S6 in that paper] and had not noticed an impact of dTom expression. It is worth noting that after correction factor application, the histone promoters give nearly identical expression on both sides. Additionally, the *AOX1* promoter tested in both directions (for example shown in Fig. 4, control at the very bottom bottom) gives nearly identical values if tested with eGFP or dTOM. Thus, if there were an interference with OD600 measurements as noted by Hecht et al., it would be evident for these controls.

However why did we not experience the issues reported by Hecht et al.?

We assume that there are two main reasons:

1.) We performed the experiments in the yeast *P. pastoris* and not in the bacterium *E. coli*. It appears plausible that the ratios between fluorescent proteins inside of the cells and the biomass is different between bacteria and yeasts. *P. pastori*s is known for growth to exceptionally high cell densities (up to 500 g/l cell wet weight in bioreactors and even in deep well plates ODs of ~20 to 30 can be reached) whereas the fluorescent protein production does not necessarily increase the same way. Hence a 10x denser *P. pastoris* culture might actually contain relatively less protein than *E. coli* cells. Hence there could be simply relatively less fluorescent protein interfering with the biomass measurement.

2) We have used a different red fluorescent protein variant than Hecht et al. with notable differences in excitation/emission wavelengths: We have used dTomato (excitation/emission wavelength: 554/581 nm (Shaner et al., 2004)) whereas Hecht et al. have used mRFP1 (excitation/emission wavelength: 584/607 nm; original mRFP1 reference: (Campbell et al., 2002)). So the em/ex peaks are shifted by 30/26 nm respectively, placing mRFP1 used by Hecht et al. notably closer to 600 nm used for OD600 measurements. Hence it appears plausible that the issue noted by Hecht et al. does not occur for all RFP variants.

We have added a discussion on these issues to S4 to make other researchers (who possibly want to perform similar experiments) aware of this issue.

“6. A strong weight is placed on Figure 2. Since glycerol, glucose and methanol are used to induce of repress promoter activities, it should be better presented in which cases the promoters are expected to be constitutive and in which a induction/repression is expected to occur.”

That is a very good point, but practically we are not sure how to do that the best way. We have now split the old Fig. 2 in three new figures (Fig. 2 to 4). Thereby there is somewhat more space available, however we would have to add a column to the figures labeled “Regulation” or more accurately “Expected regulation of MDP” and provide the expected outcome for each of the two bidirectional sides. But as the figures are still quite dense, this would make them more difficult to read.

We have now added the information on the regulation of the promoters to the end of the figure captions. Most relevantly, for (the new) Fig. 3 (old Fig. 2c,d,e) this reads:

“The bidirectionalized and fusion BDPs maintained the regulatory modes of the respective MDPs (Vogl et al., 2016; Vogl and Glieder, 2013): methanol inducible and tightly glucose/glycerol repressed (P_{AOX1}, P_{PMP20}, $P_{DAS1/2}$ [and deletion variants thereof], P_{FBA2}, P_{TAL2}, P_{AOX2}), derepressed and methanol inducible (P_{CAT1}, P_{FLD1}, P_{FDH1}) and constitutive (P_{GAP}, P_{TEF1}, P_{ADH2}, $P_{HTX1\ [HTA1\text{-}HTB1]}$).”

This is the best solution we could find without making the figures more overloaded.

“7. The way the new P. pastoris strains performs compared to previous attempts should be better presented (with the actual values), such as in line 202 and 227.”

We have now added the actual values to the main text (old line 202 (taxadiene): 8.7±0.85 mg/L / old line 227(carotene): 5.2±0.26 mg/g CDW [cell dry weight]

“5. How easy would be for the public to reuse some of the new parts created here? How some would reproduce the experiments using the engineered promoters. The tables provided the primers used for construction of the promoters, but the authors should consider to provide some annotated sequences that could be directly synthetized and used, in cases where it does not conflicts with the existing patents. Otherwise, it will be not easy to fallow those instruction and reproduce the experiments.”

Reviewer 1 has commented on a similar issue. We have now added a table with a set of 12 (if tested in both orientations) BDPs representing key features of our library (and included also annotated sequence files for these promoters to make them easily accessible for other researchers). The table is included in the main manuscript (Table 1), the sequence files as S3 (in GenBank format). Reviewer 1 had a point stating that “*Few readers will want to start utilizing 168 promoters of which many are rather similar in behaviour. The main utility of a paper like this is if it can condense all of the obtained data to clear instructions on a minimized set of tools that readers can then confidently use, knowing that they will miss very little opportunities for further enhancement in their designs.”* We think that these annotated sequences will be sufficient for most readers. If anyone is interested in additional constructs (and does not want to look up the primers in the supplements), we can communicate all sequences to anyone interested. There are no IP restrictions on sharing these sequences for research purposes, only their commercial application is limited by the patents.

Minor comments

“1. As I said before, the SI material is quite dense. Please revise it for potential errors in information (such as “Pp hybrid promoter”in line 226 vs “Pp hybrid BDPs in the Excel table). There are different style in the text, such as eGFP vs. eGfp.“

As mentioned above, we have shortened the SI and thereby also checked for errors, including the ones mentioned by the reviewer. About the different styles of writing eGFP (this also applies to the second FP, dTomato): We tried to apply standard yeast nomenclature, so when referring to the gene we write it as *eGFP* (upper case italics, as for example also for *AOX1*). If we refer to the protein we write eGfp (as Aox1p [as GFP means green fluorescence protein, we do not use the abbreviation eGfpp]. In the last version especially in the supplements, we did not uniformly follow this naming, we have now again double checked it [in vector and primer names it is just spelled as ‘eGFP’, we do not use italics there].

“2. Page 9. I do not see how FRET fits here, since there is not reason for the two proteins be in close proximity for this to occur. No need for this speculation.”

We have removed this sentence.

“3. Page 26. What do the authors understand for “more energy”? Is it reducing power? ATP? There are some good literature on resource allocation and metabolic burden that can be used here.”

We have clarified these statements and added a reference on metabolic burden. The new text reads: “Producing two FPs at the same time may require more resources in the form of amino acids and capacities for protein synthesis/folding from the cell and represent a greater metabolic burden (Wu et al., 2016) than expressing a single FP.”

“4. Page 26. What is CYP (line 775)?”

Assuming this is referring to the SI (the main text had only 20 pages), it’s rather page 28 and lines 725ff? CYP was supposed to be an abbreviation for Cytochrome P450, but in an effort to shorten the manuscript we have removed this extended discussion (and moved essential parts to the main text, we have now checked, there the abbreviations are always given).

References for response to reviewers

Ajikumar, P.K., Xiao, W.-H., Tyo, K.E.J., Wang, Y., Simeon, F., Leonard, E., Mucha, O., Phon, T.H., Pfeifer, B., Stephanopoulos, G., 2010. Isoprenoid pathway optimization for Taxol precursor overproduction in Escherichia coli. Science 330, 70–4. doi:10.1126/science.1191652

Amendola, M., Venneri, M.A., Biffi, A., Vigna, E., Naldini, L., 2005. Coordinate dual-gene transgenesis by lentiviral vectors carrying synthetic bidirectional promoters. Nat. Biotechnol. 23, 108–16. doi:10.1038/nbt1049

Aw, R., Polizzi, K.M., 2013. Can too many copies spoil the broth? Microb. Cell Fact. 12, 128. doi:10.1186/1475-2859-12-128

Campbell, R.E., Tour, O., Palmer, A.E., Steinbach, P.A., Baird, G.S., Zacharias, D.A., Tsien, R.Y., 2002. A monomeric red fluorescent protein. Proc. Natl. Acad. Sci. U. S. A. 99, 7877–82. doi:10.1073/pnas.082243699

Da Silva, N.A., Srikrishnan, S., 2012. Introduction and expression of genes for metabolic engineering applications in Saccharomyces cerevisiae. FEMS Yeast Res. 12, 197–214. doi:10.1111/j.1567-1364.2011.00769.x

Engels, B., Dahm, P., Jennewein, S., 2008. Metabolic engineering of taxadiene biosynthesis in yeast as a first step towards Taxol (Paclitaxel) production. Metab. Eng. 10, 201–6. doi:10.1016/j.ymben.2008.03.001

Fux, C., Fussenegger, M., 2003. Bidirectional expression units enable streptogramin-adjustable gene expression in mammalian cells. Biotechnol. Bioeng. 83, 618–25. doi:10.1002/bit.10713

Khalil, A.S., Lu, T.K., Bashor, C.J., Ramirez, C.L., Pyenson, N.C., Joung, J.K., Collins, J.J., 2012. A synthetic biology framework for programming eukaryotic transcription functions. Cell 150, 647–58. doi:10.1016/j.cell.2012.05.045

Li, A., Liu, Z., Li, Q., Yu, L., Wang, D., Deng, X., 2008. Construction and characterization of bidirectional expression vectors in Saccharomyces cerevisiae. FEMS Yeast Res. 8, 6–9. doi:10.1111/j.1567-1364.2007.00335.x

Lin-Cereghino, J., Wong, W.W., Xiong, S., Giang, W., Luong, L.T., Vu, J., Johnson, S.D., Lin-Cereghino, G.P., 2005. Condensed protocol for competent cell preparation and transformation of the methylotrophic yeast Pichia pastoris. Biotechniques 38, 44, 46, 48.

Miller, C.A., Martinat, M.A., Hyman, L.E., 1998. Assessment of aryl hydrocarbon receptor complex interactions using pBEVY plasmids: expressionvectors with bi-directional promoters for use in Saccharomyces cerevisiae. Nucleic Acids Res. 26, 3577–83.

Näätsaari, L., Mistlberger, B., Ruth, C., Hajek, T., Hartner, F.S., Glieder, A., 2012. Deletion of the Pichia pastoris KU70 homologue facilitates platform strain generation for gene expression and synthetic biology. PLoS One 7, e39720. doi:10.1371/journal.pone.0039720

Nevozhay, D., Zal, T., Balázsi, G., 2013. Transferring a synthetic gene circuit from yeast to mammalian cells. Nat. Commun. 4, 1451. doi:10.1038/ncomms2471

Öztürk, S., Ergün, B.G., Çalık, P., 2017. Double promoter expression systems for recombinant protein production by industrial microorganisms. Appl. Microbiol. Biotechnol. 101, 7459–7475. doi:10.1007/s00253-017-8487-y

Partow, S., Siewers, V., Bjørn, S., Nielsen, J., Maury, J., 2010. Characterization of different promoters for designing a new expression vector in Saccharomyces cerevisiae. Yeast 27, 955–64. doi:10.1002/yea.1806

Portela, R.M.C., Vogl, T., Ebner, K., Oliveira, R., Glieder, A., 2018. Pichia pastoris Alcohol Oxidase 1 (AOX1) Core Promoter Engineering by High Resolution Systematic Mutagenesis. Biotechnol. J. 13, e1700340. doi:10.1002/biot.201700340

Portela, R.M.C., Vogl, T., Kniely, C., Fischer, J.E., Oliveira, R., Glieder, A., 2017. Synthetic Core Promoters as Universal Parts for Fine-Tuning Expression in Different Yeast Species. ACS Synth. Biol. 6, 471–484. doi:10.1021/acssynbio.6b00178

Schwarzhans, J.-P., Wibberg, D., Winkler, A., Luttermann, T., Kalinowski, J., Friehs, K., 2016a. Integration event induced changes in recombinant protein productivity in Pichia pastoris discovered by whole genome sequencing and derived vector optimization. Microb. Cell Fact. 15, 84. doi:10.1186/s12934-016-0486-7

Schwarzhans, J.-P., Wibberg, D., Winkler, A., Luttermann, T., Kalinowski, J., Friehs, K., 2016b. Non-canonical integration events in *Pichia pastoris* encountered during standard transformation analysed with genome sequencing. Sci. Rep. 6, 38952. doi:10.1038/srep38952

Shaner, N.C., Campbell, R.E., Steinbach, P.A., Giepmans, B.N.G., Palmer, A.E., Tsien, R.Y., 2004. Improved monomeric red, orange and yellow fluorescent proteins derived from Discosoma sp. red fluorescent protein. Nat. Biotechnol. 22, 1567–72. doi:10.1038/nbt1037

Vickers, C.E., Bydder, S.F., Zhou, Y., Nielsen, L.K., 2013. Dual gene expression cassette vectors with antibiotic selection markers for engineering in Saccharomyces cerevisiae. Microb. Cell Fact. 12, 96. doi:10.1186/1475-2859-12-96

Vogl, T., Gebbie, L., Palfreyman, R.W., Speight, R., 2018a. Effect of Plasmid Design and Type of Integration Event on Recombinant Protein Expression in Pichia pastoris. Appl. Environ. Microbiol. 84, AEM.02712-17. doi:10.1128/AEM.02712-17

Vogl, T., Glieder, A., 2013. Regulation of Pichia pastoris promoters and its consequences for protein production. N. Biotechnol. 30, 385–404. doi:10.1016/j.nbt.2012.11.010

Vogl, T., Ruth, C., Pitzer, J., Kickenweiz, T., Glieder, A., 2014. Synthetic Core Promoters for Pichia pastoris. ACS Synth. Biol. 3, 188–91. doi:10.1021/sb400091p

Vogl, T., Sturmberger, L., Fauland, P.C., Hyden, P., Fischer, J.E., Schmid, C., Thallinger, G.G., Geier, M., Glieder, A., 2018b. Methanol independent induction in Pichia pastoris by simple derepressed overexpression of single transcription factors. Biotechnol. Bioeng. 115, 1037–1050. doi:10.1002/bit.26529

Vogl, T., Sturmberger, L., Kickenweiz, T., Wasmayer, R., Schmid, C., Hatzl, A.-M., Gerstmann, M.A., Pitzer, J., Wagner, M., Thallinger, G.G., Geier, M., Glieder, A., 2016. A Toolbox of Diverse Promoters Related to Methanol Utilization: Functionally Verified Parts for Heterologous Pathway Expression in Pichia pastoris. ACS Synth. Biol. 5, 172–86. doi:10.1021/acssynbio.5b00199

Weinhandl, K., Winkler, M., Glieder, A., Camattari, A., 2014. Carbon source dependent promoters in yeasts. Microb. Cell Fact. 13, 5. doi:10.1186/1475-2859-13-5

Weninger, A., Fischer, J.E., Raschmanová, H., Kniely, C., Vogl, T., Glieder, A., 2018. Expanding the CRISPR/Cas9 toolkit for Pichia pastoris with efficient donor integration and alternative resistance

markers. J. Cell. Biochem. 119, 3183–3198. doi:10.1002/jcb.26474

Weninger, A., Hatzl, A.-M., Schmid, C., Vogl, T., Glieder, A., 2016. Combinatorial optimization of CRISPR/Cas9 expression enables precision genome engineering in the methylotrophic yeast Pichia pastoris. J. Biotechnol. 235, 139–49. doi:10.1016/j.jbiotec.2016.03.027

Wu, G., Yan, Q., Jones, J.A., Tang, Y.J., Fong, S.S., Koffas, M.A.G., 2016. Metabolic Burden: Cornerstones in Synthetic Biology and Metabolic Engineering Applications. Trends Biotechnol. 34, 652–664. doi:10.1016/j.tibtech.2016.02.010

Xie, M., He, Y., Gan, S., 2001. Bidirectionalization of polar promoters in plants. Nat. Biotechnol. 19, 677–9. doi:10.1038/90296

Yan, C., Zhang, D., Raygoza Garay, J.A., Mwangi, M.M., Bai, L., 2015. Decoupling of divergent gene regulation by sequence-specific DNA binding factors. Nucleic Acids Res. 1–12. doi:10.1093/nar/gkv618

Yang, S., Sleight, S.C., Sauro, H.M., 2012. Rationally designed bidirectional promoter improves the evolutionary stability of synthetic genetic circuits. Nucleic Acids Res. 1–7. doi:10.1093/nar/gks972

Yurimoto, H., Oku, M., Sakai, Y., 2011. Yeast methylotrophy: metabolism, gene regulation and peroxisome homeostasis. Int. J. Microbiol. 2011, 101298. doi:10.1155/2011/101298

REVIEWERS' COMMENTS:

Reviewer #1 (Remarks to the Author):

The authors have diligently replied to the comments that I had made, and provided the data of the control experiments using monodirectional promoters. These indeed indicate that similar results are obtainable using these monodirectional promoters, and the authors make the point that the bidirectional strategy can be used to screen for which promoter combinations are most suitable, to then implement this in production strains with the constituent monodirectional promoters. That is possible but the reliability of such approach is put in doubt to some extent by the experiments that show that in bidirectional promoters, the two promoters can influence eachother. It leaves one wondering whether starting from monodirectional promoters, using a combinatorial cloning system (for example, Golden Gate cloning based) would not be the more reliable approach). Nevertheless, this does not detract from the utility of bidirectional promoters should one prefer to use those.

I still take issue with the term 'expression efficiency' as meaning 'expression strength per bp of promoter length'. 'Efficiency' has the clear meaning in our community of 'level of expression' and will be understood as such by most readers. I insist to remove that term from the paper altogether and instead use 'expression strength per bp of promoter length'. It is longer indeed, but will avoid many misunderstandings.

The paper has been much focused by removing the less elaborate parts on other expression systems, as multiple reviewers incl. myself suggested. I'm sure those data will find their way in other manuscripts.
I would think that it is appropriate to add the species name 'Komagataella phaffii' to the title of the final manuscript, indicating that all of the work is in this organism.

It is good to see a limited-options table now of which bidirectional promoter combinations the authors think the user of this now assembled know-how should test for new applications. Indeed, most of these combinations are between very classical Pichia promoters, as we noted before.

Overall, with the above suggested small changes I think the paper is now in a shape in which the conclusions are supported by the data and in which the careful reader will find sufficiently clear information to decide as to whether the described promoter systems would be useful for their own research projects.

Reviewer #2 (Remarks to the Author):

The authors have made an extensive effort to rework the manuscript to address the comments made by the reviewers. This has improved readability immensely. The aims and main findings are now clear to the reader.

The authors have also responded positively to the suggestion for changing the title and the focus, and this has helped in strengthening the main argument behind the paper.

Overall, I am satisfied with the response of the authors in all the major and minor comments I have raised and I have no further critique to make at this stage.

Reviewer #3 (Remarks to the Author):

The authors substantially improved their manuscript and have been able to address the concerns raised in the first review. More specifically, the manuscript is now much easy to follow after the division of the figures. Also, six promoter variants are provided as supplementary material so other

groups can explore their promoters further. I am quite satisfied with this current version of their work.

Response to reviewers’ comments

R1

“The authors have diligently replied to the comments that I had made, and provided the data of the control experiments using monodirectional promoters. These indeed indicate that similar results are obtainable using these monodirectional promoters, and the authors make the point that the bidirectional strategy can be used to screen for which promoter combinations are most suitable, to then implement this in production strains with the constituent monodirectional promoters. That is possible but the reliability of such approach is put in doubt to some extent by the experiments that show that in bidirectional promoters, the two promoters can influence eachother. It leaves one wondering whether starting from monodirectional promoters, using a combinatorial cloning system (for example, Golden Gate cloning based) would not be the more reliable approach). Nevertheless, this does not detract from the utility of bidirectional promoters should one prefer to use those.”

We agree with the reviewer, for sure not all BDPs could be easily reconstituted with MDPs. It will not be possible to mimic some synergistic effects of BDPs with MDPs. We have mentioned this example rather as theoretical possibility. In the vast majority of cases, it will not be necessary to change from a successful BDP system to MDPs. Few researchers will actually want to take the effort to recreate a successful BDP strain with MDPs. Also, for screenings, the BDP strategies appears advantageous over the mentioned Golden Gate approach, as the bidirectional cloning via restriction site free TA-cloning or Gibson assembly is seamless (*i.e.* no additional restriction sites are added that could interfere with transcription/translation initiation at the 5’end of the gene or termination at the 3’end).

“I still take issue with the term 'expression efficiency' as meaning 'expression strength per bp of promoter length'. 'Efficiency' has the clear meaning in our community of 'level of expression' and will be understood as such by most readers. I insist to remove that term from the paper altogether and instead use 'expression strength per bp of promoter length'. It is longer indeed, but will avoid many misunderstandings.”

We have removed the term 'expression efficiency' and replaced it everywhere with 'expression strength per bp of promoter length'.

“The paper has been much focused by removing the less elaborate parts on other expression systems, as multiple reviewers incl. myself suggested. I'm sure those data will find their way in other manuscripts. I would think that it is appropriate to add the species name 'Komagataella phaffii' to the title of the final manuscript, indicating that all of the work is in this organism.”

We do not have a strong preference for either changing the title or leaving it the same. It is true that the final version of the manuscript contains only promoter designs for *K. phaffii.* On the other hand, major parts of the work such as the cloning scheme, promoter screening/design strategies and the whole concept of using a library of bidirectional promoters to optimize gene coexpression appear universally applicable and will also be relevant to researchers working with other biological systems.
