# Supplementary material for: Engineered bidirectional promoters enable rapid multi-gene co-expression optimization
Source: Nat Commun. 2018 Sep 4;9:3589. doi: 10.1038/s41467-018-05915-w (PMC6123417; doi:10.1038/s41467-018-05915-w)
Supplement: Supplementary file 1 — Peer Review File [file 41467_2018_5915_MOESM1_ESM.pdf]

Supplementary information

Engineered bidirectional promoters enable rapid multi-gene co-expression optimization

Vogl *et al*.

Index

1. Supplementary figures

2. Supplementary tables

3. Supplementary notes

4. Supplementary Data - see separate Excel and RAR files

5. Supplementary references

Supplementary figures

Supplementary Figure 1 (Reporter proteins normalization)

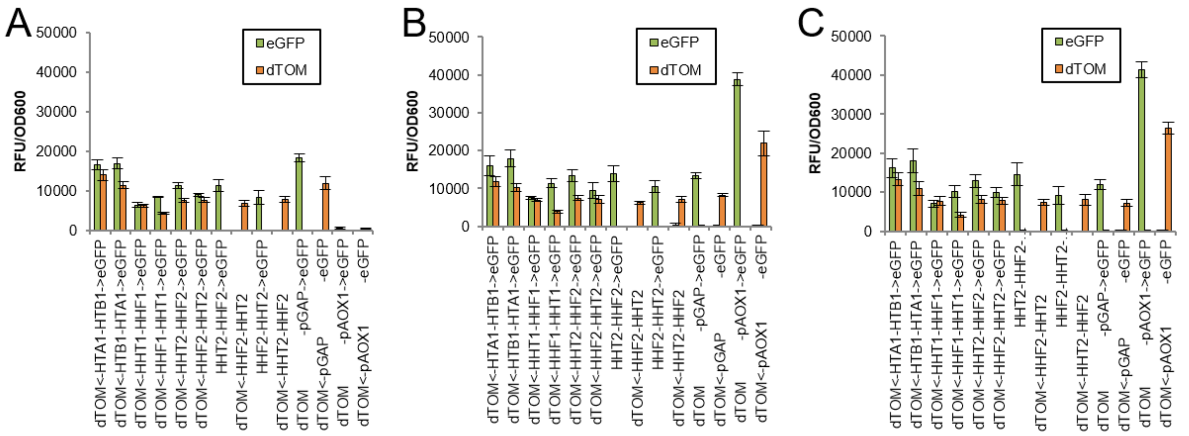

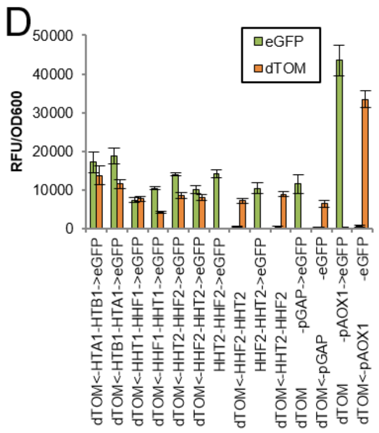

E

	Ratio eGFP/dTOM			
Values compared	60h glucose	24h MeOH	48h MeOH	72h MeOH
pHTB1-eGFP/pHTB1-dTOM	1.451	1.579	1.468	1.492
pHTA1-eGFP/pHTA1-dTOM	1.214	1.522	1.350	1.364
pHHF1-eGFP/pHHF1-dTOM	1.491	1.944	1.709	1.790
pHHT1-eGFP/pHHT1-dTOM	1.362	1.642	1.328	1.361
pHHF2-eGFP/pHHF2-mTOM	1.481	1.902	1.643	1.735
pHHT2-eGFP/pHHT2-dTOM	1.178	1.273	1.213	1.192
pHHF2-eGFP/pHHF2-dTOM (only one fluorescent protein)	1.665	2.236	1.993	1.985
pHHT2-eGFP/pHHT2-dTOM (only one fluorescent protein)	1.059	1.456	1.148	1.185
pGAP-eGFP/pGAP-dTOM	1.538	1.626	1.657	1.805
pAOX1-eGFP/pAOX1-dTOM	-	1.772	1.572	1.301
Mean value	1.38	1.70	1.51	1.52
Standard deviation	0.20	0.28	0.26	0.29

Supplementary Figure 1: Normalization of the two fluorescent reporter proteins used for characterization of the BDPs in *P. pastoris*. Bidirectional histone promoters and the monodirectional *GAP* and *AOX1* promoters were cloned between the two reporter genes *eGFP* (enhanced Gfp) and *dTOM* (dTomato, an enhanced red fluorescent protein variant [1]). Reporter gene fluorescence was measured after **(a)** 60 h growth on glucose and subsequently **(b)** after 24 h, **(c)** 48 h and **(d)** 72 h methanol induction. For each construct and time point a normalization factor was calculated **(e)** by dividing the indicated eGfp value by the dTom values. In panels a to d mean values and standard deviations of reporter protein fluorescence measurements of biological quadruplicates grown on the respective carbon sources are shown. See Supplementary Note 2 for details on the experimental design.

Supplementary Figure 2 (Natural $P_{DAS1/DAS2}$, deletion variants of P_{DAS1} and P_{DAS2} + P_{AOX1} regulatory elements)

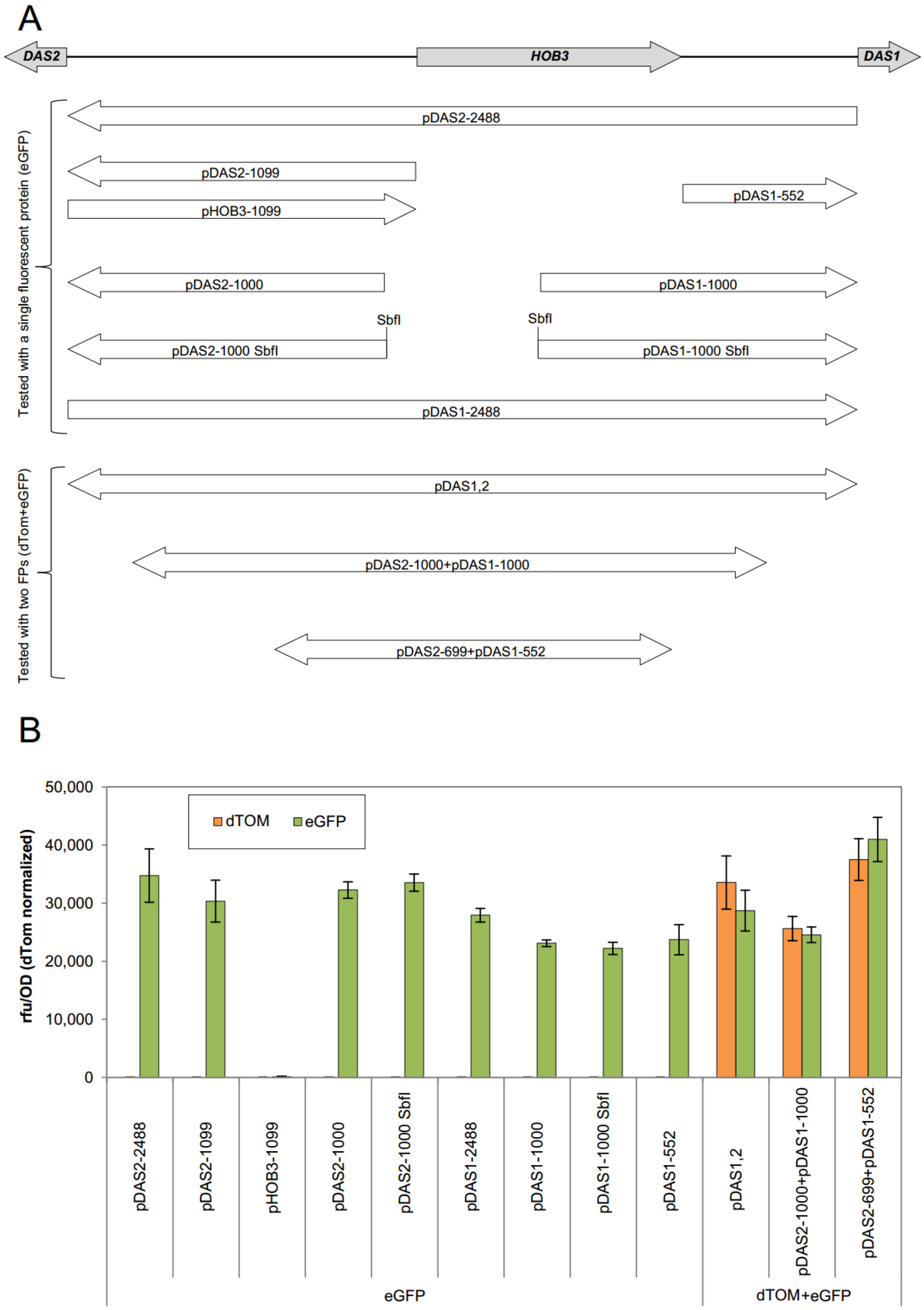

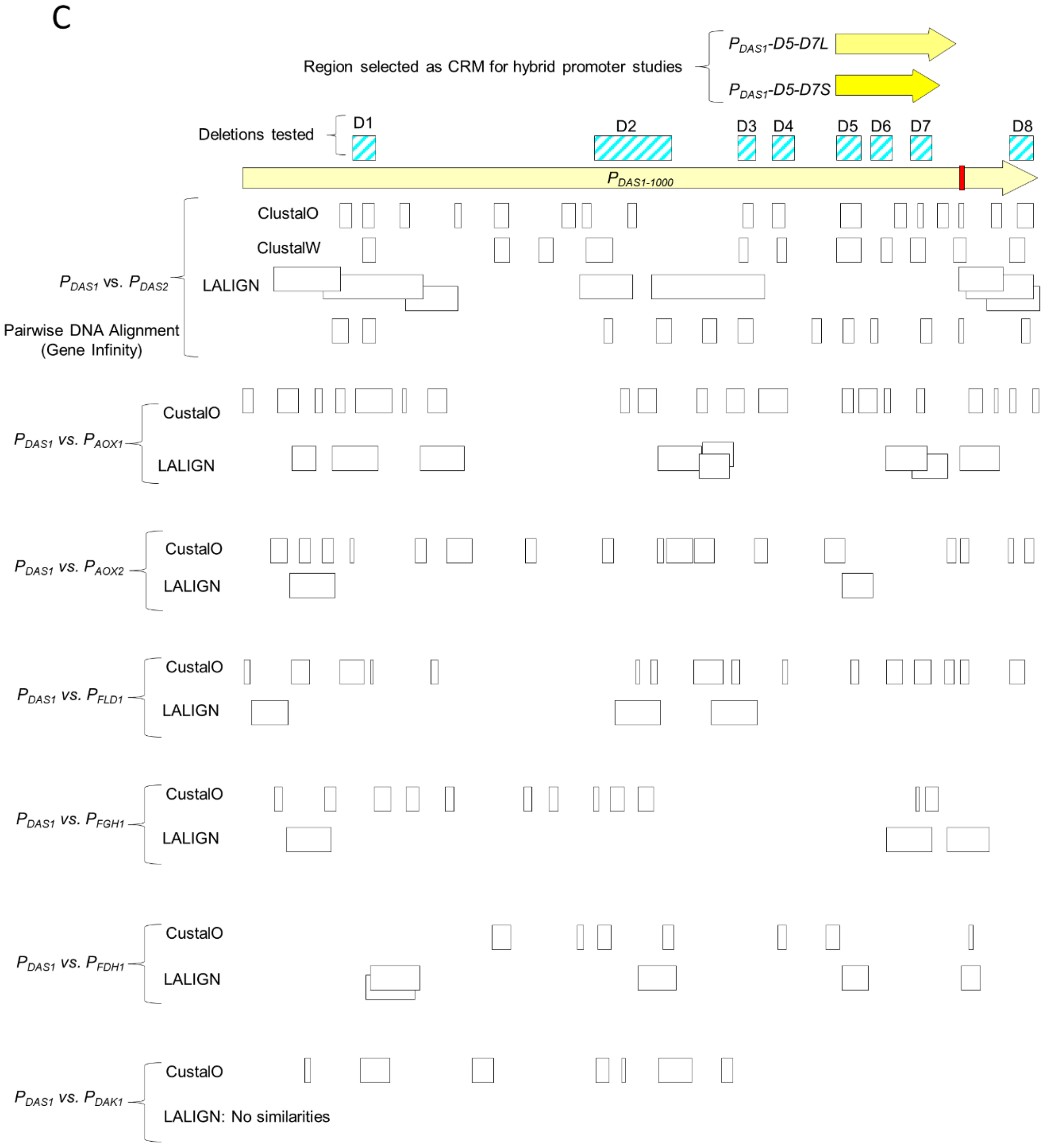

C
Region selected as CRM for hybrid promoter studies
P_{DAS1}-D5-D7L
P_{DAS1}-D5-D7S
Deletions tested
D1
D2
D3
D4
D5
D6
D7
D8
$P_{DAS1-1000}$
ClustalO
ClustalW
P_{DAS1} vs. P_{DAS2}
LALIGN
Pairwise DNA Alignment
(Gene Infinity)
P_{DAS1} vs. P_{AOX1}
CustalO
LALIGN
P_{DAS1} vs. P_{AOX2}
CustalO
LALIGN
P_{DAS1} vs. P_{FLD1}
CustalO
LALIGN
P_{DAS1} vs. P_{FGH1}
CustalO
LALIGN
P_{DAS1} vs. P_{FDH1}
CustalO
LALIGN
P_{DAS1} vs. P_{DAK1}
CustalO
LALIGN: No similarities

D

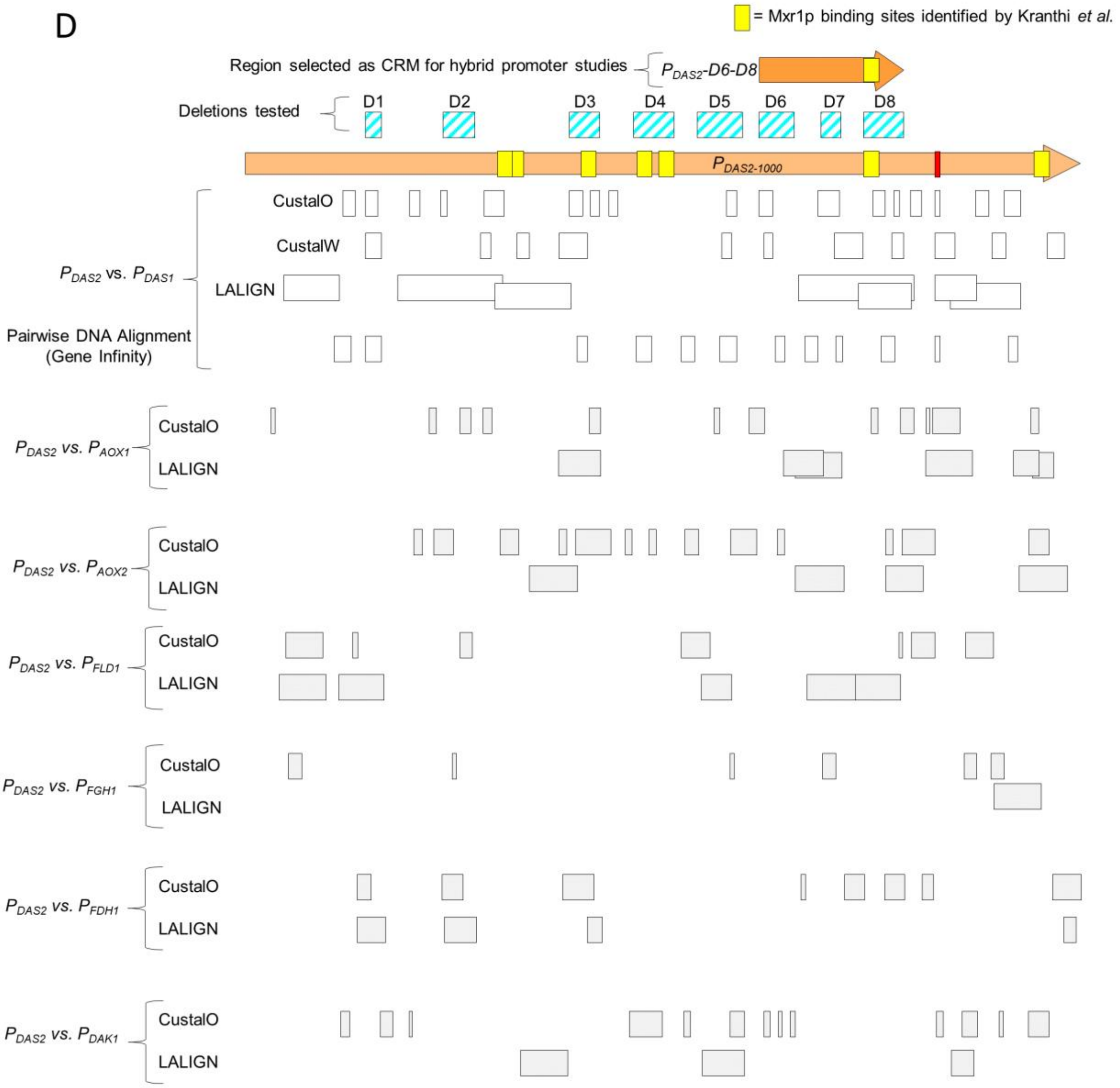
= Mxr1p binding sites identified by Kranthi et al.
Region selected as CRM for hybrid promoter studies
P_{DAS2}-D6-D8
Deletions tested
D1
D2
D3
D4
D5
D6
D7
D8
$P_{DAS2-1000}$
CustalO
CustalW
P_{DAS2} vs. P_{DAS1}
LALIGN
Pairwise DNA Alignment (Gene Infinity)
P_{DAS2} vs. P_{AOX1}
CustalO
LALIGN
P_{DAS2} vs. P_{AOX2}
CustalO
LALIGN
P_{DAS2} vs. P_{FLD1}
CustalO
LALIGN
P_{DAS2} vs. P_{FGH1}
CustalO
LALIGN
P_{DAS2} vs. P_{FDH1}
CustalO
LALIGN
P_{DAS2} vs. P_{DAK1}
CustalO
LALIGN

E

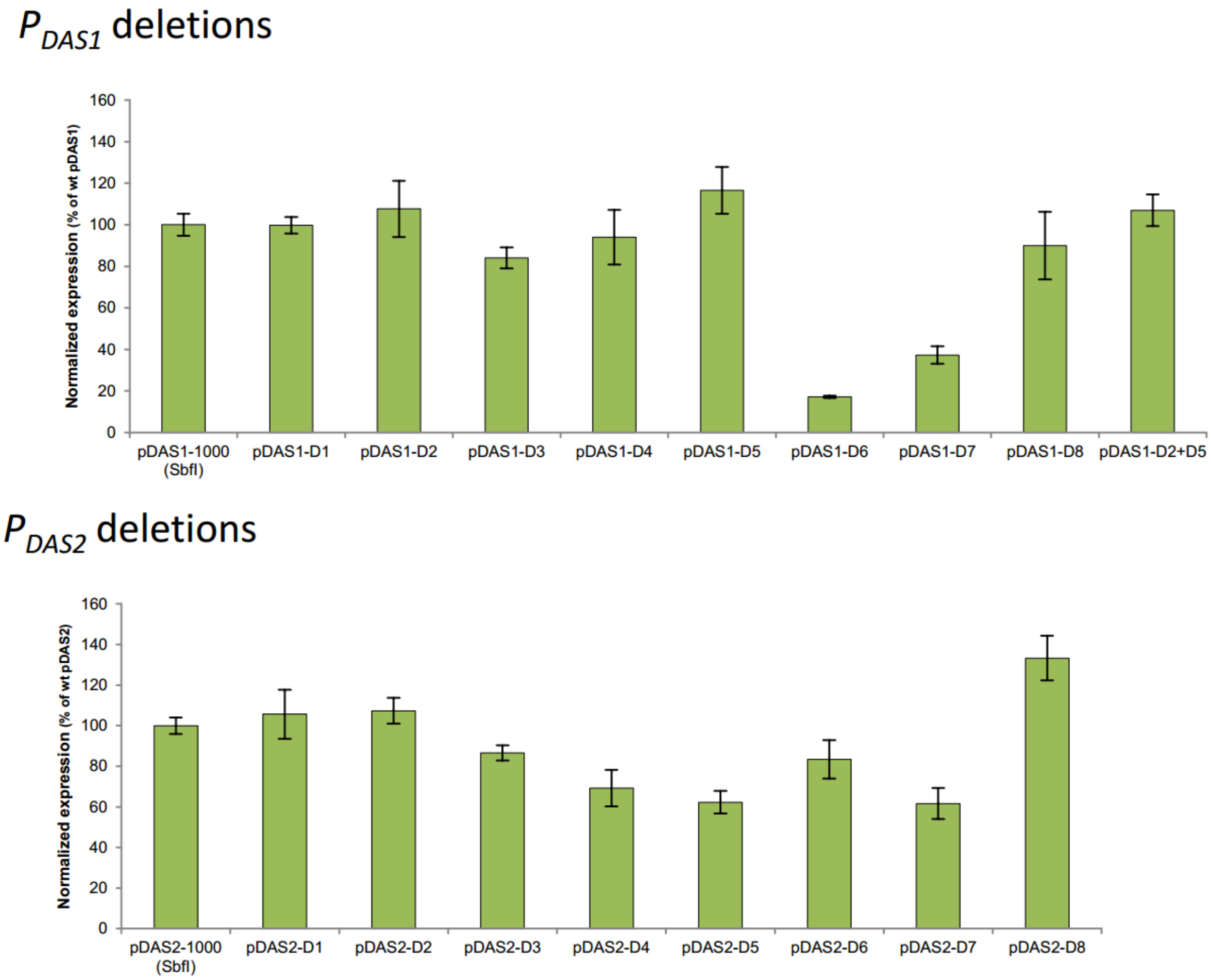

P DAS1 deletions
Normalized expression (% of wt pDAS1)
160
140
120
100
80
60
40
20
0
pDAS1-1000 (SbfI)
pDAS1-D1
pDAS1-D2
pDAS1-D3
pDAS1-D4
pDAS1-D5
pDAS1-D6
pDAS1-D7
pDAS1-D8
pDAS1-D2+D5
P DAS2 deletions
Normalized expression (% of wt pDAS2)
160
140
120
100
80
60
40
20
0
pDAS2-1000 (SbfI)
pDAS2-D1
pDAS2-D2
pDAS2-D3
pDAS2-D4
pDAS2-D5
pDAS2-D6
pDAS2-D7
pDAS2-D8

F

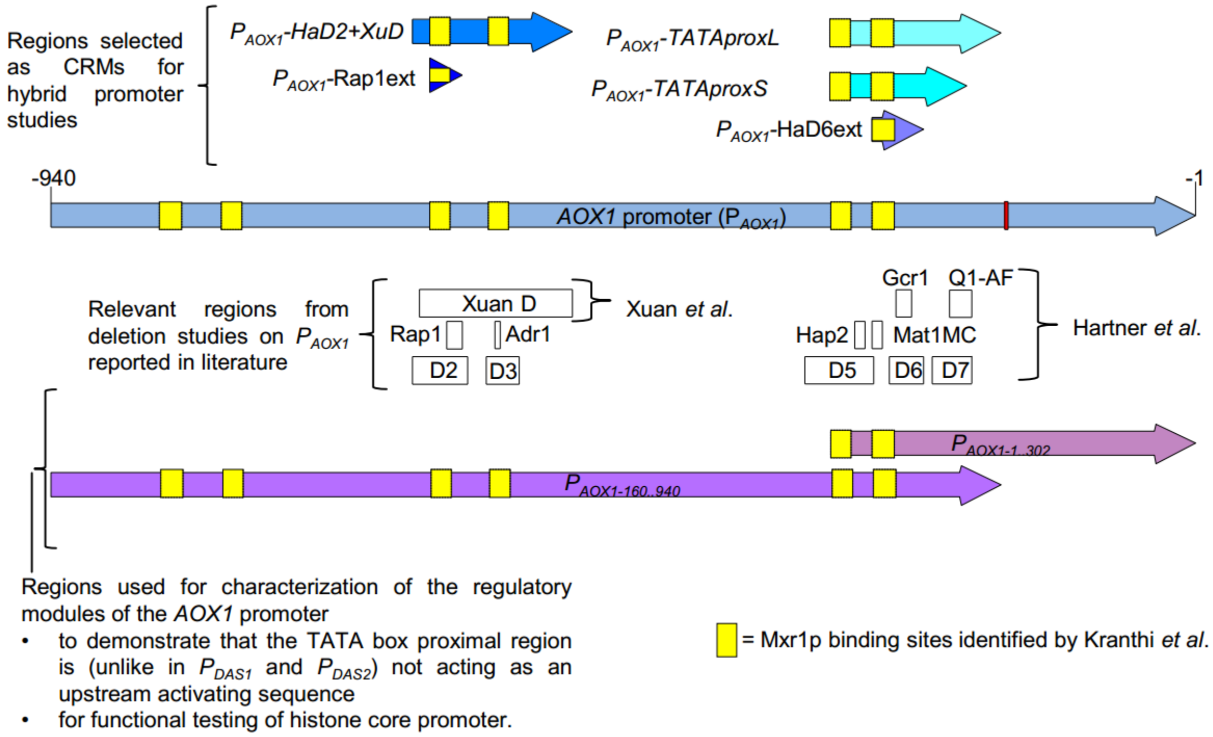

Regions selected as CRMs for hybrid promoter studies
P AOX1-HaD2+XuD
P AOX1-Rap1ext
P AOX1-TATAproxL
P AOX1-TATAproxS
P AOX1-HaD6ext
-940
-1
AOX1 promoter (P AOX1)
Relevant regions from deletion studies on P AOX1 reported in literature
Xuan D
Rap1
Adr1
D2
D3
Xuan et al.
Gcr1
Q1-AF
Hap2
Mat1MC
D5
D6
D7
Hartner et al.
P AOX1-1..302
P AOX1-160..940
Regions used for characterization of the regulatory modules of the AOX1 promoter
• to demonstrate that the TATA box proximal region is (unlike in P DAS1 and P DAS2) not acting as an upstream activating sequence
• for functional testing of histone core promoter.
= Mxr1p binding sites identified by Kranthi et al.

Supplementary Figure 2: Characterization of the natural bidirectional *P. pastoris DAS1/DAS2* promoter, deletion variants and regulatory elements selected form the *AOX1* promoter.

A) Genomic organization of the *P. pastoris DAS1/DAS2* locus (based on Figure 2 of Vogl and Glieder [2]) and the promoter lengths tested in this study. Most lengths were tested with a single fluorescent protein (eGfp, indicated by single arrows), a subset also with two fluorescent proteins (dTom and eGfp, double arrows). The *Sbf*I site in the 5' end of $P_{DAS1\text{-}1000}$ and $P_{DAS2\text{-}1000}$ was used for cloning the deletion variants outlined below and did not affect expression.

B) Reporter gene fluorescence measurements of the promoters shown in panel A (after 48 h of methanol induction).

C) Schematic overview on deletion studies on $P_{DAS1\text{-}1000}$ for the generation of variants with altered expression (panel E and Fig. 3c) and selected CRMs used for hybrid promoter design (Fig. 4 and Supplementary Figure 4). The deleted regions termed D1 to D8 were selected based on sequence similarities to the promoters of methanol regulated *P. pastoris* genes (*DAS1/DAS2*, *AOX1*, *AOX2*, *FLD1*, *FGH1*, *FDH1* and *DAK1* [3]). Similar stretches from pairwise alignments using ClustalO and LALIGN are shown. For the *DAS1/DAS2* comparison Clustal W and a pairwise alignment was performed in addition. Stretches appearing multiple times were selected for the deletions.

D) Same as C) for $P_{DAS2\text{-}1000}$. Binding sites of the methanol master regulator Mxr1 reported by Kranthi *et al.* [4] are depicted.

E) Effects of single deletions depicted in panels (c) and (d) (top panel P_{DAS1}, bottom panel P_{DAS2}). eGfp reporter fluorescence (rfu OD^{-1}) of the deletion variants was normalized as percent of the unmodified wildtype promoters (pDAS1-1000, pDAS2-1000).

F) *AOX1* CRMs used shown schematically, highlighting previously deleted regions by Xuan *et al.* and Hartner *et al.* [5,6].

In panels A and E of this figure mean values and standard deviations of normalized (using the normalization factor calculated in Supplementary Figure 1 reporter protein fluorescence measurements of biological quadruplicates grown on the respective carbon sources are shown).

See Supplementary Note 3 for the rationale of selecting the respective regions.

Supplementary Figure 3 (Synergistic and antagonistic effects observed for fusions of inducible and constitutive promoters)

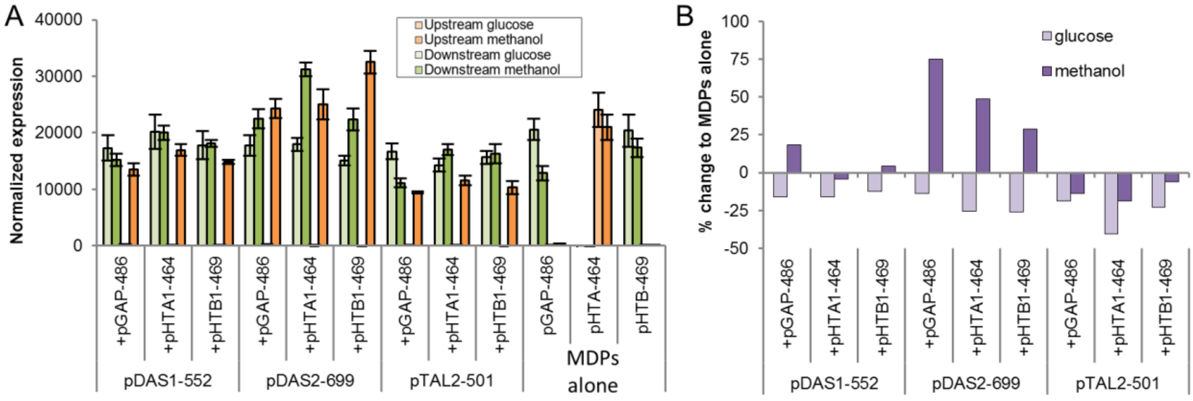

Supplementary Figure 3: Synergistic and antagonistic effects observed for fusions of inducible and constitutive promoters.

A) Comparison of bidirectional P_{GAP}, $P_{HTA1\text{-}464}$ and $P_{HTB1\text{-}469}$ fusions to methanol regulated promoters ($P_{DAS1\text{-}552}$, $P_{DAS2\text{-}699}$ and $P_{TAL2\text{-}501}$) with the MDPs alone. The same data shown in Fig. 3b and Supplementary Figure 4c was rearranged to facilitate comparisons. Hence, mean values and standard deviations of normalized (using the normalization factor calculated in Supplementary Figure 1) reporter protein fluorescence measurements of biological quadruplicates grown on the respective carbon sources are shown.

B) Changes in normalized reporter gene fluorescence of the fusions promoters compared to the MDPs alone from panel A are shown.

Fusions of growth-associated/constitutive *HTA1*, *HTB1* and *GAP* promoters to P_{DAS2} reached on methanol 1.3- to 1.8-fold increased expression compared to the single promoters. Notably, P_{GAP} is typically downregulated on methanol ([2], Supplementary Figure 1), whereas fusions to P_{DAS2} showed increased expression suggesting a transcriptional 'spillover' from the methanol inducible promoters. In consistency with these results, the $P_{DAS2\text{-}699}$ fragment had also given high expression when fused to a core promoter (Fig. 3a) underlining the strongly activating effect on upstream fusions. Fusions of the same growth-associated/constitutive promoters to $P_{DAS1\text{-}552}$ showed less pronounced effects.

However all promoters fused to $P_{TAL2\text{-}501}$ show decreased expression, on both carbon sources tested. Most strikingly the fusion of $P_{TAL2\text{-}501}$ to $P_{HTA1\text{-}464}$ shows on methanol a 41% decrease compared to the $P_{HTA1\text{-}464}$ promoter alone, suggesting a moderate repressing effect of the $P_{TAL2\text{-}501}$ sequence.

These results show that fusions of two differently regulated MDPs may interfere, affecting expression strength. Synergistic and antagonistic effects vary even between similarly regulated (*i.e.* methanol inducible) promoters. To this end, the properties of fusion promoters cannot entirely be foreseen and should be tested with reporter genes. However, the synergistic effects can be harnessed to design shorter, more efficient promoters and we expanded this principle for the design of hybrid promoters (Fig. 4).

Supplementary Figure 4 (Hybrid promoter design details)

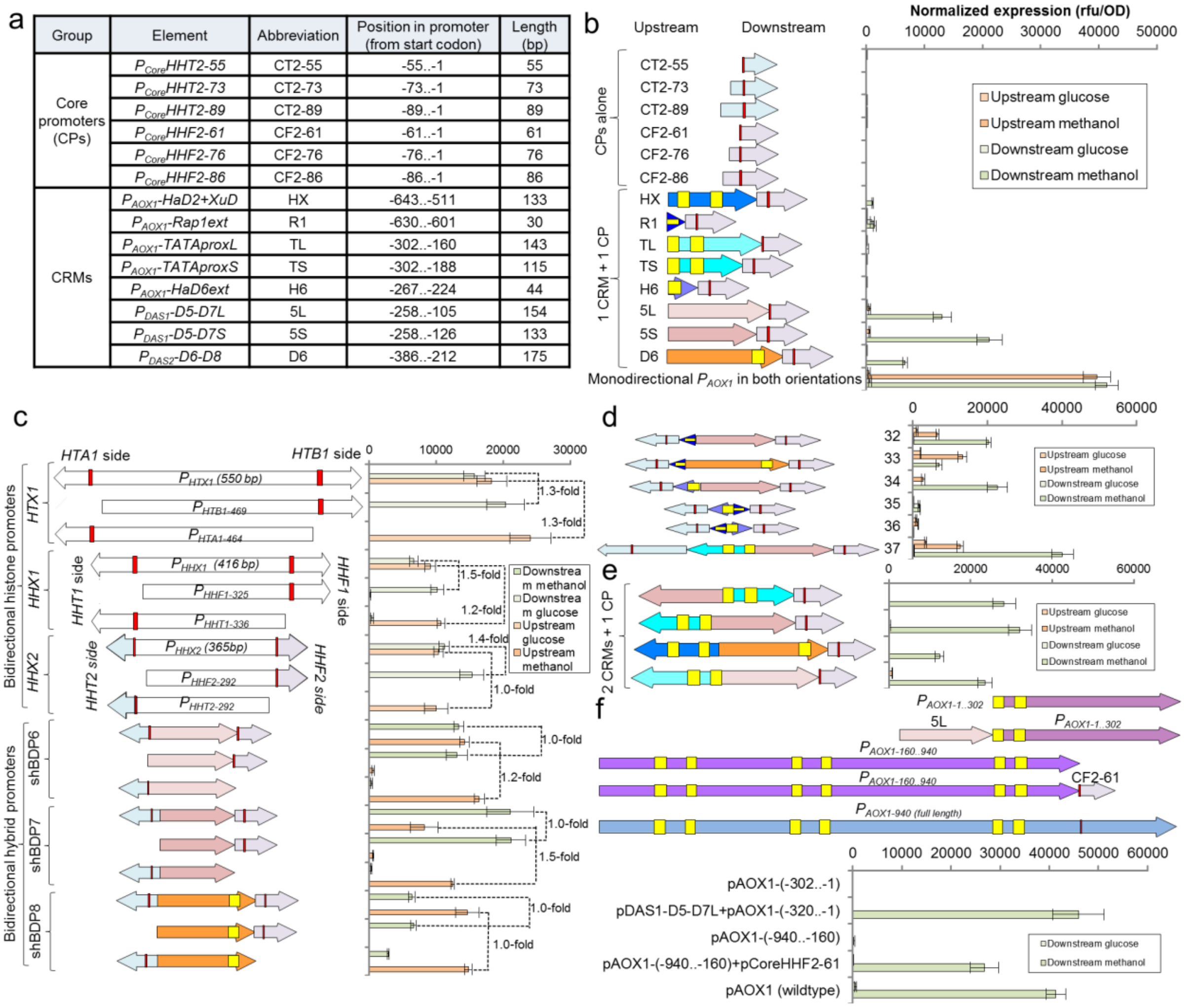

Supplementary Figure 4: Detailed design considerations and control constructs for bidirectional hybrid promoters in *P. pastoris*.

A) Regulatory elements used for the hybrid BDP design (see Fig. 2b for histone core promoters; Supplementary Figure 2 panel (c) for P_{DAS1}, (d) for P_{DAS2} and (f) for P_{AOX1} for illustrations of the elements in the natural promoter context).

B) Reporter protein fluorescence of histone CPs alone and combinations of the CRMs with a single CP. The *HHX2* CP lengths tested alone do not show any expression. Normalized fluorescence measurements after 60 h growth on glucose and 48 h of subsequent methanol induction are shown. The monodirectional P_{AOX1} is included as a control. The experimental cultivation conditions and the P_{AOX1} control apply to all panels.

C) Truncation of nBDPs (P_{HTX1}, P_{HHX1}, P_{HHX2}) and hybrid sBDPs (#6 to 8) on one side leads in 7/12 cases to increased expression on the other side. The nBDPs show the effect more pronounced (5/6) than the sBDPs tested (2/6). The data on the histone promoters is also shown in Fig. 2a in comparison to P_{GAP} and growth on glycerol. Values from growth on glucose are shown for the nBDPs, growth on methanol for the sBDPs. Fold changes of the truncated variant compared to the full length bidirectional promoter are shown.

D) Additional bidirectional hybrid promoter variants not included in Fig. 3d.

E) Additional monodirectional hybrid promoters (combinations of 2 CRMs with 1 core promoter).

F) Using the first 302 bp of P_{AOX1} as promoter element does not elicit any detectable reporter protein fluorescence despite containing two Mxr1p binding sites. Control constructs include

the fusion of a P_{DAS1}-*D5-D7L* activating sequence to the 302 core region, the *AOX1* promoter upstream sequence without a core promoter, a fusion of the upstream sequence to the *HHF2-61* core promoter and the full length wild type promoter.
In panels b-f of this figure mean values and standard deviations of normalized (using the normalization factor calculated in Supplementary Figure 1 reporter protein fluorescence measurements of biological quadruplicates grown on the respective carbon sources are shown.
See Supplementary Note 5 for interpretations of the results.

Supplementary Figure 5 (Molecular cloning of BDPs)

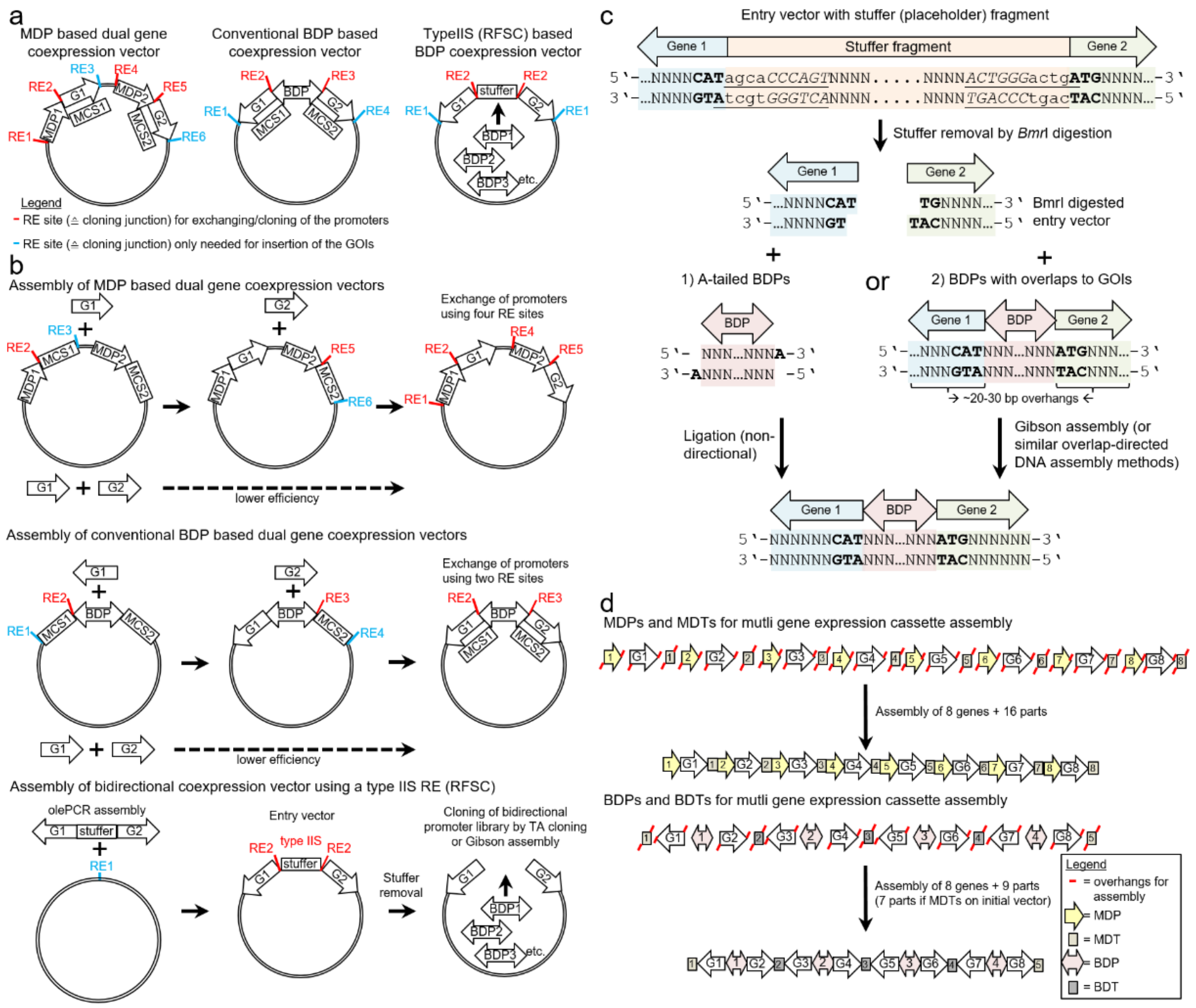

Supplementary Figure 5: Molecular cloning of BDPs via TA cloning or Gibson assembly facilitates optimization of dual and multi gene co-expression compared to MDPs.

A) Dual gene co-expression vectors based on BDPs and type IIS restriction endonucleases (REs) require less restriction sites/cloning junctions than MDP based vectors or conventional bidirectional vectors (*e.g.* [7,8]). We use the term ‘cloning junctions’ to refer to identical sequences required by overlap-directed DNA assembly methods such as Gibson assembly. ‘MCS’ elements depict multiple cloning sites required for cloning of the genes of interest (G1 and G2), ‘RSFC’: restriction site free cloning strategy [9]

B) Comparison of vector assemblies using MDP based vectors, conventional bidirectional vectors and the stuffer/typeIIS RE strategy reported here. Removal of a stuffer (placeholder) fragment from an entry vector using a single typeIIS RE enables the testing of a library of seamlessly linked BDPs.

C) Applying type IIS restriction endonucleases for seamless, sequence independent cloning (RSFC [9]) of BDPs by TA cloning [10] or providing junctions for Gibson assembly [11]. The start codons of the two genes are written bold, the entire *Bmr*I site is underlined and the recognition sequence is written in uppercase in italics.

D) Using BDPs and BDTs (bidirectional terminators) cuts the number of parts (promoters and terminators) approximately in half compared to MDPs and MDTs (monodirectional terminators) facilitating the assembly of multi gene expression cassettes. The assembly of eight genes is shown as an example. The bidirectional cloning (entry) vectors used in this study for inserting bidirectional multi gene expression cassettes provide already two MDTs, therefore the number of parts is reduced from nine to seven. See Supplementary Note 6 for additional details.

Supplementary Figure 6 (Reporter protein fluorescence dual gene co-expression)

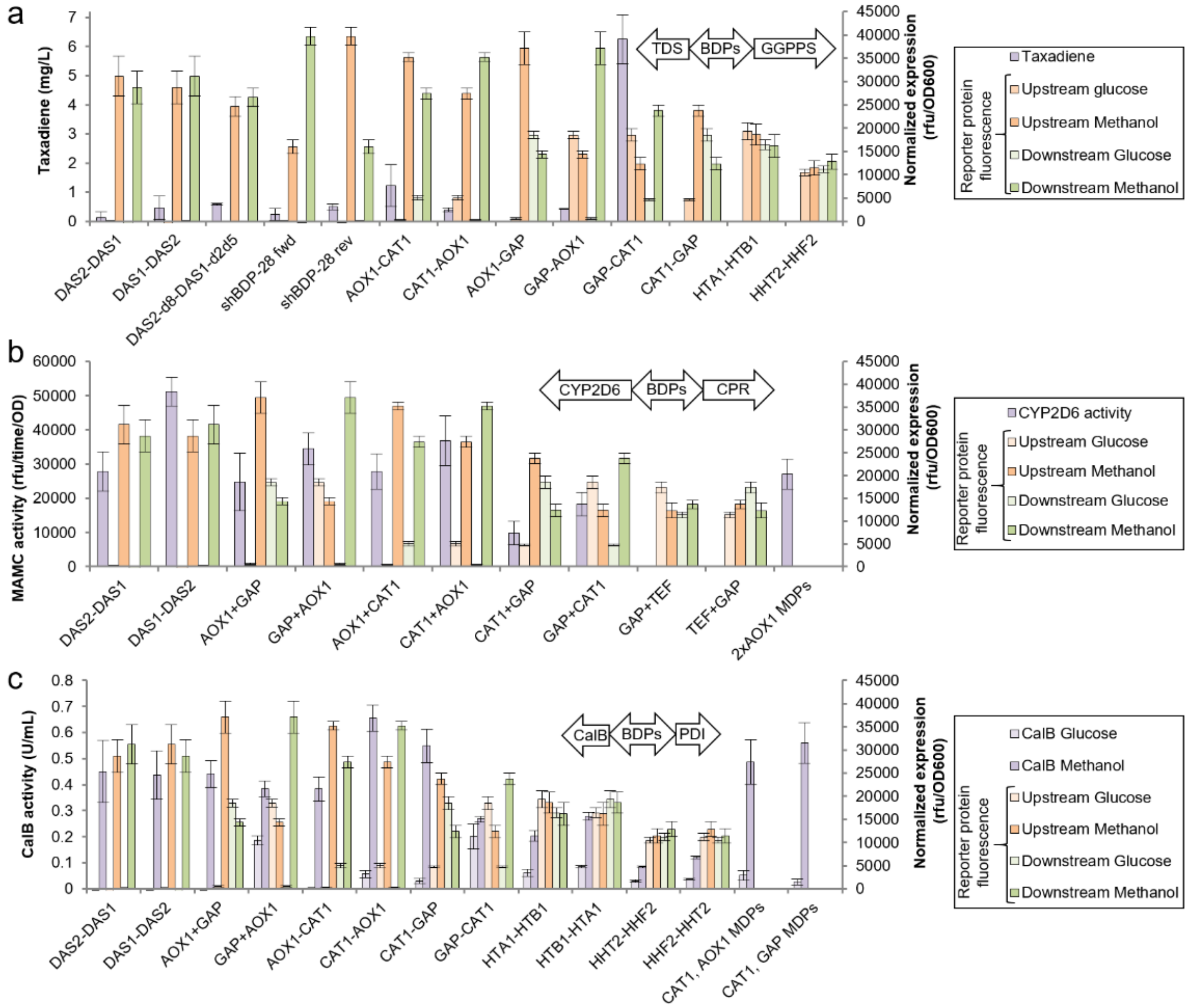

Supplementary Figure 6: Titers/activities of dual gene co-expression examples do not correlate with the respective reporter protein fluorescences of the BDPs used, highlighting the need for gene pair specific co-expression optimization. The same of data of the titers/activity measurements of taxadiene (a), CYP2D6 (b) and CalB (c) shown in Fig. 6a-c is combined with the reporter protein measurements of the respective promoters (shown in Fig. 1 to Fig. 4). Hence for all panels mean values and standard deviations from biological triplicates are shown for titers/activities, whereas fluorescence measurements were obtained from biological quadruplicates.

Supplementary Figure 7 (ARS function of bidirectional terminators, BDTs)

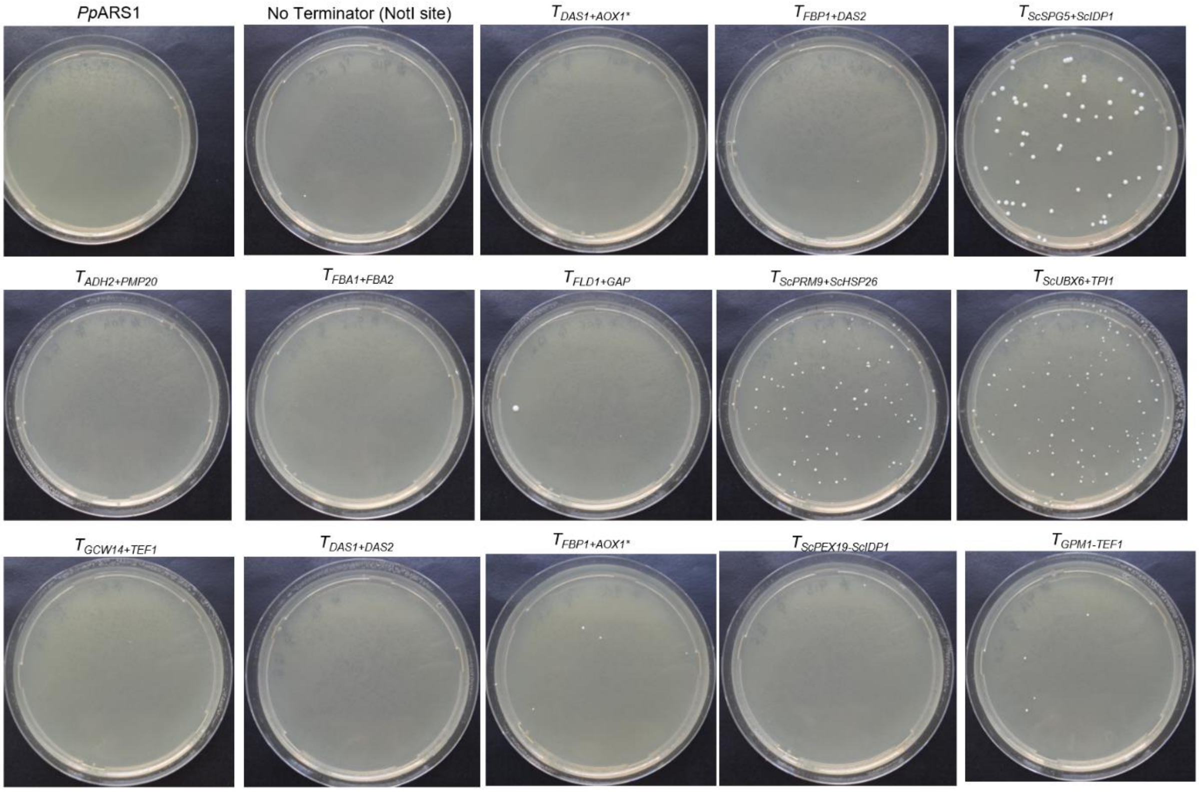

Supplementary Figure 7: Autonomously replicating sequence (ARS) function of the bidirectional terminators generated.

In *S. cerevisiae*, transcription termination and ARS function are associated [12]. In studies on *P. pastoris* MDTs [3] we noticed that some terminators showed ARS function, which may lead to increased background growth and strain instability of episomally replicating sequences. Therefore we tested all BDTs reported here for ARS function by transforming *P. pastoris* cells with 10 ng of the circular plasmids (see [3] for detailed information on the principle). We included a positive control of a *P. pastoris* ARS sequence [13] cloned into the vector backbone of the *P. pastoris* plasmid used (the same control was also included in previous work on MDTs [3]). All MDTs and BDTs were compared in a single experiment of which the MDT data was published [3], and its controls are also relevant, and therefore included here. The positive control showed pronounced growth of a few dozen colonies. The no terminator/empty vector/negative control (lacking a terminator and containing just a *Not*I site arising from self-ligating the vector) and most BDTs tested showed no or very few colonies. Few colonies are not a clear evidence for ARS function, as also circular plasmids can integrate into yeast genomes [14]. However circular plasmids show much lower efficiencies than linear DNA providing free ends [14] (which are typically generated for transformations of *P. pastoris* by linearizing the plasmids). Combinations of *S. cerevisiae* terminators showed the clearest ARS function (with $T_{ScSPG5+ScIDP1}$ being somewhat surpassing $T_{ScPRM9+ScHSP26}$ and $T_{ScUBX6+TPI1}$ judging from the colony sizes). These results match characterizations of the MDTs, where T_{ScSPG5} and T_{ScIDP1} had also shown clear ARS function [3]. The monodirectional T_{ScUBX6} had previously also shown termination function. Remarkably, monodirectional versions of T_{ScPRM9} had not shown colonies and $T_{ScHSP26}$ had shown only few colonies that we had previously not considered ARS function. Combination of these two sequences into $T_{ScPRM9+ScHSP26}$ did however show substantial ARS function, comparable to $T_{ScUBX6+TPI1}$. We recommend testing for ARS function of newly assembled BDTs to avoid issues with ARS background colonies and strain stability.

Supplementary tables

Supplementary Table 1: Detailed overview on bidirectional fusion promoters generated in Fig. 3b,c (see also the respective sheet in Supplementary Data 2).

Fusion BDP properties				Upstream promoter				Downstream promoter			
#	Functional group	Label of the fusion BDP in Fig. 3c	Length (bp)	Name	Regulation	Length (bp)	Ref.	Name	Regulation	Length (bp)	Ref.
1	Differently regulated promoters	AOX1+GAP	1426	AOX1	tightly repressed, methanol inducible	940	3	GAP	constitutive (naturally involved in glycolysis)	486	2
2		CAT1+GAP	1178	CAT1	derepressed, methanol inducible	692	3	GAP	constitutive (naturally involved in glycolysis)	486	2
3		DAS1+GAP	1038	DAS1	tightly repressed, methanol inducible	552	3	GAP	constitutive (naturally involved in glycolysis)	486	2
4		DAS1+HTA1	1016	DAS1	tightly repressed, methanol inducible	552	3	HTA1	'constitutive' (cell cycle regulated)	464	this work
5		DAS1+HTB1	1021	DAS1	tightly repressed, methanol inducible	552	3	HTB1	'constitutive' (cell cycle regulated)	469	this work
6		DAS2+GAP	1185	DAS2	tightly repressed, methanol inducible	699	3	GAP	constitutive (naturally involved in glycolysis)	486	2
7		DAS2+HTA1	1163	DAS2	tightly repressed, methanol inducible	699	3	HTA1	'constitutive' (cell cycle regulated)	464	this work
8		DAS2+HTB1	1168	DAS2	tightly repressed, methanol inducible	699	3	HTB1	'constitutive' (cell cycle regulated)	469	this work
9		TAL2+GAP	987	TAL2	tightly repressed, methanol inducible	501	3	GAP	constitutive (naturally involved in glycolysis)	486	2
10		TAL2+HTA1	965	TAL2	tightly repressed, methanol inducible	501	3	HTA1	'constitutive' (cell cycle regulated)	464	this work
11		TAL2+HTB1	970	TAL2	tightly repressed, methanol inducible	501	3	HTB1	'constitutive' (cell cycle regulated)	469	this work
12		TEF1+GAP	1486	TEF1	constitutive (naturally involved in translation)	1000	2	GAP	constitutive (naturally involved in glycolysis)	486	2
13	Inducible promoters	FDH1+PMP20	956	FDH1	derepressed, methanol inducible	564	3	PMP20	tightly repressed, methanol inducible	392	3
14		FDH1+DAS1	1116	FDH1	derepressed, methanol inducible	564	3	DAS1	tightly repressed, methanol inducible	552	3
15		FDH1+CAT1	1064	FDH1	derepressed, methanol inducible	564	3	CAT1	derepressed, methanol inducible	500	3
16		FLD1+AOX1	1009	FLD1	derepressed, methanol inducible	366	3	AOX1	tightly repressed, methanol inducible	643	3
17		FLD1+PMP20	758	FLD1	derepressed, methanol inducible	366	3	PMP20	tightly repressed, methanol inducible	392	3
18		CAT1+AOX2	1000	CAT1	derepressed, methanol inducible	500	3	AOX2	tightly repressed, methanol inducible	500	3
19		FBA2+TAL2	1001	FBA2	tightly repressed, methanol inducible	500	3	TAL2	tightly repressed, methanol inducible	501	3
20		DAS2+DAS1*	1251	DAS2	tightly repressed, methanol inducible	699	3	DAS1	tightly repressed, methanol inducible	552	3
21		AOX1+CAT1	1632	AOX1	tightly repressed, methanol inducible	940	3	CAT1	derepressed, methanol inducible	692	3

Supplementary notes

Supplementary Note 1 (*P. pastoris* gene organization analysis [Supplementary Data 1])

Analysis of genome organization
The genome sequence of the *P. pastoris* CBS7435 strain [15] was analyzed chromosome by chromosome (GenBank IDs: FR839628.1, FR839629.1, FR839630.1 and FR839631.1) for genes in head to head, tail to head (head to tail) and tail to tail orientation similar to the analysis of Trinklein *et al.* [16] of the human genome. Genes in head to head, head to tail and tail to tail orientation are provided in the separate sheets of the Excel file. In rare cases genetic elements such as tRNAs, rRNAs, mobile elements or sequencing gaps were annotated between two genes transcribed by RNA polymerase II. The presence of genetic elements is denoted in the Excel file, gene pairs separated by gaps were omitted from the analysis.

This search was limited to directly adjacent genes. Miss-annotations (or hypothetical genes present) may bias the results. For example the natural bidirectional *DAS1*/*DAS2* promoter is missing from the list of putative nBDPs, as a gene termed "Probable guanine nucleotide exchange factor FLJ41603 homolog " is between the *DAS1* and *DAS2* genes (Supplementary Figure 2a). To rule out bias of the annotation, the genes of the MUT pathway were manually curated for putative nBDPs.

Selection and testing of putative nBDPs
The list of head to head genes was searched for putative nBDPs of typical housekeeping genes (Fig. 1c) to obtain constitutive promoters. Gene pairs containing annotations with the terms "putative", "hypothetical", "uncharacterized" or "probable" were omitted from the analysis. We focused on genes of the central carbon metabolism, general transcription machinery and ribosomal proteins.

Genome wide absolute quantification of transcription by RNA sequencing (RNAseq) may facilitate nBDP characterization, since promising nBDP targets can be directly selected from their expression strength. However, at the time we started this study, no RNAseq data for *P. pastoris* was available and RNAseq studies in *P. pastoris* remain scarce (e.g. [17,18]). Yet, for widely studied model organisms such as *S. cerevisiae* with an abundance of RNAseq data studies at hand, pre-selection of putative nBDPs may considerably reduce screening efforts. Aside studies on cryptic/pervasive bidirectional transcription [19,20], so far only a DNA sequence based study on BDPs has been performed in *S. cerevisiae* comparing sequence features such as the presence of TATA boxes and transcription factor binding sites [21].

However even with RNAseq studies it may be impossible to find nBDPs with specific regulatory profiles, since they may not exist. For a library of BDPs to optimize gene co-expression, inducible nBDPs and combinations of inducible and constitutive promoter sides are desirable to fine tune expression in a time dependent manner. In *P. pastoris* only a limited set of methanol regulated promoters is known or anticipated [3] and we have tested all putative nBDPs with MUT promoters on one side (Fig. 1c).

Supplementary Note 2 (Details on reporter proteins normalization [Supplementary Figure 1])

Experimental outline and discussion

Due to different maturation times, quantum yields, stabilities and signal amplification by the fluorescence spectrometer used, the relative fluorescence measurements obtained from eGfp and dTom are not directly comparable. We designed a set of controls and determined a normalization factor between the two FPs. Therefore a set of promoters was cloned in forward and reverse orientation between the two FPs. Subsequently the eGfp and the dTom signals of the same side were compared. We included the monodirectional state of the art *AOX1* and *GAP* promoters and three bidirectional histone promoters. We also included control vectors with only a single FP present and cloned the *HHX2* promoter in both orientations into these vectors (the gene coding for the second FP was omitted and the promoter directly adjacent to the transcriptional terminator). These controls were performed to check for effects of coproduction of two FPs vs. production of a single FP.
Since we characterized constitutive and methanol inducible promoters, we compared the reporter fluorescence obtained from growth on glucose and different time points of growth on methanol (glycerol was also tested, but yielded similar results to glucose).

The normalization factors calculated from the different promoters (panel e) were in good agreement for each single time point measured. However, the mean value of the ratio/normalization factor for growth on glucose (a) was lower than for growth on methanol for 24 hours (b). When the cells were grown for a 48 h (c) and 72 h (d) on methanol, the normalization factors leveled off at similar values as on glucose. We assume that these effects are evoked by different maturation times of eGfp and dTom; as the eGfp variant was selected for improved folding: After 60 h growth on glucose both proteins have folded and accumulated, but after 24 h on methanol eGfp may be folding faster than dTom resulting in a higher eGfp/dTom fluorescence ratio. It appears that after 48 h and 72 h enough time has passed to allow dTom folding, resulting in a similar ratio as on glucose. This finding implies that for every measured time point the respective normalization factor has to be used. For the normalizations shown in the main manuscript and the supplementary materials, the values of 60 h growth on glucose and 48 h methanol induction were used. We had initially also tested alternative combinations of FPs and variants of Tomato but found the eGfp and dTom combination most suitable.

Flow cytometry measurements (*e.g.* FACS) provide more detailed information on the cell population measured, whereas the fluorescence plate reader measurements performed here give only a cumulative signal of the entire population. However, the FACS devices available to us did not provide the correct filter to clearly discriminate the signals of eGfp and dTom. The high throughput characterization of the 168 *P. pastoris* BDPs was rendered possible by the availability of a monochromator based 96 well fluorescence microtiter plate reader. Notably also alternative filter based fluorescence plate readers considered did not provide by default the suitable filter sets to unambiguously discriminate eGfp and dTom fluorescence. We performed these extensive controls to ensure reliability of our plate reader measurements and we have previously shown that FACS and plate reader measurements of promoter variants are in excellent agreement in *P. pastoris* [22].

Possible interference of dTomato and OD600 measurements

In *E. coli,* it has been reported by Hecht et al. [23], that red fluorescent protein expression can bias OD600 measurements and thereby estimates for biomass. Such effects of high dTom fluorescence on OD600 measurements may theoretically also occur in *P. pastoris*. However, if there had been a major bias, our normalization experiments, where eGfp and dTom fluorescence were extensively compared, would have revealed it. We had also specifically looked at cell growth of dTom expressing cells in comparison

with the wildtype strain in previous work [24] [supporting figure S6 in that paper] and had not noticed an impact of dTom expression. It is worth noting that after correction factor application, the histone promoters give nearly identical expression on both sides. Additionally, the *AOX1* promoter tested in both directions (for example shown in Fig. 4, control at the very bottom bottom) gives nearly identical values if tested with eGFP or dTOM. Thus, if there were an interference with OD600 measurements as noted by Hecht et al., it would be evident for these controls.
However why did we not experience the issues reported by Hecht et al.?

We assume that there are two main reasons:

1.) We performed the experiments in the yeast *P. pastoris* and not in the bacterium *E. coli*. It appears plausible that the ratios between fluorescent proteins inside of the cells and the biomass is different between bacteria and yeasts. *P. pastori*s is known for growth to exceptionally high cell densities (up to 500 g L^{-1} cell wet weight in bioreactors and even in DWPs ODs of ~20 to 30 can be reached) whereas the fluorescent protein production does not necessarily increase the same way. Hence a 10x denser *P. pastoris* culture might actually contain relatively less protein than *E. coli* cells. Hence there could be simply relatively less fluorescent protein interfering with the biomass measurement.

2) We have used a different red fluorescent protein variant than Hecht et al. with notable differences in excitation/emission wavelengths: We have used dTomato (excitation/emission wavelength: 554/581 nm [1]) whereas Hecht et al. have used mRFP1 (excitation/emission wavelength: 584/607 nm; original mRFP1 reference: [25]). So the em/ex peaks are shifted by 30/26 nm respectively, placing mRFP1 used by Hecht et al. notably closer to 600 nm used for OD600 measurements. Hence it appears plausible that the issue noted by Hecht et al. does not occur for all RFP variants.

Note that while such interference effects appear to have no effect on the use of dTom in *P. pastoris*, they might be as relevant as in *E. coli* [23]in other organisms or with other fluorescent proteins and should be tested for.

Supplementary Note 3 (Rationale behind $P_{DAS1/DAS2}$ deletion variants and regulatory elements shown in Supplementary Figure 2)

Selection of deleted regions

Deletion studies of promoters have been used in various organisms to identify regulatory regions and to generate variants with altered expression levels applicable as promoter library for fine tuning gene expression [5,26]. Either systematic deletions were performed (*i.e.* adjacent fragments [6,27]) or semi-rational considerations (such as the prediction of transcription factor binding sites [5]) were applied, as exemplified by studies on the *P. pastoris AOX1* promoter [5,6,27].

Here we used a different approach to select relevant regions for deletion in *DAS1* and *DAS2* promoters (although systematic deletion studies or TFBS predictions would likely yield similar results). Based on the recent finding that several promoters of the *P. pastoris* methanol utilization pathway are similarly regulated [3], we reasoned that this co-regulation must be conferred by conserved sequence DNA stretches. Therefore we selected a set of eight methanol inducible and glucose repressed promoters including the *DAS* promoters to search for shared elements (*DAS1*, *DAS2*, *AOX1, AOX2, FLD1, FGH1, FDH1* and *DAK1*). However, TFBSs may be placed at different positions between promoters. Studies on the *P. pastoris* methanol master regulator Mxr1p [4,28] showed that its binding sites are arranged pairwise over the whole *AOX1* promoter [28], whereas they are generally closer together in the *DAS2* promoter [4] (reviewed in [2] and compare the Mxr1 binding sites in Supplementary Figure 2d and f). In addition, yeast TFBS are often short and degenerate as exemplified by the Mxr1 consensus sequence CYCCNY (N = any base, Y = C or T) [4].

Performing a multiple sequence alignment of the eight MUT promoter sequences mentioned above using Clustal Omega [29] in standard settings did not show clearly conserved regions. Therefore we performed pairwise comparisons of the *DAS1* and *DAS2* promoters with the other promoters, including LALIGN analysis in the standard settings (LALIGN is suitable to identify local sequence similarities between two sequences [30]). Sequences appearing multiple times were selected for deletion.

Effects of the single deletions

Several deletion variants showed up to 1.33-fold increased expression compared to the full length wildtype promoters (1000 bp length) suggesting either removal of repressor binding sites or beneficial effects from rearranging the spacing. Most strikingly, deletions D6 and D7 in the *DAS1* promoter led to a strong decrease in expression (17 and 37% of unmodified control), suggesting loss of a major activating region. Deletion of several regions in P_{DAS2} also had a negative impact on reporter fluorescence, however not as drastically as in P_{DAS1} (62% of 1000 bp unmodified control).

Supplementary Note 4 (Bidirectionalization and fusion design considerations)

Design consideration for bidirectionalizations

We selected differently regulated monodirectional *P. pastoris* promoters for bidirectionalization, including methanol inducible, tightly repressed promoters of the *AOX1, PMP20, DAS1, DAS2, FBA2* and *TAL2* genes. Also methanol inducible but derepressed promoters of *CAT1, FLD1* and *FDH1* genes were tested [3]. Furthermore constitutive promoters of the *GAP, TEF* and *ADH2* genes were bidirectionalized. The short histone core promoters outlined in Fig. 2b were in reverse orientation fused to different lengths of the MDPs. Results on the bidirectionalizations are shown in Fig. 3a of the main manuscript.

Design consideration for fusions promoters

To achieve combinations of regulatory profiles, we fused differently regulated MDPs to each other in reverse orientation (see also the respective sheet in Supplementary Data 2). In addition, we included specifically combinations of two inducible promoters, to obtain suitable tools for regulated pathway overexpression. The properties of the combinations tested are summarized in in Supplementary Table 1 (results are shown Fig. 3b of the main manuscript).

Combinations of PDAS1 and PDAS2 deletions

We generated additional methanol inducible fusion BDPs with varying expression ratios by combing different monodirectional deletion variants (Fig. 3c). We combined deletions showing increased reporter gene fluorescence in the monodirectional context (Supplementary Figure 2e) into improved BDPs (*e.g*. *DAS2-D8+DAS1-D2*, *DAS2-D6+DAS1-D2D5*). Also BDPs with decreased expression were generated (*DAS2-D5+DAS1-D6*, *DAS2-386+Das1-D6*). Altered ratios between both sides (*DAS2-D8+DAS1-D6*) were generated by fusing weaker monodirectional variants. $P_{DAS2\text{-}386}$ and $P_{DAS\text{-}261}$ are additional truncated variants to reduce expression from the *DAS2* side (since monodirectional P_{DAS2} deletions had only shown a decrease to 62% of the unmodified control).

Supplementary Note 5 (Hybrid sBDPs results interpretation)

Selection of CRMs for the generating the hybrid BDPs shown in Fig. 4 and Supplementary Figure 4

Various synthetic monodirectional hybrid promoter have been engineered by fusing CRMs to core promoters [26,31]. We extended this strategy to BDPs, by flanking CRMs with two core promoters in opposite orientation. We used the short *HHX2* histone core promoters (Fig. 2b) successfully applied for bidirectionalization of MDPs (Fig. 3a). Six short CRMs (30 to 175 bp) from methanol regulated promoters (P_{AOX1}, P_{DAS1}, P_{DAS2}) were used. Namely, four elements from P_{AOX1} (P_{AOX1}*-HaD2+XuD*, P_{AOX1}*-Rap1ext*, P_{AOX1}*-TATAproxL/S,* P_{AOX1}*-HaD6ext*) and a single element from each P_{DAS1} (P_{DAS1}*-D5-D7L/S*) and P_{DAS2} (P_{DAS2}*-D6-D8*) were used.

CRMs from the *AOX1* promoter were selected based on deletion studies reported in the literature ([5,6,27,32] reviewed in [2]) and binding sites reported for Mxr1p (zinc finger transcription factor and master regulator of MUT genes in *P. pastoris*) [28]. The CRMs of the *AOX1* promoter contain Mxr1p binding sites and deletions within these regions strongly affected expression [5,6,28]. P_{AOX1}*-HaD2+XuD* is a fusion of the D2 region of Hartner *et al.* [5] and region D of Xuan *et al.* [6] containing two Mxr1p binding sites [28]. P_{AOX1}*-Rap1ext* is a putative TFBS reported by Hartner *et al.* extended to contain an Mxr1p binding site. P_{AOX1}*-TATAproxL/S* contains two Mxr1p binding sites and several deletions in this region drastically affected expression. Due to its proximity to the TATA box we refer to this CRM as 'TATAprox'. P_{AOX1}*-HaD6ext* is the region D6 characterized by Hartner *et al.* extended to comprise the adjacent Mxr1p binding site.

CRMs from P_{DAS1} and P_{DAS2} were selected based on deletion studies performed in frame of this work (Supplementary Figure 2, Supplementary Note 3) and Mxr1p binding sites reported for P_{DAS2} [2,4]. Variants with deletions of the regions D6 to D7 in the *DAS1* promoter showed strongly decreased expression, suggesting the presence of a major activating region. We extended this region to include the D5 region and tested it due to its close proximity to the core promoter/TATA box in two lengths (termed P_{DAS1}*-D5-D7L* and P_{DAS1}*-D5-D7S*). Deletions in the *DAS2* promoter had not shown as drastic effects as in the case of P_{DAS1}, however deletion of region D7 had notably reduced expression. We extended this sequence stretch to the adjacent elements resulting in P_{DAS2}*-D6-D8.*

CRMs adjacent to the core promoter/TATA box were in part tested in different lengths (P_{AOX1}*-TATAproxL/S* and P_{DAS1}*-D5-D7L/S;* 'L' for long, 'S' for short) to probe for carryover effects of the core promoter. The long variants of these CRMs were extended up to the TATA box (fusions of these CRMs with core promoters reconstitute the natural position of the TATA box in both core promoter and CRM).

Truncation of BDPs on one side leads in 7/12 cases to increased expression on the other side.

We had noticed in the deletion and truncations studies of P_{HHX2} (Fig. 2c,d) that removal of the core promoter from one sides increases expression from the opposite side. To confirm this effect we also truncated the core promoters from the histone promoters P_{HTX1} and P_{HHX1} and synthetic bidirectional constructs shBDP6 to shBDP8 (panel C). For P_{HTX1} we removed the 86 bp long *HTB1*-core promoter ($Pcore_{HTB1-86}$, Fig. 2b) resulting in a truncated P_{HTA1} promoter of 464 bp ($P_{HTA1-464}$). *Vice versa* the core promoter removal/truncated promoter pair on the other side of P_{HTX1} is $Pcore_{HTA1-81}$/$P_{HTB1-469}$. The pairs for P_{HHX1} are $Pcore_{HHT1-91}$/$P_{HHF1-325}$ and $Pcore_{HHF1-80}$/$P_{HHT1-336}$. For P_{HHX2} the truncations F1 and T1 already shown in Fig. 1g were used. For shBDP6 to shBDP8 we tested the CRMs flanked by two core promoters simultaneously and also a single core promoter on each side (panel C).

In 7 of 12 cases removal of a core promoter increased expression from the other side (up to 1.5-fold). This may be caused by transcriptional or translational effects: The two core promoters could be competing for RNA polymerase II (RNAPII) and general transcription factors. Alternatively transcription could be unaffected and solely the protein level affected. Producing two FPs at the same time may require more resources in the form of amino acids and capacities for protein synthesis/folding from the cell and represent a greater metabolic burden [33] than expressing a single FP. If the burden of a second protein is removed, translation of the single one may be stronger.

For P_{HHX2} we assume that the effect is transcriptional and not translational: In frame of the normalization work to compare the two FPs used (Supplementary Figure 1, Supplementary Note 2), we created constructs of the full length *HHX2* promoter flanked by 1.) two FPs and 2.) one FP and one transcriptional terminator (in both orientations: transcription terminator on the 5' end [directly next to the promoter] and a FP gene on the 3' end or *vice versa* [terminator on the 3' end and FP gene on the 5' end]). Thereby the bidirectional promoter is on one side expressing the FP, but on the other side transcription is immediately stopped after the 5'UTR by the terminator. Expression of these terminator constructs was not increased compared to deletion of the core promoters (Fig. 2d). Therefore we conclude that removal of the core promoters and not just the reporter gene is required to increase expression. Hence it appears that *P. pastoris* cells have sufficient resources to produce two FPs at high levels and translation is not the limiting factor, hinting a regulatory model in which two core promoters are competing for transcription initiation by general TFs or RNAPII in a bidirectional context (possibly in line with studies in higher eukaryotes showing that antisense promoter transcription commonly relies on the sense core promoter sequence [34]).

Transcriptional 'spillover' in hybrid promoters

In hybrid BDPs created by fusions of the growth-associated $P_{HHT2\text{-}T146}$ to glucose repressed/methanol regulated CRMs, similar antagonistic/synergistic 'spillover' effects as seen with some fusion promoters (Supplementary Figure 3) were observed (hybrid promoters #9-14 in Fig. 3d). If the methanol regulated CRMs were not fused to any additional sequences (Supplementary Figure 4b) or other methanol regulated CRMs (Fig. 3d), they were tightly repressed on glucose. However, if they were fused to the growth associated active $P_{HHT2\text{-}T146}$, they showed already on glucose clear reporter protein fluorescence. This effect depended on the CRM, but suggests that the growth associated expression of the $P_{HHT2\text{-}T146}$ to glucose repressed/methanol regulated CRMs partially alleviates the repression.
We did not observe so strong effects with longer fusion promoters (Fig. 3b), presumably as these promoters were considerably longer and regulatory regions not directly adjacent. The use of insulator sequences may also abolish the spill-over in hybrid promoters consisting of $P_{HHT2\text{-}T146}$ fusions to glucose repressed/methanol regulated CRMs. In *S. cerevisiae* DNA binding factors have been reported (Tbf1 and Mcm1), that could be used for this purpose [35].

In some respect, certain aspects of our work would rather support models from openly debated deep sequencing studies (e.g. [36–38]), in which divergent/bidirectional transcription is arising from second (cryptic) upstream core promoters (in yeast pre-initiation complexes form around both sides of nucleosome free regions associated with transcribed chromatin [39]). We have here shown that most CRMs tested were suitable to trigger upstream expression if a second core promoter was added. Hence, cryptic core promoter sequences naturally occurring upstream of CRMs/enhancers may fulfill a similar role as rationally added core promoters in our experiment (also given the fact that even random sequences can show substantial functionality as core promoters [22]). Hence we rather tend to lend credence to the notion, that widespread bidirectionality observed in deep sequencing studies is presumably caused by the presence of cryptic upstream core promoters alongside an orientation independent recruitment of RNAPII by activators binding CRMs. Whether these cryptic/weak core promoters are evolutionary shaped and their expression serves a purpose (or is a merely an

unavoidable side effect of the orientation insensitivity of the recruitment mechanisms of transcription factors) has recently been comprehensively studied [40].

The hybrid promoter assemblies and additional controls suggest different promoter architectures for PAOX1 and PDAS1

In all our studies of the hybrid promoters, the CRMs close to the TATA box and the 3' end of the *AOX1* promoter did not show any activity (P_{AOX1}-*TATAprox*). This finding is surprising as a CRM from the *DAS1* promoter (P_{DAS1}-*D5-D7*) stemming from a similar 3' region close to the TATA box, did show strong activation in all contexts tested (Fig. 4 and Supplementary Figure 4).

These effects could be caused by an incompatibility of the *AOX1* CRM with the histone core promoters or indeed a lack of activating sequences.

We performed additional controls in Supplementary Figure 4f, by testing the natural context of the *AOX1* CRM fused to the *AOX1* core promoter ($P_{AOX1\text{-}1..302}$). This part alone did not show any detectable reporter fluorescence. Fusion of the entire sequence upstream of the TATA box of the *AOX1* promoter ($P_{AOX1\text{-}160..940}$) to the histone core promoter of the *HHF2* gene, showed expression matching the wild type *AOX1* promoter. The negative control of the $P_{AOX1\text{-}160..940}$ sequence alone does not show any expression. These experiments rule out that the problem is arising from the fusion of the CRM to the histone core promoter. Fusion of the P_{DAS1}-*D5-D7* CRM to the $P_{AOX1\text{-}1..302}$ sequence restores P_{AOX1} wild type like expression levels.

It indeed seems that the TATA proximal region of P_{AOX1} does not have any activating function whereas the similar region in P_{DAS1}-*D5-D7* leads to strong activation. It is also puzzling, that the TATA proximal region of P_{AOX1} contains two experimentally confirmed binding sites for Mxr1p, a master activator for methanol inducible genes in *P. pastoris* [28]. The full length *AOX1* and *DAS1* promoters are however similarly regulated (tight glucose repression, strong methanol induction Fig. 1c). These results highlight the variability and flexibility of yeast promoters, achieving similar regulation by vastly different promoter architecture.

Supplementary Note 6 (Molecular cloning & overlap directed cloning methods for BDPs)

BDPs facilitate molecular cloning for dual gene and multi gene co-expression

With a suitable library of 168 BDPs at hand, we devised a cloning strategy and optimized vectors for applying the BDPs for dual gene and multi gene co-expression (Supplementary Figure 5). Using conventional MDPs for optimization of dual gene co-expression requires in total six unique RE sites to insert genes of interest (GOIs) and testing different promoters (Supplementary Figure 5a,b). Currently used bidirectional vectors (*e.g.* [7,8]) rely on a fixed bidirectional promoter and sequential cloning steps using multiple cloning sites (MCSs [requiring at least four RE sites]) (Supplementary Figure 5a,b). This strategy enables the basic use of BDPs for gene co-expression but is unfeasible for testing a library of BDPs.

Here, we use a cloning strategy based on the removal of a stuffer (placeholder) fragment via a type IIS RE [9] in combination with TA cloning [10] or Gibson assembly [11] (Supplementary Figure 5c) allowing RE site free, seamless cloning of a large number of BDPs. An expression cassette of the two genes of interest separated by a stuffer fragment is cloned into a starting vector using a single RE (Supplementary Figure 5b). In a subsequent cloning step the stuffer fragment is entirely cleaved out using a single type IIS RE (*Bmr*I) resulting in vector ends suitable for inserting PCR amplified BDPs Supplementary Figure 5c). *Bmr*I recognizes a non-palindromic sequence and cleaves in a variable sequence outside of its recognition sequence, as previously applied for a resection site free cloning approach (RSFC) [9]. We positioned the variable 3' overhang generated by *Bmr*I in the beginning of the start codon of the two genes, resulting in 3' thymidine overhangs on both sides of the vector (Supplementary Figure 5c). Adenine-tailed PCR fragments of BDPs can be directly cloned into the vector by TA cloning complementing the start codons.

Thereby, in total only two REs are needed for preparing the promoter library in the vector. This approach does not require RE digestion of the BDPs or the presence of MCSs in the vector and maintains the natural sequence context of the BDP up to the start codon. MCSs contain several RE sites adding non-natural sequences to the 5' untranslated region of the mRNA that can interfere with mRNA structure thereby causing translation inhibition [41]. The same library of BDPs can be used for cloning between any gene pairs. Alternatively overlaps and Gibson assembly can be used. However, in this case it is necessary to add overlaps to the GOIs to all promoters and new primers are needed for each gene pair to be co-expressed (extended discussion below). Random insertion of fragments by TA cloning is a major disadvantage for the cloning of MDPs or coding sequences as only the forward orientation is functional. For BDPs it is however a beneficial trait, since the same BDP can be tested in both orientations in a single cloning experiment.

In addition to dual gene co-expression (Supplementary Figure 5a-c), BDPs and BDTs (bidirectional terminators) facilitate also the assembly of multi gene expression cassettes in comparison to MDPs and MDTs (monodirectional terminators) (Supplementary Figure 5d). Typically the efficiency of overlap-directed DNA assembly methods is decreasing with the number of fragments in the assembly [42]. The number of parts (promoters and terminators) needed is approximately cut in half using BDPs and BDTs over MDPs and MDTs, considerably increasing the efficiency of multi fragment assemblies (depending on the method used [42]). In addition, cassettes based on the bidirectional elements reported here are shorter than using monodirectional elements. Smaller expression cassettes can be verified with less sequence reactions and show typically higher transformation efficiencies [43,44].

Using Gibson assembly or similar overlap directed cloning methods for the cloning of BDPs relying on identical core promoters.

Alternatively to RE based cloning, overlap-directed DNA assembly methods such as Gibson assembly [11], CPEC [45] or SLIC/SLiCE [46,47] can be used requiring overlapping regions (here referred to as 'cloning junctions') with the vector. Thus for each gene pair a new set of primers is needed to amplify the MDPs to be tested for fine-tuning co-expression.

Cloning by overlap based cloning strategies such as Gibson assembly requires identical sequences of the ends of the PCR products of the BDPs with the two genes of interest. In the library approach described in this paper, the BDPs are PCR amplified and then cloned into the vector. For each bidirectional promoter side tested, a separate primer would be needed using this approach, which would render the library amplification of a larger number of promoters rather expensive.
However, a large set of the BDPs described in this paper can be amplified with only two primers: The natural P_{HHX2} variants (32 BDPs, Fig. 2c,d) and the synthetic hybrid BDPs (31 BDPs, Fig. 4) contain identical core promoters. Hence, their ends are matching and they can be amplified with the same primers. These more than 60 promoters cover a wide range of expression levels, ratios, regulation and are exceptionally short, providing a high expression strengths per bp of promoter length (Fig. 5c).
For cloning of a larger number of constructs and the availability of suitable screening systems, TA cloning is favorable. Due to higher efficiencies and less errors in the cloning junctions, we recommend Gibson assembly/overlap based cloning if a small set BDPs is to be tested.

Supplementary data - see separate Excel and RAR files

Supplementary Data 1 – List of head to head, head to tail and tail to tail genes in *P. pastoris*

Supplementary Data 2 - List of BDPs & primers for cloning

Supplementary Data 3 - Annotated sequences for minimal set of promoters

Supplementary references

These supplementary references are self-contained. References mentioned in both the main text and the Supplementary Information are independently part of both reference lists.

1. Shaner, N. C. *et al.* Improved monomeric red, orange and yellow fluorescent proteins derived from Discosoma sp. red fluorescent protein. *Nat. Biotechnol.* **22,** 1567–72 (2004).
2. Vogl, T. & Glieder, A. Regulation of Pichia pastoris promoters and its consequences for protein production. *N. Biotechnol.* **30,** 385–404 (2013).
3. Vogl, T. *et al.* A Toolbox of Diverse Promoters Related to Methanol Utilization: Functionally Verified Parts for Heterologous Pathway Expression in Pichia pastoris. *ACS Synth. Biol.* **5,** 172–86 (2016).
4. Kranthi, B. V., Kumar, H. R. V. & Rangarajan, P. N. Identification of Mxr1p-binding sites in the promoters of genes encoding dihydroxyacetone synthase and peroxin 8 of the methylotrophic yeast Pichia pastoris. *Yeast* **27,** 705–11 (2010).
5. Hartner, F. S. *et al.* Promoter library designed for fine-tuned gene expression in Pichia pastoris. *Nucleic Acids Res.* **36,** e76 (2008).
6. Xuan, Y. *et al.* An upstream activation sequence controls the expression of AOX1 gene in Pichia pastoris. *FEMS Yeast Res.* **9,** 1271–82 (2009).
7. Miller, C. A., Martinat, M. A. & Hyman, L. E. Assessment of aryl hydrocarbon receptor complex interactions using pBEVY plasmids: expressionvectors with bi-directional promoters for use in Saccharomyces cerevisiae. *Nucleic Acids Res.* **26,** 3577–83 (1998).
8. Li, A. *et al.* Construction and characterization of bidirectional expression vectors in Saccharomyces cerevisiae. *FEMS Yeast Res.* **8,** 6–9 (2008).
9. Vogl, T., Ahmad, M., Krainer, F. W., Schwab, H. & Glieder, A. Restriction site free cloning (RSFC) plasmid family for seamless, sequence independent cloning in Pichia pastoris. *Microb. Cell Fact.* **14,** 103 (2015).
10. Mead, D. A., Pey, N. K., Herrnstadt, C., Marcil, R. A. & Smith, L. M. A universal method for the direct cloning of PCR amplified nucleic acid. *Biotechnology. (N. Y).* **9,** 657–63 (1991).
11. Gibson, D. G. *et al.* Enzymatic assembly of DNA molecules up to several hundred kilobases. *Nat Methods* **6,** 343–5 (2009).
12. Chen, S., Reger, R., Miller, C. & Hyman, L. E. Transcriptional terminators of RNA polymerase II are associated with yeast replication origins. *Nucleic Acids Res.* **24,** 2885–93 (1996).
13. Cregg, J. M., Barringer, K. J., Hessler, A. Y. & Madden, K. R. Pichia pastoris as a host system for transformations. *Mol. Cell. Biol.* **5,** 3376–85 (1985).
14. Orr-Weaver, T. L., Szostak, J. W. & Rothstein, R. J. Yeast transformation: a model system for the study of recombination. *Proc. Natl. Acad. Sci. U. S. A.* **78,** 6354–8 (1981).
15. Küberl, A. *et al.* High-quality genome sequence of Pichia pastoris CBS7435. *J Biotechnol* **154,** 312–20 (2011).
16. Trinklein, N. D. *et al.* An abundance of bidirectional promoters in the human genome. *Genome Res.* **14,** 62–6 (2004).
17. Liang, S. *et al.* Comprehensive structural annotation of Pichia pastoris transcriptome and the response to various carbon sources using deep paired-end RNA sequencing. *BMC Genomics*

13, 738 (2012).

18. Hesketh, A. R., Castrillo, J. I., Sawyer, T., Archer, D. B. & Oliver, S. G. Investigating the physiological response of Pichia (Komagataella) pastoris GS115 to the heterologous expression of misfolded proteins using chemostat cultures. *Appl. Microbiol. Biotechnol.* **97,** 9747–9762 (2013).

19. Xu, Z. *et al.* Bidirectional promoters generate pervasive transcription in yeast. *Nature* **457,** 1033–7 (2009).

20. Neil, H. *et al.* Widespread bidirectional promoters are the major source of cryptic transcripts in yeast. *Nature* **457,** 1038–42 (2009).

21. Chang, D. T.-H., Wu, C.-Y. & Fan, C.-Y. A study on promoter characteristics of head-to-head genes in Saccharomyces cerevisiae. *BMC Genomics* **13 Suppl 1,** S11 (2012).

22. Portela, R. M. C. *et al.* Synthetic Core Promoters as Universal Parts for Fine-Tuning Expression in Different Yeast Species. *ACS Synth. Biol.* **6,** 471–484 (2017).

23. Hecht, A., Endy, D., Salit, M. & Munson, M. S. When Wavelengths Collide: Bias in Cell Abundance Measurements Due to Expressed Fluorescent Proteins. *ACS Synth. Biol.* **5,** 1024–1027 (2016).

24. Vogl, T. *et al.* Methanol independent induction in Pichia pastoris by simple derepressed overexpression of single transcription factors. *Biotechnol. Bioeng.* **115,** 1037–1050 (2018).

25. Campbell, R. E. *et al.* A monomeric red fluorescent protein. *Proc. Natl. Acad. Sci. U. S. A.* **99,** 7877–82 (2002).

26. Blazeck, J. & Alper, H. S. Promoter engineering: recent advances in controlling transcription at the most fundamental level. *Biotechnol. J.* **8,** 46–58 (2013).

27. Inan, M. . . Studies on the alcohol oxidase (AOX1) promoter of Pichia pastoris. (University of Nebraska, 2000).

28. Kranthi, B. V., Kumar, R., Kumar, N. V., Rao, D. N. & Rangarajan, P. N. Identification of key DNA elements involved in promoter recognition by Mxr1p, a master regulator of methanol utilization pathway in Pichia pastoris. *Biochim. Biophys. Acta* **1789,** 460–8 (2009).

29. Sievers, F. *et al.* Fast, scalable generation of high-quality protein multiple sequence alignments using Clustal Omega. *Mol. Syst. Biol.* **7,** 539 (2011).

30. Huang, X. & Miller, W. A time-efficient, linear-space local similarity algorithm. *Adv. Appl. Math.* **12,** 337–357 (1991).

31. Perez-Pinera, P. *et al.* Synthetic biology and microbioreactor platforms for programmable production of biologics at the point-of-care. *Nat. Commun.* **7,** 12211 (2016).

32. Lin-Cereghino, G. P. *et al.* Mxr1p, a key regulator of the methanol utilization pathway and peroxisomal genes in Pichia pastoris. *Mol. Cell. Biol.* **26,** 883–97 (2006).

33. Wu, G. *et al.* Metabolic Burden: Cornerstones in Synthetic Biology and Metabolic Engineering Applications. *Trends Biotechnol.* **34,** 652–664 (2016).

34. van Arensbergen, J. *et al.* Genome-wide mapping of autonomous promoter activity in human cells. *Nat. Biotechnol.* **35,** 145–153 (2017).

35. Yan, C., Zhang, D., Raygoza Garay, J. A., Mwangi, M. M. & Bai, L. Decoupling of divergent gene regulation by sequence-specific DNA binding factors. *Nucleic Acids Res.* 1–12 (2015). doi:10.1093/nar/gkv618

36. Duttke, S. H. C. *et al.* Perspectives on Unidirectional versus Divergent Transcription. *Mol. Cell* **60,** 348–349 (2015).

37. Andersson, R. *et al.* Human Gene Promoters Are Intrinsically Bidirectional. *Mol. Cell* **60,** 346–347 (2015).

38. Duttke, S. H. C. *et al.* Human promoters are intrinsically directional. *Mol. Cell* **57,** 674–84 (2015).

39. Rhee, H. S. & Pugh, B. F. Genome-wide structure and organization of eukaryotic pre-initiation complexes. *Nature* **483,** 295–301 (2012).

40. Jin, Y., Eser, U., Struhl, K. & Churchman, L. S. The Ground State and Evolution of Promoter Region Directionality. *Cell* 1–10 (2017). doi:10.1016/j.cell.2017.07.006

41. Crook, N. C., Freeman, E. S. & Alper, H. S. Re-engineering multicloning sites for function and convenience. *Nucleic Acids Res.* **39,** e92 (2011).

42. de Kok, S. *et al.* Rapid and reliable DNA assembly via ligase cycling reaction. *ACS Synth. Biol.* **3,** 97–106 (2014).

43. Amendola, M., Venneri, M. A., Biffi, A., Vigna, E. & Naldini, L. Coordinate dual-gene transgenesis by lentiviral vectors carrying synthetic bidirectional promoters. *Nat. Biotechnol.* **23,** 108–16 (2005).

44. Xie, M., He, Y. & Gan, S. Bidirectionalization of polar promoters in plants. *Nat. Biotechnol.* **19,** 677–9 (2001).

45. Quan, J. & Tian, J. Circular polymerase extension cloning for high-throughput cloning of complex and combinatorial DNA libraries. *Nat. Protoc.* **6,** 242–51 (2011).

46. Li, M. Z. & Elledge, S. J. Harnessing homologous recombination in vitro to generate recombinant DNA via SLIC. *Nat. Methods* **4,** 251–6 (2007).

47. Zhang, Y., Werling, U. & Edelmann, W. SLiCE: a novel bacterial cell extract-based DNA cloning method. *Nucleic Acids Res.* **40,** e55 (2012).
